# Anti-tumor activity of all-trans retinoic acid in gastric-cancer: gene-networks and molecular mechanisms

**DOI:** 10.1186/s13046-023-02869-w

**Published:** 2023-11-11

**Authors:** Luca Guarrera, Mami Kurosaki, Silvio-Ken Garattini, Maurizio Gianni’, Gianpiero Fasola, Luca Rossit, Michele Prisciandaro, Maria Di Bartolomeo, Marco Bolis, Paola Rizzo, Claudia Nastasi, Marika Foglia, Adriana Zanetti, Gabriela Paroni, Mineko Terao, Enrico Garattini

**Affiliations:** 1 Department of Biochemistry and Molecular Biology, Istituto di Ricerche Farmacologiche Mario Negri IRCCS, via Mario Negri 2, Milano, 20156 Italy; 2 Department of Oncology, Academic Hospital of Udine ASUFC, Piazzale Santa Maria della Misericordia 15, Udine, 33100 UD Italy; 3 Department of General Surgery, Academic Hospital of Udine ASUFC, Piazzale Santa Maria della Misericordia 15, Udine, 33100 UD Italy; 4 Department of Medical Oncology, Fondazione IRCCS Istituto Nazionale Dei Tumori, Via Venezian 1, Milan, 20133 Italy; 5 Department of Oncology, Istituto di Ricerche Farmacologiche Mario Negri IRCCS, via Mario Negri 2, Milano, 20156 Italy; 6 Faculty of Biomedical Sciences, Institute of Oncology Research, USI, Bellinzona, 6500 TI Switzerland; 7 Istituto di Ricerche Farmacologiche Mario Negri IRCCS, Bergamo, 24100 Italy

**Keywords:** All-trans-retinoic-acid, Immune-responses, Gastric-cancer, IRF1, RNA-sequencing

## Abstract

**Background:**

Gastric-cancer is a heterogeneous type of neoplastic disease and it lacks appropriate therapeutic options. There is an urgent need for the development of innovative pharmacological strategies, particularly in consideration of the potential stratified/personalized treatment of this tumor. All-Trans Retinoic-acid (ATRA) is one of the active metabolites of vitamin-A. This natural compound is the first example of clinically approved cyto-differentiating agent, being used in the treatment of acute promyelocytic leukemia. ATRA may have significant therapeutic potential also in the context of solid tumors, including gastric-cancer. The present study provides pre-clinical evidence supporting the use of ATRA in the treatment of gastric-cancer using high-throughput approaches.

**Methods:**

We evaluated the anti-proliferative action of *ATRA* in 27 gastric-cancer cell-lines and tissue-slice cultures from 13 gastric-cancer patients. We performed RNA-sequencing studies in 13 cell-lines exposed to ATRA. We used these and the gastric-cancer RNA-sequencing data of the *TCGA*/*CCLE* datasets to conduct multiple computational analyses.

**Results:**

Profiling of our large panel of gastric-cancer cell-lines for their quantitative response to the anti-proliferative effects of ATRA indicate that approximately half of the cell-lines are characterized by sensitivity to the retinoid. The constitutive transcriptomic profiles of these cell-lines permitted the construction of a model consisting of 42 genes, whose expression correlates with *ATRA*-sensitivity.  The model predicts that 45% of the *TCGA* gastric-cancers are sensitive to ATRA. RNA-sequencing studies performed in retinoid-treated gastric-cancer cell-lines provide insights into the gene-networks underlying *ATRA* anti-tumor activity. In addition, our data demonstrate that ATRA exerts significant immune-modulatory effects, which seem to be largely controlled by *IRF1* up-regulation. Finally, we provide evidence of a feed-back loop between *IRF1* and *DHRS3*, another gene which is up-regulated by ATRA.

**Conclusions:**

ATRA is endowed with significant therapeutic potential in the stratified/personalized treatment gastric-cancer. Our data represent the fundaments for the design of clinical trials focusing on the use of ATRA in the personalized treatment of this heterogeneous tumor. Our gene-expression model will permit the development of a predictive tool for the selection of ATRA-sensitive gastric-cancer patients. The immune-regulatory responses activated by ATRA suggest that the retinoid and immune-checkpoint inhibitors constitute rational combinations for the management of gastric-cancer.

**Supplementary Information:**

The online version contains supplementary material available at 10.1186/s13046-023-02869-w.

## Background

Gastric-cancer is a heterogeneous neoplasia and one of the leading causes of tumor mortality worldwide [1, 2]. The frequency of stomach tumors is higher in the male than the female population (2/3 of the cases). The traditional classification of gastric-cancer defines 3 sub-groups, “*Intestinal*”, “*Diffuse*” and “*Mixed*” [3]. In addition, this tumor classifies into different sub-types according to the clinical-stage, histo-pathological features and mutational/trascriptomic profiles [4–7]. A recently developed gene-expression fingerprint gathers gastric-cancers into the *Genomic-intestinal* (*G-INT*) and *Genomic-diffuse* (*G-DIFF*) sub-groups [8]. Gastric-cancer lacks significant treatment options and innovative pharmacological strategies are needed [9], particularly in the context of the stratified/personalized treatment of this neoplastic disease.

All-Trans Retinoic-acid (ATRA) is the active metabolite of vitamin-A and it is the first example of clinically approved cyto-differentiating agent, being used in *Acute-Promyelocytic-Leukemia* (*APL*) [10]. ATRA-based therapeutic strategies induce long-lasting remissions in approximately 80% of the *APL* cases [11, 12]. This has stirred enthusiasm on the use of ATRA for the treatment of solid tumors, including gastric-cancer. Indeed, recent studies indicate that ATRA exerts significant anti-tumor effects in breast-cancer [13–16].

Here, we provide pre-clinical evidence on the anti-tumor effects exerted by ATRA in gastric-cancer, using multiple cell-lines, short-term tumor-tissue cultures and mouse xenografts. In addition, we generate a gene-expression model based on 42 genes, which predicts gastric-cancer responsiveness to ATRA. The RNA-sequencing (*RNA-seq*) studies performed in ATRA-treated cell-lines provide insights into the gene-networks underlying the action of the retinoid. In particular, we demonstrate that ATRA up-regulates *IRF1* and *DHRS3*, two genes whose silencing abrogates/reduces the anti-proliferative action of the retinoid. Finally, our data indicate that ATRA exerts a significant immune-modulatory action, resulting in increased antigen-presentation and interferon-dependent responses.

## Methods

### Chemicals, siRNAs and cell-lines

Chemicals: ATRA (Sigma-Aldrich, https://www.sigmaaldrich.com), AM580 (Tocris, http://www.tocris.com), BMS961 (Tocris), ER50891 (Tocris), CD2314 (Tocris), MM11253 (Tocris), ER50891 (Tocris) and CD2665 (Tocris). Stealth *Interfering-RNA*s (*RNAi*, Invitrogen-Thermo Fisher Scientific, Carlsbad, CA, USA): *si-IRF1a* = RNAi (HSS105500) and *si-IRF1b* = RNAi (HSS179960). RNAi Negative Control duplexes (high GC, cat. No. 12935-400) served as negative controls (*si-CTRL*). The characteristics/origin of the gastric-cancer cell-lines used is available in Table S1.

### Silencing and over-expression studies

To silence *IRF1* in a transient manner, *HGC-27* or *LMSU* cells were transfected with the *si-IRF1a*, *si-IRF1b* and *si-CTRL* stealth *RNAi* oligonucleotides (60 nM), in *OptiMem* containing *Lipofectamine-2000* (Invitrogen). To obtain the *shRNA* plasmid-constructs used for *IRF1* (*sh-IRF1a*/*sh-IRF1b*) and *DHRS3* (*sh-DHRS3a*/*sh-DHRS3b*) silencing and the non-targeted control-*shRNA* plasmid-constructs (*sh-CTRL1*/*sh-CTRL-2*), double-stranded DNA-oligonucleotides were designed by Biosettia Inc. (San Diego, CA) and synthesized by Metabion (Metabion international AG/metabion GmbH 2018). The *shRNA*-coding double-stranded DNAs were inserted into the *pGreenPuro*-plasmid (System Biosciences Inc, Palo Alto, CA), using the *EcoRI*/*BamHI* sites located downstream of the H1 gene promoter (double-stranded oligonucleotides structures: Table S2). The constructs were transfected in *293TN* cells to generate lentiviral particles, which were concentrated with *PEG-IT* (System-Biosciences, Palo Alto, CA) and used to infect *HGC-27* cells. Infected *HGC-27* cells were selected in 5 µg/ml puromycin (Sigma-Aldrich). *IRF1* over-expression studies in retinoid-resistant *AGS* cells: *AGS* cells were transiently transfected with a plasmid construct allowing the expression of the full-length *IRF1* cDNA (RC203500; Origene) using a standard approach, as detailed in the legend to Fig. S8.

### Western-blots, FACS analyses and cell-growth studies

Cell-lysates in *RIPA* buffer containing *PMSF*(1.0 mM)/1×protease-inhibitor were separated by *SDS–PAGE*, transferred to nitro-cellulose (Protran, Amersham) and incubated with primary-antibodies (1:1000) at 4 °C overnight. After staining with *HRP*-conjugated secondary-antibodies (1:10000 for anti-rabbit and 1:3000 for anti-mouse antibodies), signals were developed using the *ECL-Star* kit (Euroclone) and the *BioRad-Chemidoc-TM* imaging-system (Image LabTM touch software, BioRad). *β2-actin* served as the loading control. Western-blot experiments were conducted with anti-*IRF1* (8478, Cell Signaling Technology; E-AB-12,522, Elabscience), anti-*DHRS3* (FNab02372, FineTest, Wuhan, China) anti-*β2-actin* (SC-47,778, Santa Cruz) and anti-tubulin (T5168, Sigma-Aldrich) antibodies. In *FACS* (*Fluorescence-Activated-Cell-Sorter*) studies, samples were stained with Viakrome808 (Beckman Coulter, Miami, FL, USA) to exclude dead cells and then HLA-A/B/C monoclonal Ab (Biolegend, cat. 311,410) according to manufacturer’s instruction. Events were acquired with Cytoflex LX (Beckman Coulter, Miami, FL, USA). Flow cytometry data were analyzed using FlowJo™ v10.8 Software (BD Life Sciences). Cell growth studies were performed with the colorimetric *MTS*-assay kit (Abcam, ab197010), the Sulforhodamine assay [16] or an automatic counting of viable cells with the use of the *Vi-cell Blu* cell viability analyzer (Beckman-Coulter).

### Short-term tissue-slice cultures

The gastric-cancer samples used for the short-term tissue-slice cultures were obtained from patients undergoing a *Tru-cut* diagnostic procedure and they were supplied by *Academic-Hospital-of-Udine-ASUFC*, Udine, and *Fondazione-IRCCS-Istituto-Nazionale-Dei-Tumori*, Milano. All the procedures were approved by the ethical committees of the two clinical centers and a signed informed consent for the donation of the samples was obtained from patients. We performed tissue-slice cultures of the tumor samples (Supplementary Methods), as described [16]. For the quantitative histochemical determination of the *Ki67* protein, tumor slices were fixed, paraffin embedded, cut into 5 μm slices and stained for the *Ki67* protein using a specific antibody (Abcam, Ab16667). The number of *Ki67*-positive cells was counted under the microscope using the generated slides. We examined 5 fields/slide, each containing at least 1,000 cells/field.

### Studies involving experimental animals

Female athymic *Nude* mice (Charles River, Italy) were transplanted sub-cutaneously with the *LMSU* and *NCI-N87* cell-lines. All the experiments involving mice (project: 685/2020-PR) were performed following approval of the internal Ethical Committee on Animal Experimentation of the *Istituto-di-Ricerche-Farmacologiche-Mario-Negri-IRRCS*. Project 685/2020-PR was approved by the *Italian-Ministry-of-Health* and it was conducted in compliance with the Italian legislation.

### Calculation of the experimentally determined ATRA-score

Cell lines were exposed to increasing concentrations of ATRA (0.001–10.0 µM) for 3/6/9 days and cell-proliferation was determined with the colorimetric *MTS*-assay kit (Abcam, ab197010). To measure the sensitivity of each cell-line to ATRA anti-proliferative action, we calculated an experimental *ATRA-score* based on the *Area-Under*-*the*-*Curve* (*AUC*) determined for each cell-line exposed to ATRA for 6 days (Fig. S1). The calculated *AUC* values were re-scaled between the “1.00” and “0.00” reference-values.

### RNA-sequencing

*RNA-Seq* was performed with an Illumina NextSeq500 instrument using paired-end, 121-base-pair-long reads. The quality of the sequencing reads was evaluated with the *FastQC* (v.0.11.9) protocol. Sequence alignment of total-RNA (stranded) to the reference human genome (GRCh38) was performed with *STAR* (v.2.7.9a) [17], using a two-pass mode. We quantified gene-expression with the comprehensive annotations of *Gencode* (v38 GTF File). We adjusted and normalized samples for library size using the *cpm* method in the *R*-statistical-environment. Differential analysis was conducted with the *DESeq2* (v1.28.1) pipeline [18]. *GSEA* (*Gene-Set-Enrichment-Analysis*) was performed using the *Limma* (v. 3.52.2) package. Gene-set collections were retrieved from the *Molecular-Signature-Database* (*MSigDB*) [19]. We corrected the p-values for multiple testing using the *False-Discovery-Rate* (*FDR*) procedure, with the significance-threshold set at 0.1. The raw data are available in the *EMBL-EBI Annotare* database [20] (https://www.ebi.ac.uk/fg/annotare/) under the accession numbers: E-MTAB-12,387 (Cell-lines) and E-MTAB-12,385 (Patients). The processed *RNA-seq* data of our gastric-cancer cell-lines are available in Table S4. We utilized the processed/normalized *RNA-Seq* data to perform transcriptomic clustering of the cell-lines, using the *R*-programming language. We calculated the pairwise distances between samples using the Euclidean distance metric. To carry out hierarchical clustering, we used the above values as inputs for the *hclust* function to measure the distance between clusters with the *Ward’s minimum variance* method [21].

### In-silico computation of the ATRA-score fingerprint

To obtain a signature predicting the response to ATRA treatment in gastric-cancer, we correlated the basal gene-expression levels and the *ATRA-scores* of each cell-line, using the *Spearman’s* approach. We retrieved the constitutive transcriptomic data of the cell-lines used for the correlation analysis from the *CCLE* (*Cancer-Cell-Line-Encyclopedia*) database. The correlation analysis resulted in an initial list of genes (p-value < 0.01; *rho* correlation coefficient > 0.4), whose number was reduced with a connectivity analysis [22] involving the *STRING* (*Search-Tool-for-the-Retrieval-of-Interacting-Genes/Proteins*) database [23]. Selection of the genes showing a degree of interconnectedness ≥ 2 resulted in the final gene-expression signature.

### ATRA sensitivity predictions in gastric-cancer patients

We predicted the *ATRA-score* values of the *TCGA* (*The-Cancer-Genome-Atlas*) and our 13 gastric-cancer cases (*ATRA-scores* and tumor histological characteristics in Supplementary Methods), using a *Similarity-score* algorithm (*GSVA*: *Gene-Set-Variation-Analysis*) based on the gene-expression signature described above and a *Single-Sample-Gene-Set-Enrichment-Analysis* (*ssGSEA*) approach [24]. The *GSVA* algorithm [25] permitted the calculation of a *Similarity-score* value reflecting the enrichment of the gene-set under study, using the *GSVA*-*R*-package.

### Clustering of the samples into the G-DIFF and G-INT sub-groups

With the use of the signature template of 171 genes classifying gastric-cancers into the *G-DIFF* and *G-INT* sub-groups [8] and the *NTP* (*Nearest Template Prediction*) classification system [26], we clustered the 375 TCGA samples and our set of patients. We applied the *NTP* algorithm to the pre-processed data using the “*NTP*” R-package, which calculates the Euclidean distance between the expression profile of each sample and the centroid vector of each template, assigning the sample to the template characterized by the smallest distance. We evaluated the classification accuracy of the *NTP* method using a cross-validation approach (number of permutations = 100). With this approach, we split the data into a training- as well as a test-set in a random fashion and we assessed the performance according to the proportion of correctly classified samples in the test-set.

## Results

### A significant proportion of gastric-cancers are sensitive to ATRA

**Fig. 1:**
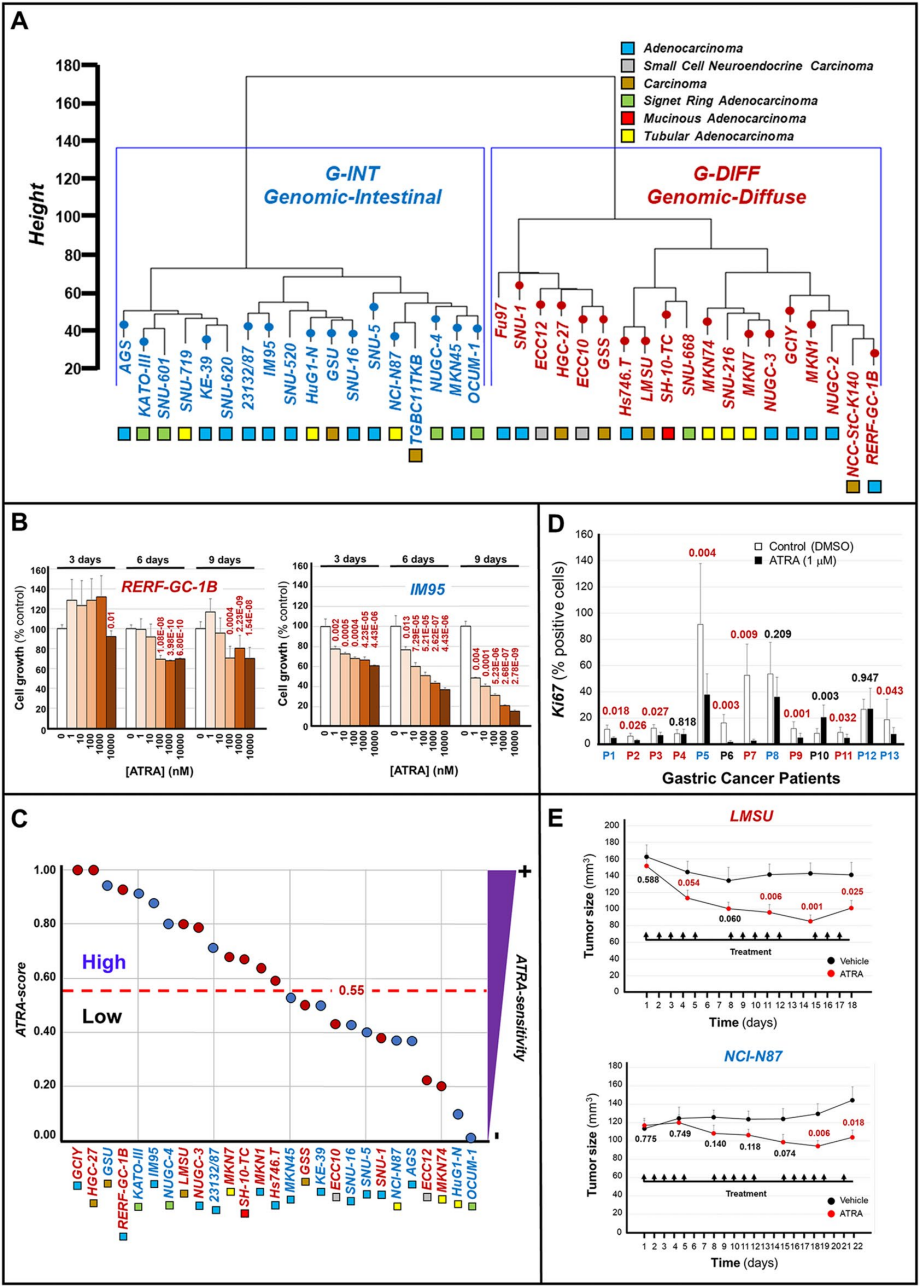
ATRA-dependent anti-tumor activity in gastric-cancer cell-lines, tissue-slice cultures of gastric-cancer specimens and LMSU/NCI-N87 mouse xenografts. **A** Unsupervised-hierarchical-clustering of the indicated gastric-cancer cell-lines based on the gene-expression profiles determined with the *RNA-seq* results (*TCGA*-dataset): cell-lines are classified according to transcriptomic-profile (*G-DIFF*=red; *G-INT*=blue) and histochemical-characteristics (colored-boxes). **B** ATRA growth-inhibitory effects in *RERF-GC-1B* (*G-DIFF*, red) and *IM95* (*G-INT*, blue) cell-lines: the values are normalized for the vehicle-treated controls, which are taken as 100%. Each point is the mean±SD of 10 cultures. When the cell-growth results observed in ATRA-treated specimens are significantly lower than those of the vehicle-treated counterparts, p-values (two-tailed Student’s t-test) are shown in red. **C** Gastric-cancer cell-lines are ranked according to the *ATRA-score* values: the bimodal and arbitrary threshold-value of 0.55 separates the cell-lines characterized by high ATRA-sensitivity (*ATRA-score* >0.55) from the cell-lines showing low ATRA-sensitivity (*ATRA-score* <0.55). Each calculated value is representative of at least 2 independent experiments. **D** Surgical specimens of 13 patients (P1-P13) were challenged with vehicle (DMSO) or ATRA (1.0 μM) for 48 hours: P3-P6-P9=female-patients; P1-P2-P4-P5-P7-P8-P10-P11-P12-P13= male-patients; *G-INT*-cases=blue; *G-DIFF*-cases=red; not-determined=black. Each value = Mean ± SE of 5 histological-fields/experimental-sample. The p-values (two-tailed Student's t-test) of the comparisons between the ATRA and corresponding vehicle-treated samples are shown. When the *Ki67* amounts observed in ATRA-treated specimens are lower than those of the vehicle-treated counterparts, the p-values are marked in red. **E** Mice were xenografted subcutaneously with 1×10^7^ *LMSU* or *NCI-N87* cells on both flanks. One week after transplantation, 10 animals/experimental group were treated with vehicle (DMSO) or ATRA (15.0 mg/kg) intra-peritoneally once/day, 5-days/week, as indicated (arrows). The calculated volumes of the tumors are plotted. As for *LMSU*, each point is the Mean ± SE of 15 and 19 tumors in the case of DMSO- and ATRA-treated animals, respectively. As for *NCI-N87*, each point is the Mean ± SE of 19 and 22 tumors. We compared the vehicle and ATRA-treated values at each time-point and the p-values (two-tailed Student’s t-test) of each comparison are shown

The heterogeneity of gastric-cancer [7, 27] is recapitulated by cell-lines whose constitutive gene-expression profiles are available in the *CCLE* (*Cancer-Cell-Lines-Encyclopedia*) database. This database contains RNA-sequencing (*RNA-seq*) data from 37 gastric-cancer cell-lines (Fig. 1A). According to their gene-expression profiles, these cell-lines classify into the *G-INT* and *G-DIFF* subgroups [8].

To define the sensitivity of gastric-cancer cells to ATRA growth-inhibitory action, we selected 27 cell-lines and exposed them to increasing concentrations of the retinoid (0.001-10µM). Subsequently, we evaluated the anti-proliferative effects of ATRA at day-3, day-6 and day-9, as exemplified in the case of the *RERF-GC* and *IM95* cells (Fig. 1B). Cell-lines were ranked for their sensitivity to the retinoid (Fig. 1C) using a modified version of the quantitative *ATRA-score* index [14, 16]. This new version of the *ATRA-score* is based on the *AUC*s determined for each retinoid-treated cell-line and it ranges from a minimal “0.00” value to a maximal “1.00” value. We calculated the *ATRA-scores* using the growth-curves at day-6 (Fig. S1). To obtain a bimodal distribution of the 27 gastric-cancer cell-lines based on their level of sensitivity to the retinoid, we used an arbitrary *ATRA-score* threshold value of 0.55, which falls above the median number of cell-lines. Indeed, this threshold value allows separation of the cell-lines into 2 unbiased groups, consisting of 14 and 13 cell-lines, which are characterized by high (*ATRA-score* > 0.55) and low (*ATRA-score* < 0.55) ATRA-sensitivity (Fig. 1C). The 2 most sensitive (*GCIY*/*HGC-27*) and the 2 least sensitive (*HuG1-N*/*OCUM-1*) cell-lines have a *G-DIFF* and a *G-INT* phenotype, respectively. This reflects the trend observed in our panel of cell-lines, which shows a respective enrichment of the *G-DIFF* and *G-INT* phenotypes in the high ATRA-sensitivity (9/14) and the low ATRA-sensitivity (8/13) groups.

To validate the results obtained with cell-lines, we evaluated the anti-proliferative effects exerted by ATRA in gastric-cancer tissue-slice cultures [16, 28] from 13 patients (Table S4, patients-characteristics). Five and 6 of these tumors classify as *G-INT* and *G-DIFF*, respectively, according to the constitutive gene-expression profiles determined by the *RNA-seq* analyses performed on 11 cases. We challenged tissue-slices with vehicle or ATRA for 48 h and the growth-inhibitory action of the retinoid was determined by quantitative immune-histochemical measurement of the *Ki-67* proliferation-marker, as exemplified for patient-1 (*G-INT*) and patient-2 (*G-DIFF*) (Fig. S2). The results demonstrate that ATRA causes a decrease of the *Ki-67* levels in 9 cases (*G-DIFF* = 5 cases; *G-INT* = 3 cases; *NotDefined* = 1 case) (Fig. 1D).

To support the therapeutic potential of ATRA in gastric-cancer, we performed *in-vivo* studies with xenografts of a *G-DIFF* (*LMSU*/*ATRA-score* = 0.80) and a *G-INT* (*NCI-N87*/*ATRA-score* = 0.37) cell-line. Mice were administered vehicle (DMSO) or ATRA intra-peritoneally for 2/3 weeks and the tumor-size was determined at different time-points (Fig. 1E). As for *LMSU*, an ATRA-dependent reduction of the tumor-mass is already evident following 5 days of treatment and the decrease is maximal at day-15 (Fig. 1E, upper). In *NCI-N87* xenografts, the maximal effect of ATRA is of lower magnitude and it is delayed, being observed only at day-19 (Fig. 1E, lower). In these conditions, ATRA is devoid of systemic toxicity, as it exerts no significant effect on the body-weight of *LMSU*- and *NCI-N87*-transplanted mice (Fig. S3).

### Involvement of RARα in the anti-proliferative action of ATRA

Six retinoid-receptors (*RARα*/*RARβ*/*RARγ*/*RXRα*/*RXRβ*/*RXRγ*) are known and the active forms of these transcription-factors consist of *RAR*/*RXR* heterodimers, in which *RAR* acts as the ligand-binding component [29, 30]. ATRA is a pan-*RAR* agonist, binding/trans-activating *RARα*/*RARβ*/*RARγ* with equal affinity/efficiency.

**Fig. 2:**
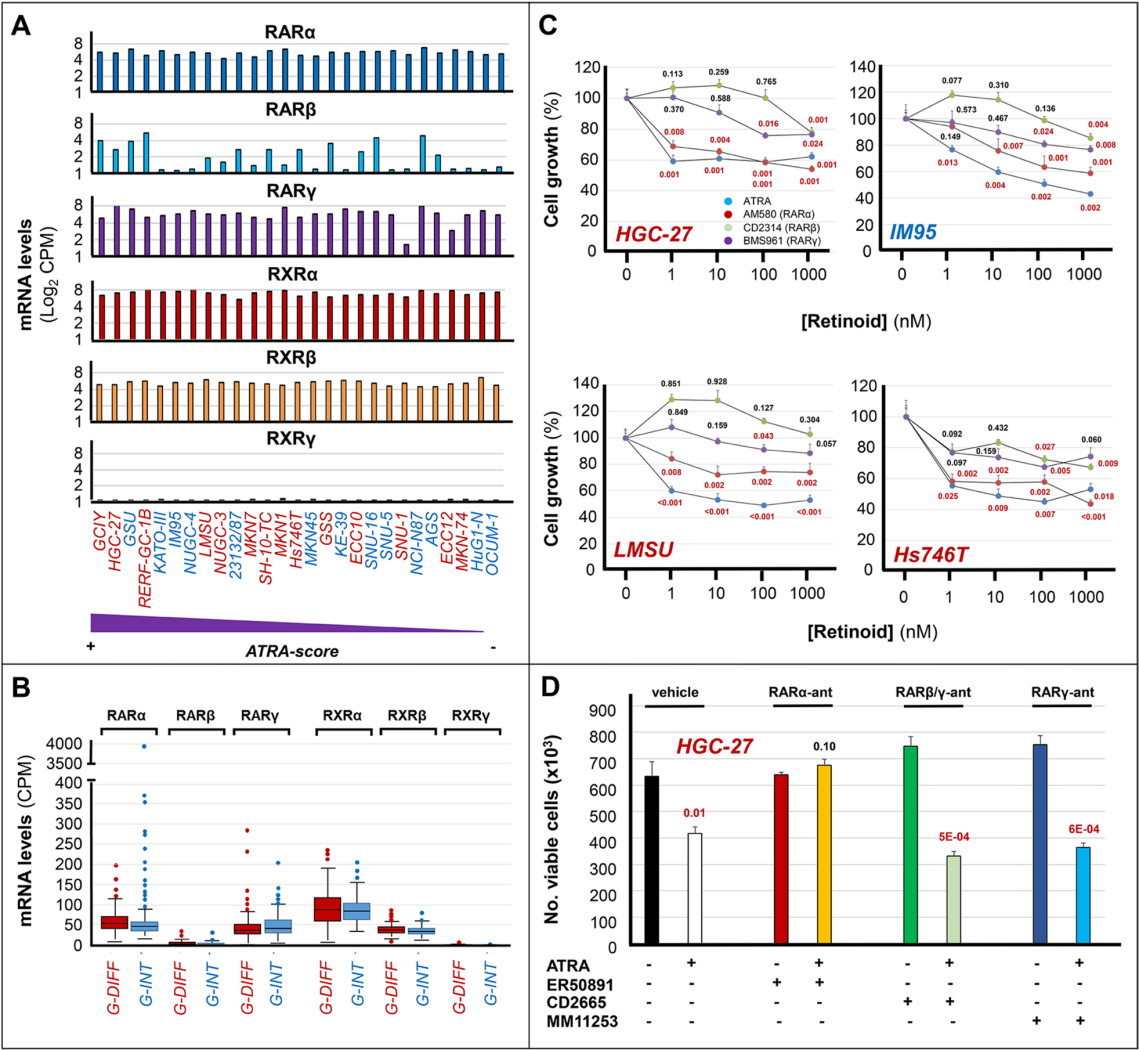
RAR and RXR mRNAs expression and RAR agonists anti-proliferative effects in gastric-cancer. **A** The panel shows the constitutive expression levels of the mRNAs coding for the indicated *RAR* and *RXR* isoforms in our panel of 27 gastric-cancer cell-lines. The cell-lines are ranked according to their decreasing sensitivity to the anti-proliferative effects of ATRA from left to right, as indicated. The expression values of the *RAR* and* RXR* mRNAs in gastric-cancer cell-lines are calculated using the *RNA-seq* results of the *CCLE* database. The values are expressed as Log 2 [CPM (Counts Per Million)]. *G-DIFF* and *G-INT* cell-lines are marked in red and blue, respectively. **B** The box plots indicate the constitutive expression levels of the mRNAs coding for the indicated *RAR* and *RXR* isoforms in gastric-cancer tissues characterized by a *G-DIFF* (red) or a *G-INT* (blue) phenotype. The values are calculated with the *RNA-seq* results of the *TCGA* database. The results are expressed as the Median [CPM] values  ±  SD. **C** The indicated *G-DIFF* (*HGC-27*; *LMSU*; *Hs746T*) and *G-INT* (*IM95*) cell-lines were exposed to vehicle (DMSO; [Retinoid] = 0 nM) or the indicated concentrations of the pan-*RAR* agonist, ATRA, the *RARα* agonist, AM580, the *RARβ* agonist, CD2314, and the *RARγ* agonist, BMS961, for 6 days. At the end of the treatment, the growth of each cell line was evaluated with the MTS assay. Each value is the Mean  ±  SD of 5 independent cultures and the data are normalized for the growth value of vehicle-treated cells (100%). We compare each compound-treated sample with the corresponding vehicle-treated counterparts at the different concentrations of the compounds. In case of statistical significance (two-tailed Student’s t-test), the p-values are shown in red. When the p-values lack statistical significance, they are marked in black. **D** Three independent cultures of *HGC-27* cells were exposed to vehicle (DMSO) the *RARα* antagonist, ER50891 (0.1 µM), the *RARβ /γ* antagonist, CD2665 (0.1 µM), or the *RARγ* antagonist, MM11253 (0.1 µM), in the absence and presence of ATRA (0.01 µM) for 9 days. At the end of the treatment, the number of viable cells was counted automatically. In all samples, cell viability was always  ≥  85%. The values indicated by the columns are the Mean  ±  SD of the 3 independent cultures considered. We compare each ATRA, ATRA + ER50891, ATRA + CD2665 and ATRA + MM11253 treated sample with the corresponding vehicle, ER50891, CD2665 and MM11253 treated counterparts. In case of statistical significance (two-tailed Student’s t-test), the *p*-values shown above the corresponding columns are marked in red

To identify the *RAR* isoform(s) underlying the anti-proliferative action of the retinoid, initially, we evaluated the constitutive expression of *RARα*, *RARβ*, *RARγ*, *RXRα*, *RXRβ* and *RXRγ* mRNAs in our gastric-cancer cell-lines (Fig. 2A). The *CCLE RNA-seq* data indicate that all the cell-lines express similar levels of *RARα*, *RARγ*, *RXRα* and *RXRβ* mRNAs. In contrast, the levels of *RARβ* mRNA are variable, although the majority of cell-lines expresses barely detectable amounts of the transcript. No correlation between *RARβ* mRNA levels and ATRA-sensitivity or *G-DIFF*/*G-INT* phenotype is evident. Moreover, no cell-line expresses detectable amounts of the *RXRγ* mRNA. Noticeably, the *RAR*/*RXR* expression profiles of the cell-lines recapitulate the situation of primary gastric-cancers, as indicated by the *RNA-seq* data of the *TCGA* database (Fig. 2B).

Subsequently, we took a pharmacological approach involving the use of *RARα*/*RARβ*/*RARγ* agonists. With this approach, we determined the growth of the *HGC-27* (*ATRA-score* = 1.00), *IM95* (*ATRA-score* = 0.88), *LMSU* (*ATRA-score* = 0.80) and *Hs747T* (*ATRA-score* = 0.59) cell-lines, which belong to the high ATRA-sensitivity group. To this purpose, we exposed the 4 cell-lines to increasing concentrations of ATRA, AM580 (*RARα* agonist) [31], CD2314 (*RARβ* agonist) [32] and BMS961 (*RARγ* agonist) [16] for 6 days (Fig. 2C). In all the cell-lines, AM580 is the sole *RAR* agonist causing a concentration-dependent growth-inhibitory action of the same order of magnitude as the one observed with ATRA. Indeed, CD2314 and BMS961 exert only marginal effects on the growth of *HGC-27*, *LMSU*, *IM95* and *Hs747T* cells and these effects are observed only with the highest concentration(s) of the two compounds. The data obtained with the *RAR* agonists support the idea that *RARα* is the primary retinoid receptor involved in the anti-proliferative action exerted by ATRA in gastric cells.

Finally, we performed experiments aimed at validating the hypothesized role of *RARα* in the growth inhibitory action exerted by ATRA, with the use of a specific *RARα* antagonist (ER50891), a *RARβ/γ* antagonist (CD2665) and a selective *RARγ* antagonist (MM11253) (Fig. 2D). To conduct these studies, we exposed *HGC-27* cells to ATRA, ER50891, CD2665 and MM11253 as well as the combinations of ATRA + ER50891, ATRA + CD2665 and ATRA + MM11253 for 9 days. In these experimental conditions, ATRA causes the expected growth inhibitory effect, while ER50891, CD2665 and MM11253 are devoid of any anti-proliferative action on *HGC-27* cells. Remarkably, the *RARα* antagonist, ER50891, is the sole compound suppressing the growth inhibitory action of ATRA. Indeed, exposure of *HGC-27* cells to ATRA + CD2665 and ATRA + MM11253 results in the same level of growth inhibition observed with ATRA alone.

In conclusion, the data obtained in *HGC-27* cells with a pharmacological approach based on the use of *RAR* agonists and antagonists are consistent with the idea that activation of *RARα* is necessary and sufficient to mediate the anti-proliferative activity of ATRA in sensitive gastric-cancer cells.

### Networks of genes whose constitutive expression is associated with ATRA-sensitivity

**Fig. 3:**
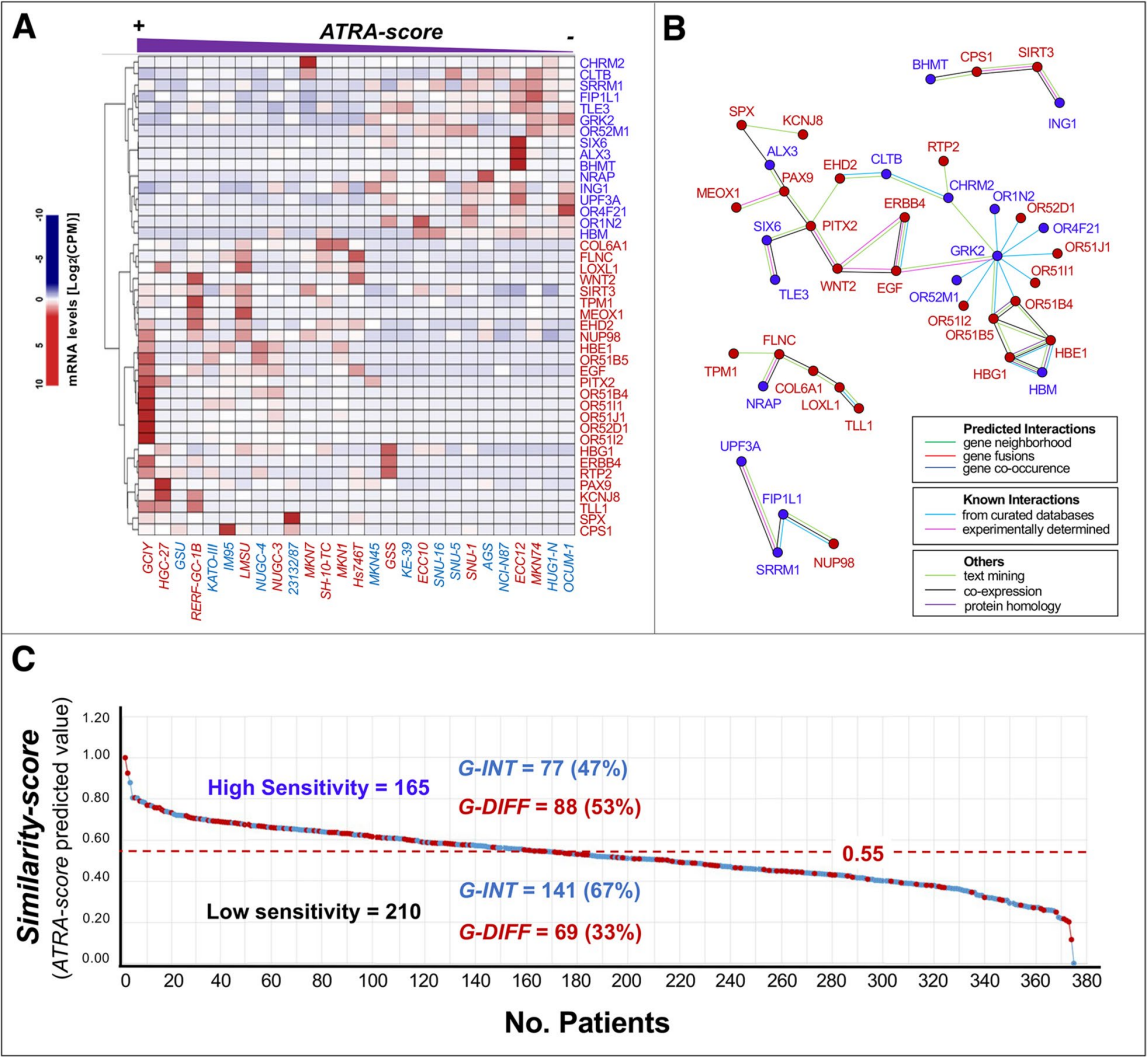
Transcriptomic model based on genes whose basal expression is associated with gastric-cancer cell-lines ATRA-scores. **A** The panel shows a heat-map illustrating the levels of the 42 genes whose constitutive expression is quantitatively associated with the ATRA-score values of the gastric-cancer cell-lines profiled for their sensitivity to ATRA. The mRNAs directly (high-Basal-Expression-Levels/high-ATRA-scores) and inversely (low-Basal-Expression-Levels/high-ATRA-scores) correlated with ATRA-sensitivity are marked in red and blue respectively. The expression values of the 42 mRNAs in gastric-cancer cell-lines are calculated with the use of the RNA-seq results available in the *CCLE* (*Cancer Cell Line Encyclopedia*) database. The values are expressed as Log 2 [CPM (Counts Per Million)]. The *G-DIFF* and *G-INT* cell-lines are marked in red and light-blue, respectively. **B** The panel illustrates the results of a *STRING* (*Search-Tool-for-the-Retrieval-of-Interacting-Genes/Proteins*) analysis performed on the 42 gene-products directly or inversely associated with ATRA-sensitivity. The genes directly and inversely associated with ATRA-sensitivity are marked by red and blue dots, respectively. **C** The panel shows the level of ATRA-sensitivity predicted in the 375 gastric-cancer samples of the TCGA dataset for which RNA-seq results are available. The predictions rest on the 42-gene model shown in panels (**A**) and (**B**) and they were generated with the use of a quantitative Similarity-score applied to the *RNA-seq* data. The *ATRA-score* threshold value of 0.55 is used to separate the cases predicted to be characterized by high ATRA-sensitivity (≥  0.55) and low ATRA-sensitivity (< 0.55). The *G-DIFF* and *G-INT* cases are marked in red and blue, respectively. The number and percentage of *G-DIFF* and *G-INT* cases observed in the high ATRA-sensitivity and low ATRA-sensitivity groups are indicated

As a first step towards the generation of a predictive tool for the selection of ATRA-sensitive gastric-cancer patients, we defined the constitutive gene-expression profiles of our panel of 27 cell-lines, employing the *CCLE*/*RNA-seq* data. The computational approach used identifies a limited number of transcripts whose basal expression levels correlate directly or inversely with the experimentally determined *ATRA-score* values in a quantitative manner (Fig. 3A). Indeed, 26 and 16 protein-coding mRNAs correlate with ATRA-sensitivity directly (high-*Basal-Expression-Levels*/high-*ATRA-score*s) and inversely (low-*Basal-Expression-Levels*/high-*ATRA-score*s). According to an analysis performed with the *STRING* database [23], the products of these 42 genes converge into 4 distinct networks of interacting proteins (Fig. 3B). The largest network (28 elements) contains proteins involved in tissue-development (*PITX2/PAX9/ALX3/MEOX1/SIX6/TLE3*) and the *WNT* pathway (*WNT2/TLE3/EGF/ERBB3*). The second network (6 elements) encloses proteins playing a role in myogenesis and collagen-homeostasis (*TPM1/FLNC/NRAP/COL6A1/LOXL1/TLL1*). The third and fourth networks consist of factors controlling pre-mRNA-processing (*UPF3A/SRRM1/FIP1L1/NUP98*) and metabolic-/mitochondrial-homeostasis (*CPS1*/*BHMT*/*SIRT3*/*ING1*).

We used the combined expression levels of the 42 transcripts to calculate *Similarity-score* values from the *RNA-seq* of the 375 gastric-cancer cases available in the *TCGA-*dataset. These *Similarity-scores* were utilized to predict the corresponding *ATRA-score* values (Fig. 3C). Indeed, we predict an *ATRA-score* ≥ 0.55 in 165 cases (high ATRA-sensitivity) and an *ATRA-score* < 0.55 in the remaining 210 cases (low ATRA-sensitivity). Thus, consistent with the proportion of gastric-cancer cell-lines experimentally responsive to ATRA (52%; Fig. 1C), 44% of gastric-cancers are predicted to be characterized by high ATRA-sensitivity. As expected from the cell-lines data, the *G-INT* phenotype is prevailing in patients characterized by predicted low ATRA-sensitivity (67%; Fig. 3C).

### Effects of ATRA on gene-expression in gastric-cancer cell-lines

**Fig. 4:**
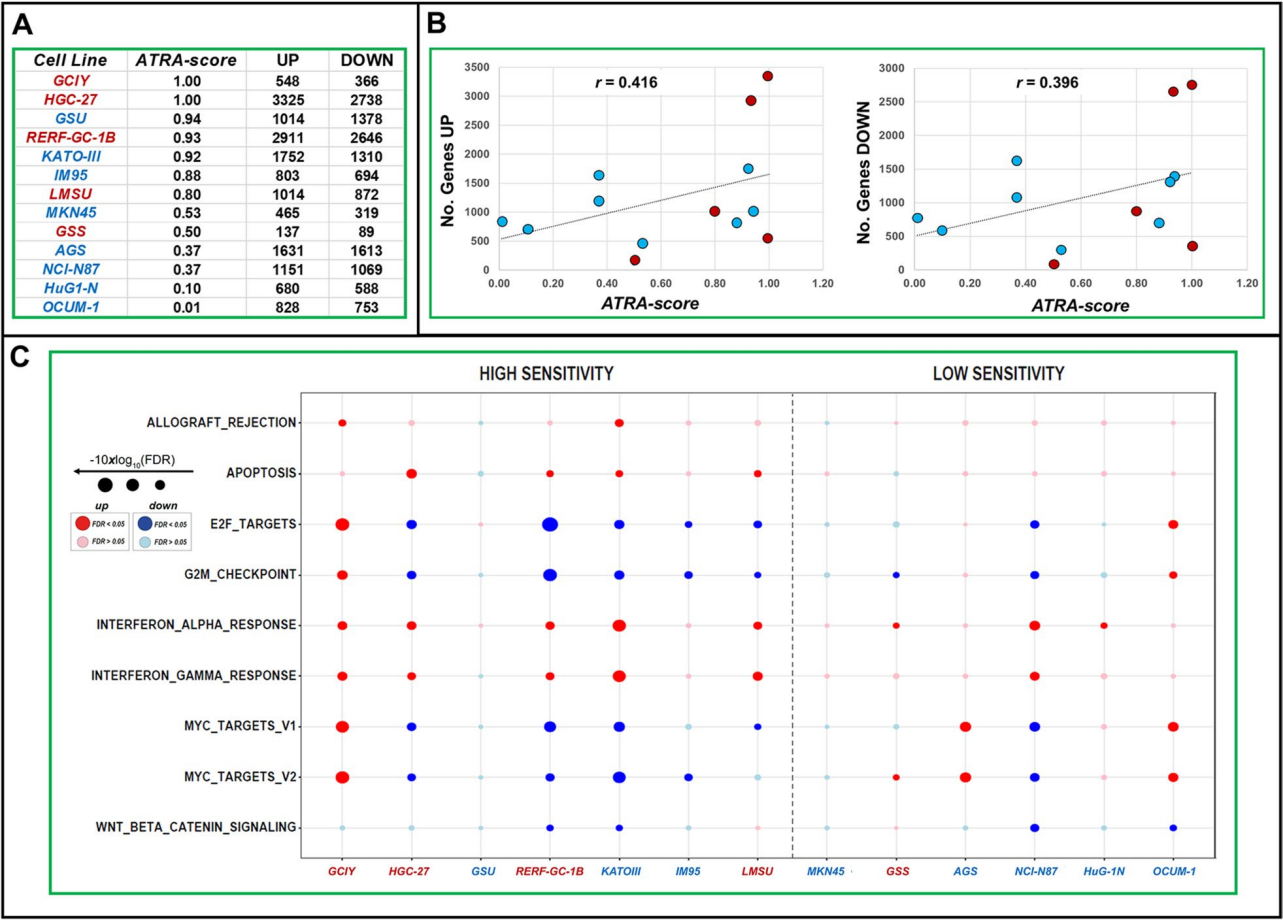
Effects of ATRA on the gene-expression profiles of gastric cancer cells: RNA-seq pathway analysis. Exponentially growing triplicate cultures of the indicated cell lines were exposed to ATRA (1.0 µM) for 48 hours. At the end of the treatment cells were subjected to *RNA-seq* analysis (processed data in Table S4). **A** The panel shows the ATRA-sensitivity scores (*ATRA-scores*) of the 13 gastric cell-lines considered along with the number of genes significantly (FDR<0.1) up-regulated (UP) and down-regulated (DOWN) by ATRA (1.0µM) following 48 hours of exposure. **B** The diagram illustrates the correlations between the *ATRA-score* values and the number of genes up-regulated by the retinoid in our experimental conditions. The *r*-correlation values are indicated. **C** The *RNA-seq* data were subjected to pathway analysis using the HALLMARK data set. The Figure illustrates a Dot-Plot of the most significant HALLMARK pathways which are up-regulated (red dots) and down-regulated (blue dots) by ATRA in the indicated cell-lines. The size of the dots is inversely proportional to the *FDR* (*False-Discovery-Rate*) values calculated. When the *FDR* values are <0.1, they are considered to be statistically significant. The dots shown in dark color are statistically significant, while those shown in light color lack significance. Only the most relevant up- (red) or downregulated (blue) pathways are illustrated. The full results of the analysis are available in Fig. S4

To obtain insights into the gene-networks mediating the action of ATRA in gastric-cancer, we performed *RNA-seq* studies in 13 of our cell-lines (Table S4). Seven and 6 cell-lines are characterized by high (*ATRA-score* > 0.55) and low (*ATRA-score* < 0.55) sensitivity to the retinoid, respectively (Fig. 4A). To perform these *RNA-seq* studies, we exposed each cell-line to vehicle or ATRA (1.0 µM) for 48 h, a time-interval preceding any sign of growth-inhibition. The number of transcripts up- and down-regulated by ATRA in each cell-line (Fig. 4A) shows a direct correlation with the *ATRA-score*s, as indicated by the linear-regression *r*-values (Fig. 4B). Indeed, the higher is the *ATRA-score* determined in each cell-line, the higher is the number of mRNAs up-/down-regulated by ATRA.

To gather further information regarding the pathways regulated by ATRA in the responsive cell-lines, we performed *GSEA* on the *RNA-seq* data, using the HALLMARK platform (Figs. 4C and S4). The ATRA-dependent anti-proliferative effects are associated with a decrease in the expression of genes involved in the control of the *G2M* checkpoint and genes regulated by the *E2F* transcription-factor. In the retinoid-sensitive cell-lines, the growth-inhibitory action of ATRA seems to involve a specific down-regulation of *c-myc* target-genes (Figs. 4C and S4). In the majority of the cell-lines, regardless of their sensitivity to the retinoid, ATRA increases the expression of genes controlled by interferon-α (*IFN*α) and interferon-γ (*IFN*γ) (Figs. 4C and S4). These data indicate that the retinoid activates a series of *IFN*-dependent immune-responses, which may be necessary but insufficient for the anti-proliferative effects of ATRA in gastric-cancer cells. As for the potential metabolic pathways involved in the growth-inhibitory action of ATRA, the *KEGG-Metabolism* platform indicates that ATRA tends to up-regulate genes controlling glycerophospholids and retinol metabolism in all cell-lines (Fig. S5).

**Fig. 5:**
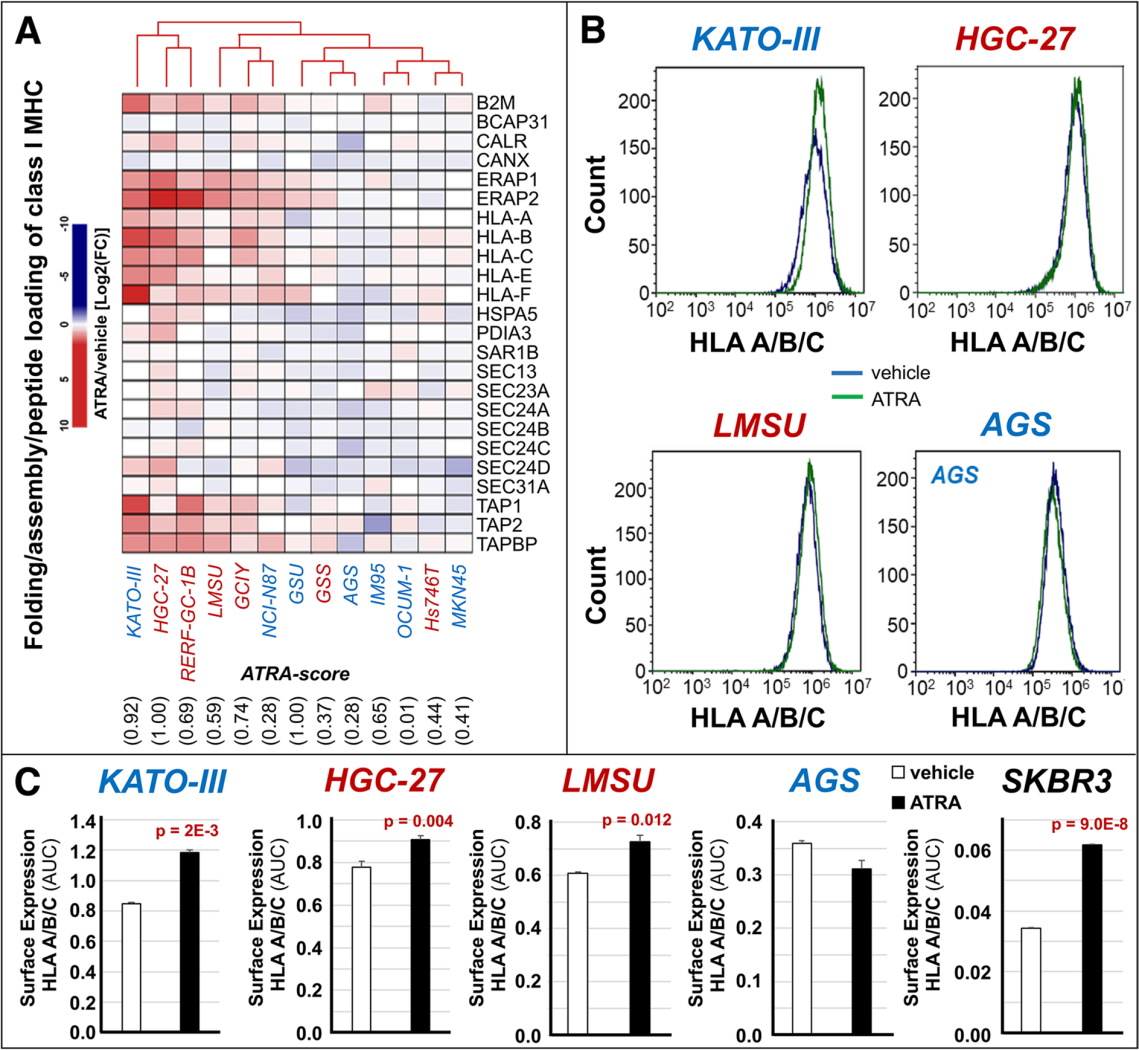
Effects of ATRA on the process of antigen-presentation in gastric-cancer cell-lines. The indicated cell-lines were exposed to vehicle (DMSO) or ATRA (1µM) for 48 hours. At the end of the treatment, each cell-line was subjected to *RNA-seq* analysis. **A** The panel shows a heat-map illustrating the effects of ATRA on the expression levels of the 24 genes constituting the “*Folding-Assembly-and-Peptide-Loading-of-Class-I-MHC*” REACTOME gene-network in the indicated gastric-cancer cell-lines. The results are expressed as the ATRA-vehicle ratio [Log_2_FC (Fold-Change)]. The *G-INT* cell-lines are marked in blue and the *G-DIFF* cell-lines are marked in red. The cell-lines *ATRA-score* values are shown below the heat-map, as indicated. **B** and **C** The indicated gastric-cancer and the control *SKBR3* breast cancer cell-lines were exposed to vehicle (DMSO) or ATRA (1µM) for 48 hours. At the end of the treatment, the cell-lines were subjected to *FACS* (*Fluorescence-Activated-Cell-Sorter*) analysis with an anti-*HLA/B/C* antibody. Panel **B** shows representative *FACS* graphs obtained with the indicated gastric-cancer cell-lines. Panel **C** shows the calculated *FACS* quantitative data. The data are expressed as the Mean+SD (*N*=3) of the *AUC* (*Area Under the Curve*) values determined from the *FACS* graphs. The surface expression of *HLA/B/C* was compared in each of the indicated vehicle-treated and ATRA-treated cell-lines. In case of significance (two-tailed Student’s t-test), the *p*-values of the comparisons are shown

ATRA-dependent up-regulation of the genes involved in *IFN*-dependent immune-responses is accompanied by an increase in the HALLMARK “*Allograft-Rejection*” gene-set (Figs. 4C and S4). This HALLMARK network contains genes involved in antigen-presentation and T-cell dependent suppression of tumor-growth/metastatic-spread, which suggests that ATRA increases the immunogenicity of gastric-cancer cells. Thus, we took into consideration the *REACTOME* “*Folding-Assembly-and-Peptide-Loading-of-Class-I-MHC*” gene-set, which consists of 24 genes playing key roles in antigen-presentation. ATRA up-regulates this gene-network in the majority of our gastric-cancer cell-lines, regardless of retinoid-sensitivity and *G-DIFF*/*G-INT* phenotype (Fig. 5A). In particular, ATRA stimulates the expression of the *HLA-A/B/C* and the *B2M* genes whose products are components of the *Major-Histocompatibility-Complex* (*MHC*). To evaluate whether these ATRA-dependent transcriptomic effects translate into an increase of the *HLA-A/B/C* surface-antigens, we performed *FACS* (*Fluorescence-Activated-Cell-Sorter*) analyses in the gastric-cancer *HGC-27*, *LMSU*, *KATO-III*, *AGS* and the breast-cancer *SKBR3* (positive control) cell-lines, using an anti-*HLA-A/B/C* antibody (Fig. 5B and C). Consistent with the general up-regulation of the *REACTOME* gene-network, *HGC-27*, *LMSU* and *KATO-III* cells show an ATRA-dependent increase in *HLA-A/B/C* surface-expression. Significantly, ATRA does not alter *HLA-A/B/C* surface-expression in *AGS*, the only cell-line showing a slight down-regulation of the *REACTOME* gene-network.

### Genes commonly modulated by ATRA in both *G-INT* and *G-DIFF* cell-lines

**Fig. 6:**
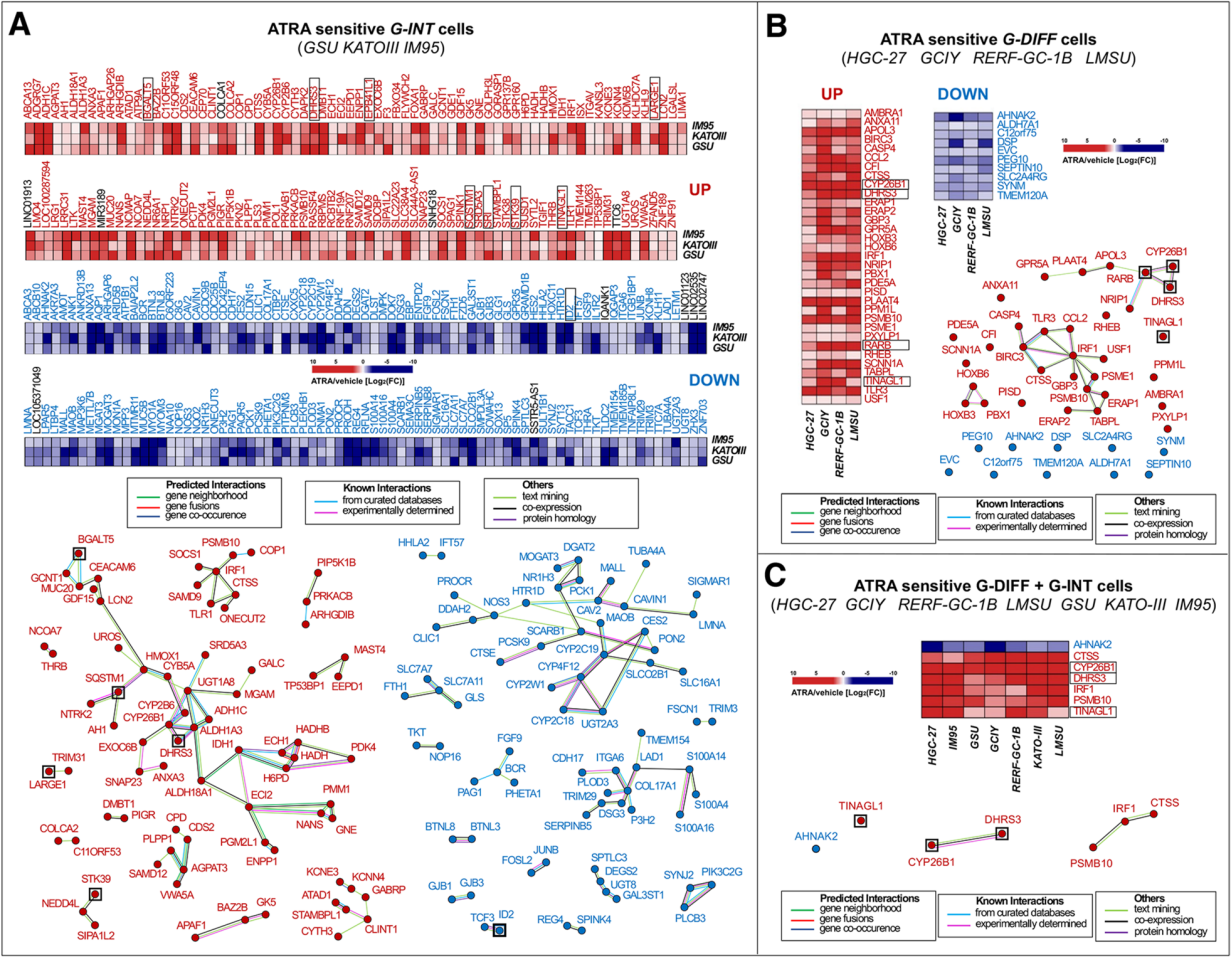
Gene-networks modulated by ATRA in retinoid-sensitive *G-INT* and *G-DIFF* gastric-cancer cell-lines. The *G-INT*/ATRA-sensitive *GSU*/*KATO-III*/*IM95* and the *G-DIFF*/ATRA-sensitive *HGC-27*/*GCIY*/*RERF-GC-1B*/*LMSU* cell-lines were exposed to vehicle (DMSO) or ATRA (1µM) for 48 hours and subjected to *RNA-seq* analysis. **A** Upper: heat-maps illustrating the effects of ATRA on the expression of the 297 genes commonly up-regulated (143 genes; UP) and down-regulated (154 genes; DOWN) in the 3 *G-INT* cell-lines (FDR < 0.1). The results (Mean of 3 independent vehicle-treated and ATRA-treated cultures) are expressed as the ATRA-vehicle ratio [Log_2_ *FC* (*Fold Change*)]. Up-regulated/genes=red; Down-regulated/genes=blue; Non-protein-coding/genes=black. Lower: *STRING* (*Search-Tool-for-the-Retrieval-of-Interacting-Genes*/*Proteins*) analysis of the 143 up-regulated gene-products (left diagram; red dots) and the 154 down-regulated gene-products (right diagram; blue dots). The proteins in black squares are encoded by genes up-regulated and down-regulated in the retinoid-resistant *G-DIFF* cell-lines, *AGS*, *NCI-N87*,* HuG1-N*, *MKN45*, *OCUM-1*. **B** Upper: 2 heat-maps illustrating the effects of ATRA on the expression levels of the 43 genes commonly (FDR < 0.1) up-regulated (33 genes; UP) and down-regulated (10 genes; DOWN) in the in the 4 *G-DIFF* gastric-cancer cell-lines. The results are expressed as the ATRA-vehicle ratio [Log_2_ *FC* (*Fold Change*)] and they represent the mean of 3 independent vehicle-treated and ATRA-treated cultures. Lower: *STRING*-analysis of the 33 up-regulated gene-products (red-dots) and the 10 down-regulated gene products (blue-dots). The proteins in black squares are encoded by genes up-regulated in the retinoid-resistant *G-DIFF* cell-line, *GSS*. **C** Upper: heat-map illustrating the effects of ATRA on the expression of the genes commonly up-regulated (6 genes; UP) and down-regulated (1 gene; DOWN) in ATRA-sensitive *G-INT*/*G-DIFF* cell-lines (FDR < 0.1). The results (mean of 3 vehicle-treated and ATRA-treated cultures) are expressed as the ATRA-vehicle ratio [Log_2_ *FC* (*Fold Change*)]. Lower: *STRING*-analysis of the 6 up-regulated gene products (upper diagram; red dots) and the single down-regulated gene product (lower diagram; blue dots) which are common to the 7 ATRA-sensitive *G-DIFF* and *G-INT* cell-lines shown in panels **A** and **B**. The type of protein interactions available in the literature are shown in the rectangular boxes

To identify genes potentially involved in the anti-tumor action of ATRA in *G-INT* tumors, we performed *RNA-seq* studies with 8 *G-INT* cell-lines exposed to ATRA for 48 h. In a first set of experiments, we focused on the *G-INT* cell-lines, *GSU*, *KATO-III* and *IM95* (*ATRA-score* > 0.55) characterized by high ATRA-sensitivity. In these cell-lines, ATRA modulates the expression of a few thousands transcripts (Fig. S6, left). However, only 143 and 154 common mRNAs are up-regulated and down-regulated by ATRA, respectively, in all these cell-lines (Fig. 6A).

As for the commonly up-regulated mRNAs, a *STRING* analysis of the data demonstrates that some of the corresponding translated products are part of 12 separate interactomes (Fig. 6A, lower-left). The largest interactome (37 elements) contains proteins involved in lipid-metabolism (*HADHB*/*HADH*/*ECH1*/*ECI2*/*ALDH1A3*/*ADH1C*/*DHRS3*/*CYP26B1*/*CYP2B6*/*UGT1A8*/*SRD5A3*/*GALC*/*BGALT5*), with particular reference to the retinol metabolic pathway (*ALDH1A3*/*ADH1C*/*DHRS3*/*CYP26B1*/*CYP2B6*/*UGT1A8*). This is consistent with the *GSEA* results obtained with the *KEGG-Metabolism* platform (Fig. S5). The second-largest interactome (8 elements) is centered on *IRF1* (*Interferon-Responsive-Factor-1*) and it contains proteins involved in inflammation and antigen-presentation (*IRF1*/*SOCS1*/*SAMD9*/*TLR1*/*PSMB10*/*CTSS*). In this case too, up-regulation of the network is in line with the *GSEA* results (Fig. S4). The third-largest interactome (7 elements) contains proteins controlling ion-channels (*KCNE3*/*KCNN4*) and epithelial-polarization (*CYTH3*). The fourth-largest network includes the tumor-suppressor *VWA5A* [33] and 5 other proteins (*SAMD12*/*PLPP1*/*AGPAT3*/*CDS2*/*CPD*) regulating membrane phospholipids. Finally, one of the commonly up-regulated genes is *DMBT1*, a tumor-suppressive demethylase [34], which interacts with *PGR*.

As for the commonly down-regulated genes, the corresponding products aggregate into 14 interactomes (Fig. 6A, lower-right). The largest interactome (28 elements) contains factors involved in lipid metabolism (*MOGAT3*/*DGAT2*/*PCK1*/*NR1H3*/*SCARB1*/*PCSK9*/*PON2*/*CES2*/*CYP2C19*/*CYP4F12*/*CYP2C18*/*CYP2W1*/*SLC16A1*). Interestingly, other proteins controlling lipid metabolism (*SPTLC3*/*DEGS2*/*UGT8*/*GAL3ST1*) cluster inside a smaller network. This supports the idea that the anti-proliferative effects of ATRA in the *G-INT* neoplastic cells are associated with lipid-homeostasis modulation. The second-largest interactome of down-regulated gene-products (*CDH17*/*ITGA6*/*COL17A1*/*DSG3*/*TRIM29*/*SERPINB5*/*PLOD3*/*LAD1*/*P3H2*/*TMEM154*/*S100A14*/*S100A16*/*S100A4*) contains proteins involved in cell-adhesion and motility. Down-regulation of this last group of gene-products may relate to a potential increase in the process of epithelial-polarization mentioned in the case of the up-regulated *CYTH3* gene.

**Fig. 7:**
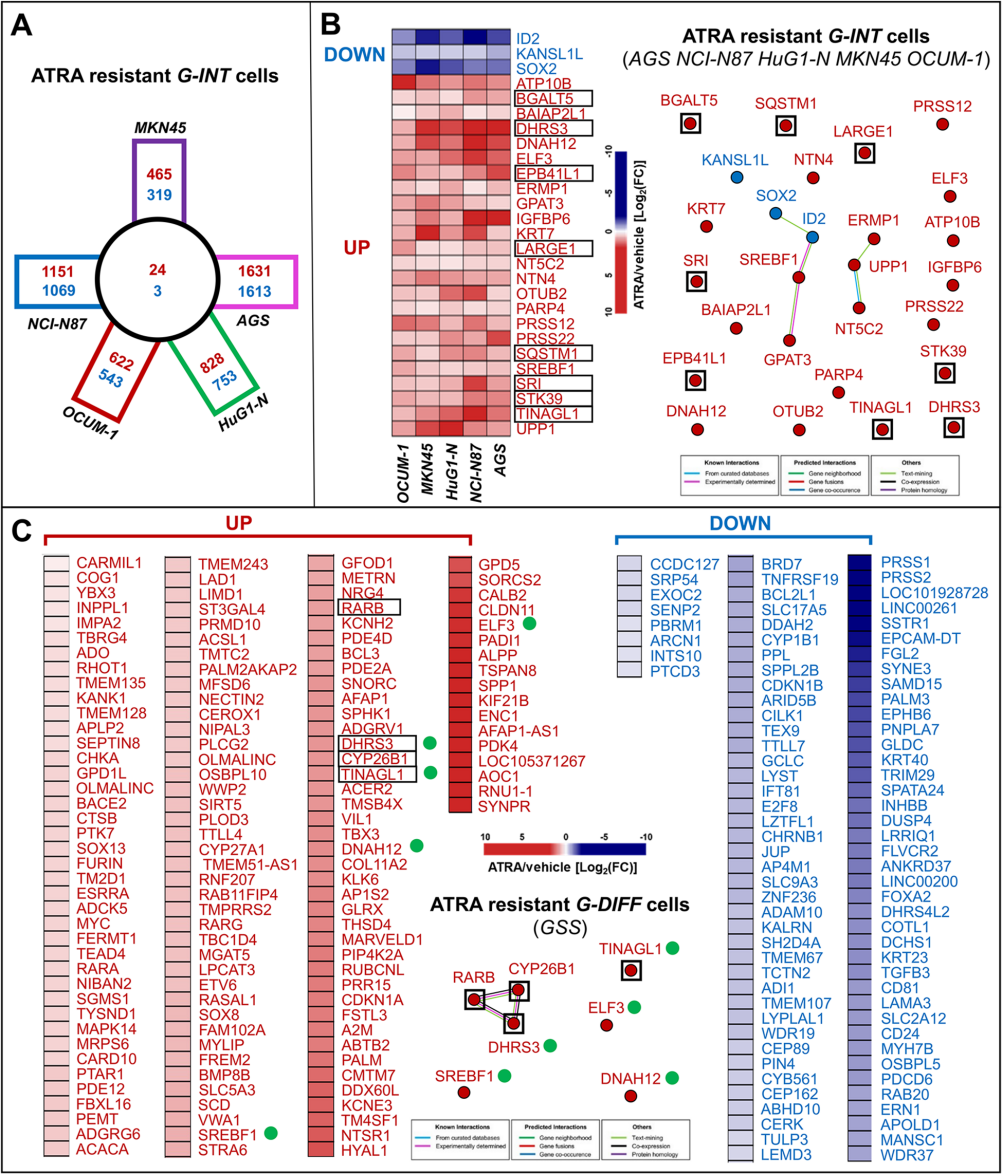
Gene-networks modulated by ATRA in retinoid-resistant *G-INT* and *G-DIFF* gastric cancer cell-lines. The *G-INT*/retinoid-resistant *MKN45*/*NCI-N87*/*AGS*/*OCUM-1*/*HuG1-N* cell-lines and the *G-DIFF*/retinoid-resistant *GSS* cell-line were exposed to vehicle (DMSO) or ATRA (1.0 µM) for 48 hours and subjected to *RNA-seq* analysis. **A** The panel illustrates the number of genes selectively up-regulated (red) or down-regulated (blue) in each *G-INT* cell-line (squares) and commonly up-regulated (red) or down-regulated (blue) in the 5 cell-lines (circle). **B** The left side of the panel shows a heat-map illustrating the effects of ATRA on the expression levels of the 27 genes commonly and significantly (*FDR* < 0.1) up-regulated (24 genes; UP) and down-regulated (3 genes; DOWN) in the 5 retinoid-resistant *G-INT* gastric-cancer cell-lines. The results are expressed as the ATRA-vehicle ratio [Log_2_ *FC* (*Fold-Change*)] and they represent the mean of 3 independent vehicle-treated and ATRA-treated cultures. The right side of the panel illustrates the results of a *STRING* (*Search-Tool-for-the-Retrieval-of-Interacting-Genes*/*Proteins*) analysis performed on the 24 up-regulated gene-products (red dots) and the 3 down-regulated gene products (blue dots). The proteins included in black squares are encoded by genes which are commonly up-regulated or down-regulated also in the 3 retinoid-sensitive *G-INT* cell-lines, *GSU*, *KATO-III* and *IM95*. **C** The panel shows two heat-maps illustrating the effects of ATRA on the expression levels of the 225 genes significantly (*FDR* < 0.1) up-regulated (137 genes; UP) and down-regulated (88 genes; DOWN) in the retinoid-resistant *G-DIFF* cell-line,* GSS*. In addition, the panel illustrates the results of a *STRING* analysis performed on the 7 gene products which are commonly up-regulated by ATRA in the *GSS* and the retinoid-sensitive *G-DIFF* cell-lines, *HGC-27*, *LMSU*, *GCIY* and *RERF-GC-1B* (black squares) or the retinoid-resistant *G-INT* cell-lines, *MKN45*, *NCI-N87*, *AGS*, *OCUM-1* and *HuG1-N* (green dots). The results are expressed as the ATRA-vehicle ratio [Log_2_ *FC* (*Fold-Change*)] and they represent the mean of 3 independent vehicle-treated and ATRA-treated cultures.

To evaluate possible associations with ATRA-sensitivity, we conducted similar studies in 5 *G-INT* cell-lines (*MKN45*, *AGS*, *NCI-N87*, *HuG1-N* and *OCUM-1*) characterized by low ATRA-sensitivity (*ATRA-score* < 0.55). The 5 cell-lines respond to ATRA with the up-/down-regulation of several hundred genes (Fig. 7A). Nevertheless, the number of commonly up-/down-regulated genes is limited to 24 and 3, respectively (Fig. 7A and B). Interestingly, 8 genes (*BGALT5*/*SRI*/*EPB41L1*/*TINAGL1*/*LARGE1*/*SQSTM1*/*STK39*/*DHRS3*), are up-regulated in both *G-INT* cells showing low and high ATRA-sensitivity (Figs. 6A and 7B, black-squares). A similar situation is observed in the case of the down-regulated *ID2* gene.

We extended our analyses to the *G-DIFF* context, performing studies in the *G-DIFF* cell-lines, *HGC-27*, *RERF-GC-1B*, *LMSU* and *GCIY* (*ATRA-score* > 0.55), showing high ATRA-sensitivity, as well as *GSS* cells, the only *G-DIFF* cell-line characterized by low ATRA-sensitivity (*ATRA-score* = 0.50). In *HGC-27* and *RERF-GC-1B* cells, ATRA modulates the expression of 6,063 and 5,557 genes, respectively, while a lower number of genes is up- and down-regulated in *LMSU* (1,886 genes) and *GCIY* (914 genes) cells (Fig. S6, right). In these retinoid-sensitive cell-lines, ATRA up- and down-regulates 33 and 10 common genes, respectively (Fig. 6B). The STRING analysis performed on the 33 up-regulated genes demonstrates that the corresponding proteins cluster into 3 networks. The largest network (13 elements) centers on *IRF1* and it contains factors regulating inflammation (*BIRC3*/*TLR3*/*USF1*/*CCL2*) and antigen-presentation (*PSME1*/*PSMB10*/*TAPBPL2*/*ERAP1*/*ERAP2*/*CTSS*). The second-largest network consists of 7 proteins (*CYP26B1*/*DHRS3*/*NRIP1*/*RARB*/*GPRC5A*/*RARRES3*/*APOL3*) controlling retinoid-metabolism and epithelial-differentiation. As for the 10 down-regulated genes, they do not code for any interacting protein. In retinoid-resistant *GSS* cells, ATRA causes a significant up- and down-regulation of 137 and 89 genes, respectively (Fig. 7C). Noticeably, 4 of these genes (*CYP26B1*/*RARB*/*DHRS3*/*TINAGL1*) are equally up-regulated in the retinoid-sensitive *G-DIFF* cell-lines, *GCIY*, *HGC-27*, *RERF-GC-1B* and *LMSU* (Fig. 7C, black-squares).

In a last set of studies, we looked for genes up- or down-regulated by ATRA in *G-INT* and *G-DIFF* cell-lines characterized by high ATRA-sensitivity (Fig. 6C). In all the *G-DIFF* and *G-INT* cell-lines ATRA increases the expression of 6 common genes, *i.e. IRF1*, *DHRS3*, *CTSS*, *PSMB10*, *CYP26B1* and *TINAGL1*. In contrast, only *AHNAK2* is down-regulated by ATRA in the entire set of *G-INT*/*G-DIFF* cell-lines. As already observed, the *CTSS* and *PSMB10* gene-products interact with *IRF1* and are part of the interactome centered on *IRF1* itself (Fig. 6A and B). Furthermore, the *CYP26B1* gene-product binds *DHRS3* and the 2 proteins are part of a network involved in retinoid metabolism and epithelial-differentiation (Fig. 6A and B).

### Role of *IRF1* and *DHRS3* in the growth inhibitory action of ATRA

Given the potential role played by *IRF1* in inflammatory/immunological/*IFN*-dependent responses [35, 36] and by *DHRS3* (*Short-Chain-Dehydrogenase/Reductase-Family-16 C-Member-1* or *Retinol-Dehydrogenase-17*) in retinoid metabolism, we conducted functional studies on the 2 factors in *HGC-27* cells.

**Fig. 8:**
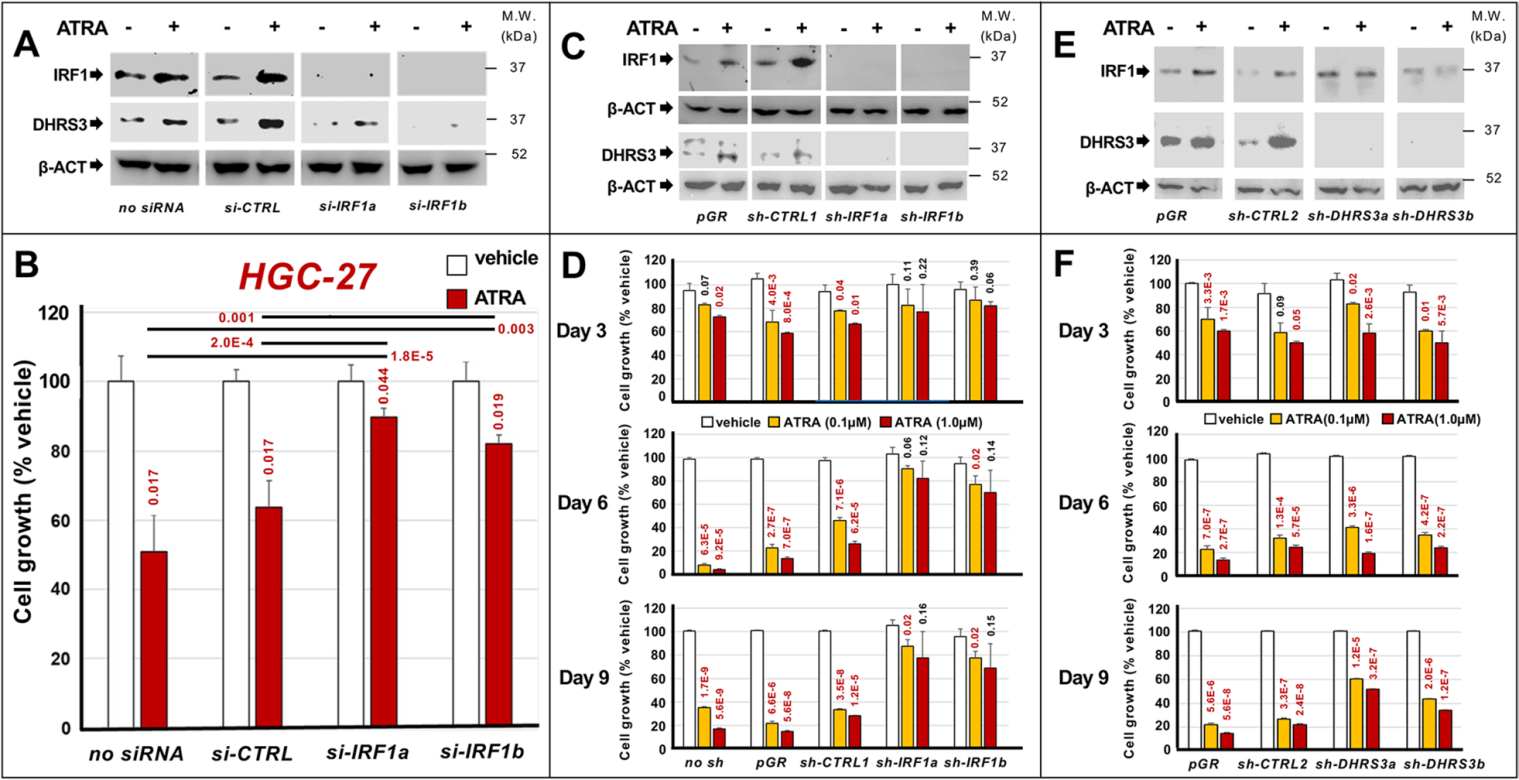
*IRF1* and *DHRS3* involvement in the anti-proliferative effects exerted by ATRA in *HGC-27* cells. *HGC-27* cells were transfected with two *IRF1-*targeting (*si-IRF1a*/*si-IRF1b*) and a control siRNA (*si-CTRL*). Twenty-four hours later, cells were treated with vehicle (DMSO) or ATRA (1µM) for 48 hours. **A** Western-blot analysis using anti-*IRF1*, anti-*DHRS3* and anti-*βactin* antibodies: the lanes marked as “no-siRNA” indicate *p*arental *HGC-27* cells. **B** Cell-growth of transfected *HGC-27* cells (MTS-assay): Mean+SD of 3 replicate cultures; values normalized for vehicle-treated cells (100%). The p-values (two-tailed Student's t-test) of the comparisons between ATRA-treated and vehicle-treated cells and the comparisons between the indicated groups are shown above each red column and above the diagram, respectively. **C** *HGC-27 *cells were infected with lentiviral particles containing 2 *IRF1-*targeting-shRNAs (*sh-IRF1a*/*sh-IRF1b*), one control-shRNA (*sh-CTRL1*) or the *pGreenPuro-*vector (*pGR*). Following puromycin-selection, we isolated 4 green-fluorescent cell-populations characterized by *pGR­*-*, sh-CTRL*-,* sh-IRF1a*- and *sh-IRF1b*­-integration. The cell-populations were treated with vehicle or ATRA (1µM) for 48 hours and subjected to Western-blot analysis using anti-*IRF1*, anti-*DHRS3* and anti-*βactin* antibodies as in (**A**). **D** The *pGR*-*, sh-CTRL*-,* sh-IRF1a­*- and *sh-IRF1b*-infected cell-populations were treated with vehicle or ATRA (0.1µM/1.0µM) for 3/6/9 days: “*no-sh*”=parental-*HGC-27* cells. Cell-growth (MTS-assay): each value is the Mean+SD of 3 cultures; values are normalized as in (**B**). The p-values (two-tailed-Student's-t-test) of the comparisons between ATRA-treated and corresponding vehicle-treated cells are shown above each column. **E** *HGC-27 *cells were infected with lentiviral-particles containing two *DHRS3*-targeting *pGreenPuro*-constructs (*sh-DHRS3a*/*sh-DHRS3b*), the *pGreenPuro*-vector (*pGR*) and a control shRNA (*sh-CTRL2*). Following infection/puromycin-selection, we isolated 4 populations of green-fluorescent *HGC-27* cells characterized by stable* pGR/sh-CTRL*/*sh-DHRS3a*/*sh-DHRS3b*-integration. The cell-populations were treated with vehicle or ATRA (1.0µM) for 48 hours and subjected to Western-blot analysis using anti-*IRF1*/anti-*DHRS3*/anti-*βactin* antibodies as in (**A**). **F** *pGR/sh-CTRL*/*sh-DHRS3a*/*sh-DHRS3b-*infected cell-populations were treated with vehicle or ATRA as in (**D**). Cell-growth (MTS-assay): Mean+SD of 3 cultures; values normalized as in (**D**). The *p*-values (two-tailed-Student’s-t-test) of the ATRA-treated/vehicle-treated cells comparison are shown above each column

In a first set of experiments, we transiently transfected *HGC-27* cells with 2 *IRF1*-targeting siRNAs (*siIRF1a* and *siIRF1b*) and a control siRNA (*siCTRL*). We exposed the transfected cells to vehicle or ATRA for 48 h. In accordance with the up-regulation of the corresponding mRNA, ATRA increases the basal levels of the *IRF1* protein in mock transfected (*no siRNA*) and *siCTRL* transfected *HGC-27* cells, as indicated by the Western-blot analyses performed (Fig. 8A). By converse, the *IRF1* protein is undetectable in *siIRF1a* and *siIRF1b* transfected cells regardless of vehicle or ATRA treatment. As ATRA co-regulates *IRF1* and *DHRS3* mRNAs, we determined the levels of the *DHRS3* protein as well. In native and *siCTRL*-transfected cells, ATRA induces the *DHRS3* protein (Fig. 8A). The two siRNAs, *siIRF1a* and *siIRF1b* reduce the basal levels of the *DHRS3* protein. More importantly, the retinoid-dependent *DHRS3* induction is abolished in *siIRF1a*- and *siIRF1b*-transfected cells. Thus, *IRF1* up-regulation may be responsible for the increase in *DHRS3* that ATRA triggers in gastric-cancer cells. As for the functional role played by *IRF1*, *siIRF1a* and *siIRF1b* reduce the anti-proliferative effects exerted by ATRA in native and *siCTRL*-transfected *HGC-27* cells (Fig. 8B). These data indicate that *IRF1* up-regulation takes part in the growth-inhibitory action exerted by ATRA in *HGC-27* cells. To confirm the role played by *IRF1* in ATRA-dependent growth inhibition of retinoid-sensitive gastric cancer cells, we performed similar silencing experiments in the *LMSU* cell line (Fig. S7). As expected, *siIRF1a*/*siIRF1b* transfection abolishes the constitutive levels of *IRF1* protein and suppresses the ATRA-dependent *IRF1* induction observed in mock transfected (*no siRNA*) and *siCTRL* transfected *LMSU* cells. In addition, *IRF1* silencing abrogates the growth inhibitory action exerted by ATRA in the cultures of the two control *LMSU* cell lines (Fig. S7). This is consistent with the results generated in the *HGC-27* line and it supports the idea that *IRF1* up-regulation is involved in the anti-proliferative action exerted by ATRA in retinoid-sensitive gastric cancer cells.

To validate the siRNA results we took a complementary approach based on the use of *IRF1*-targeting shRNAs. To this purpose, we infected *HGC-27* cells with lentiviral particles containing two distinct *IRF1*-targeting shRNAs (*shIRF1a* and *shIRF1b*), an untargeted control shRNA (*shCTRL1*) or the void vector (*pGR*). This resulted in the generation of 4 distinct and puromycin-resistant cell-populations characterized by stable integration of the *pGR, shCTRL1*, *shIRF1a* and *shIRF1b* constructs. Consistent with a silencing effect of the *shIRF1a* and *shIRF1b* constructs, the basal levels of the *IRF1* protein are measurable only in *pGR-* and *shCTRL1*-infected cells (Fig. 8C). As expected, ATRA causes a significant induction of the *IRF1* protein in both *pGR*- and *shCTRL1*-infected cells. This ATRA-dependent increase of the *IRF1* protein is abolished in *shIRF1a-* and *shIRF1b-*infected cells. As for *DHRS3*, vehicle-treated *pGR*- and *shCTRL1*-infected cells express measurable amounts of the corresponding protein. Upon exposure to ATRA, the two cell-populations show the expected induction of *DHRS3*. By converse, *shIRF1a*- and *shIRF1b*-infected cells do not express detectable levels of the *DHRS3* protein, in either basal conditions or following exposure to ATRA. To define the functional consequences of the *IRF1* knock-down, we exposed native as well as *pGR*-, *shCTRL1*-, *shIRF1a*- and *shIRF1b*-infected cells to vehicle and ATRA (0.1µM/1.0µM) for 3, 6 and 9 days. At day-6 and day-9, both concentrations of ATRA cause the expected reduction in the growth of native and *pGR*/*shCTRL1* infected cells, while the observed ATRA-dependent anti-proliferative effects are absent/reduced in *shIRF1a*- and *shIRF1b*-infected cells (Fig. 8D). Overall, the data obtained in these cellular models confirm the siRNA results.

In a second set of experiments, we studied the involvement of *DHRS3* in ATRA growth-inhibitory action, using the same shRNA approach described for *IRF1*. We infected *HGC-27* cells with lentiviral particles containing *DHRS3*-targeting shRNA constructs (*shDHRS3a* and *shDHRS3b*) and the *pGR*/*shCTRL2* controls. This resulted in the generation of distinct and puromycin-resistant cell-populations, which we treated with vehicle or ATRA for 48 h. Vehicle-treated *pGR* and *shCTRL2* cells synthesize significant amounts of *DHRS3*, whereas the protein is undetectable in *shDHRS3a* and *shDHRS3b* cells (Fig. 8E). As expected, ATRA increases the amounts of *DHRS3* in *pGR*- and *shCTRL2*-infected cells, while the protein remains undetectable in retinoid-treated *shDHRS3a*- and *shDHRS3b*-infected cells. Surprisingly, *DHRS3*-silencing blocks the ATRA-dependent induction of *IRF1*, which suggests a positive feed-back loop. To define the consequences of *DHRS3*-silencing, we treated *pGR*- *shCTRL2*-, *shDHRS3a*- and *shDHRS3b*-infected cells with vehicle or ATRA (0.1µM/1.0µM) for 3, 6 and 9 days. At all these time-points, both concentrations of ATRA reduce the growth of *pGR*-/*shCTRL*-infected cells (Fig. 8F). At day-9, the ATRA-dependent anti-proliferative effect observed in *pGR*-/*shCTRL*-infected cells is diminished in the *shDHRS3a*-/*shDHRS3b*-infected counterparts. These last data indicate that also *DHRS3* up-regulation may be involved, at least partially, in the growth-inhibitory effects exerted by ATRA in gastric-cancer cells. However, it must be pointed out that the RNA-sequencing data indicate that ATRA induces *DHRS3* expression in all the gastric cancer cell-lines considered, regardless of their relative sensitivity/resistance to the retinoid. This suggests that *DHRS3* is likely to require other gene-products/factors or pathways, which are selectively stimulated/repressed by the retinoid only in sensitive gastric cancer cells, to play a role in the anti-proliferative action of ATRA.

In a last set of studies, we evaluated whether ATRA-dependent up-regulation of the *IRF1* protein is dependent or independent of retinoid sensitivity. To address the point we determined the amounts of the protein in four representative lines characterized by a high level of resistance to the anti-proliferative effects of ATRA, *i.e. MKN74* (*G-DIFF*), *AGS* (*G-INT*), *HuG1-N* (*G-INT*) and *OCUM-1* (*G-INT*) cells. As in the case of the retinoid-sensitive *HGC-27* and *LMSU* cells, we exposed the four cell-lines to ATRA (1µM) for 48 h and we determined the levels of the *IRF1* protein by Western blot analysis (Fig. S8A). In basal conditions, *MKN74* cells express higher levels of the *IRF1* protein than the *AGS*, *HuG1-N* and *OCUM-1* counterparts. ATRA exerts no effect on the amounts of the *IRF1* protein determined in the 4 retinoid resistant cell-lines. This supports the idea that ATRA-dependent up-regulation of the *IRF1* protein is a characteristic of the retinoid sensitive gastric cancer cell-lines. The results described above prompted us to evaluate the effects of *IRF1* over-expression on the growth of one cell-line characterized by low sensitivity to the anti-proliferative effects of ATRA. We conducted these studies in the retinoid-resistant *AGS* cell-line, by stable transfection of a plasmid construct allowing expression of a functionally active *IRF1* protein (Fig. S8B). In our experimental conditions, *IRF1* over-expression does not alter the growth of *AGS* cells exposed to either vehicle or ATRA (Fig. S8C).

Overall, the data obtained with the knock-down and over-expression approaches considered suggest that *IRF1* up-regulation is a necessary but insufficient determinant of the anti-proliferative action exerted by ATRA in gastric cancer cells.

## Discussion

The results obtained in this pre-clinical study indicate that ATRA has significant potential in the treatment of patients affected by gastric-cancer. Indeed, our experimental data and predictions support the idea that at least one-half of the gastric-cancer cases may benefit from ATRA-based therapeutic strategies. Interestingly, ATRA-sensitive tumors are not enriched for any of the gastric-cancer sub-types defined according to the *Lauren*’s classification, histological characteristics or gene-expression profiles. The platform of 42 genes whose basal expression is directly or inversely associated to ATRA-sensitivity provides indications as to the characteristics of the gastric-cancers which respond to the retinoid. Among the genes directly associated with ATRA-sensitivity, *WNT2* stands out. In fact, high expression of *WNT2* is a negative prognostic factor, which associates with the invasive/metastatic behavior of gastric-cancer cells [37, 38], *via* stimulation of the *EMT* (*Epithelial-to-Mesenchimal-Transition*) process [39]. Thus, *WNT2* and *EMT* activation may be factors involved in the sensitivity of gastric-cancer cells to ATRA. With respect to this, the direct correlation of *LOXL1* (*Lysyl-Oxidase-Like-1*) levels with ATRA-sensitivity is consistent with the idea that *EMT* represents a determinant of this sensitivity. Indeed, the enzyme is over-expressed in gastric-cancer cells and it favors their *EMT*-dependent dissemination in the peritoneum [40].

Another important result of our studies is the observation that ATRA stimulates *IFN*-dependent immune-responses and increases antigen presentation in gastric-cancer cell-lines. This is similar to what we observed in the context of breast-cancer [13]. In breast cancer, the immune-modulatory effects activated by ATRA are associated with a “*viral-mimicry*” response resulting from an increase in the RNAs produced by endogenous retroviruses. In contrast, the ATRA-dependent immune-modulation observed in gastric-cancer cells is not accompanied by a “*viral-mimicry*” response, as we observe no alteration in the expression of the numerous endogenous retroviral mRNAs detected (Table S5). The data suggest that the molecular mechanisms underlying the immune-modulatory action of ATRA in gastric- and breast-cancers are different. From a translational and therapeutic prospective, the immune responses activated by ATRA in gastric-cancer are of great relevance, as they indicate that the retinoid is likely to increase the sensitivity of this tumor to immune-modulatory agents, such as immune-checkpoint inhibitors [41, 42].

A final and significant outcome of our study is the identification of a restricted number of genes which are up-regulated (*IRF1*, *CTSS*, *PSMB10*, *CYP26B1*, *DHRS3* and *TINAGL1*) and down-regulated (*AHNAK2*) by ATRA in all the retinoid-sensitive gastric-cancer cell-lines considered. Interestingly, *IRF1*, *CTSS* and *PSMB10* are direct or indirect transcriptional targets of ATRA [43–45] and code for interacting proteins involved in the regulation of immunity [35, 46, 47]. In particular, the *IRF1* gene is likely to play a key role in the *IFN*-dependent and immunological responses activated by ATRA in gastric-cancer. Moreover, *CYP26B1* and *DHRS3* up-regulation indicates that ATRA causes significant effects on the metabolism of endogenous vitamin-A in gastric-cancer cells. In fact, *CYP26B1* codes for a protein catalyzing the oxidation of ATRA into an inactive oxo-derivative [48], while *DHRS3* codes for an NADPH-dependent enzyme which reduces retinaldehyde into retinol [49] [50] [51]. From a functional point of view, *TINAGL1* is one of the most interesting up-regulated genes, since the corresponding protein-product suppresses the progression and diffusion of triple-negative breast-cancer *via* inhibition of the *FAK* and *EGFR* signaling pathways [52]. Finally, *AHNAK2* is the only gene, which is down-regulated by ATRA in sensitive gastric-cancer cells. *AHNAK2* codes for an oncogenic nucleoprotein [53], which may play a role in calcium signaling [54]. In gastric-cancer, *AHNAK2* expression is involved in the resistance to chemotherapeutics [55]. Thus, *AHNAK2* down-regulation suggests that ATRA may boost the efficacy of certain chemotherapeutics in the clinics. Although functional studies need to be performed for all the above genes, we focused our initial attention on *IRF1* and *DHRS3*. Our results indicate that *IRF1* and to a lesser extent *DHRS3* modulate the growth-inhibitory action of ATRA in retinoid-sensitive gastric-cancer cells. In fact, knock-down of the two corresponding proteins suppresses and reduces ATRA anti-proliferative action in representative cell-lines. The modulatory effect exerted by *IRF1* in gastric cancer cells seems to be limited to ATRA-sensitive cell-lines, as indicated by the results obtained in *G-INT* and ATRA-resistant *AGS* cells which were engineered to over-express *IRF1* (Fig. S8).

Overall, the data obtained in this study suggest that pharmacological agents targeting the two proteins may be used for the design of innovative therapeutic strategies in retinoid-sensitive gastric-cancers.

## Conclusion

The present study provides data supporting the therapeutic potential of ATRA in the stratified/personalized treatment of gastric-cancer. In this context, the gene-expression model that we generated will permit the development of a predictive clinical tool for the selection of patients who may benefit from ATRA-based therapeutic strategies. Overall, our data represent the fundaments for the design/organization of clinical trials focusing on the use of ATRA in the treatment of this heterogeneous type of tumor. In particular, the strong immune-regulatory responses activated by the retinoid suggest that ATRA and immune checkpoint inhibitors constitute rational combinations in the management of gastric-cancer.

### Supplementary Information

**Additional file 1:** **Supplementary Methods. Supplementary Table S1. **Characteristics and source of the gastric-cancer cell-lines. **Supplementary Table S2. **Structure of the double stranded DNAs used for the construction of the shRNA plasmid constructs. **Figure S1.** Growth curves of the gastric cancer cell-lines exposed to increasing concentrations of ATRA. **Figure S2.** Ki67 immune-histochemistry in tissue-slice cultures of representative primary gastric-cancers exposed to ATRA. **Figure S3.** Effects of ATRA on the body weight of SCID mice transplanted with LMSU and NCI-N87 cells. **Figure S4. **HALLMARK pathway analysis of the RNA-seq results obtained following treatment of the indicated gastric cell lines with ATRA. **Figure S5.** KEGG pathway analysis of the RNA-seq results obtained following treatment of the indicated gastric cell lines with ATRA. **Figure S6.** Number of genes modulated by ATRA in retinoid-sensitive G-INT and G-DIFF gastric cancer cell-lines.**Figure S7.** Effects of ATRA on IRF1 protein levels and cell-growth in the retinoid-sensitive LMSU cell-line.**Figure S8.** Effects of ATRA on IRF1 protein expression in retinoid resistant gastric cancer cells and IRF1 over-expression in AGS cells. Original Western blots.

**Additional file 2:** **Table S3. **Characteristics of the gastric-cancer patients used for the studies involving tissue-slice cultures*. *The table summarizes the clinical characteristics of the 13 patients considered.

**Additional file 3:** **Table S4. **RNA-sequencing data. The table contains the processed *RNA-seq* data obtained with our panel of gastric cancer cell-lines exposed to vehicle and ATRA.

**Additional file 4:** **Table S5. **Effects of ATRA on the expression of the RNAs derived from endogenous retroviruses. The table contains the levels of endogenous retroviral RNAs determined by *RNA-seq* data obtained from the indicated cell lines exposed to vehicle and ATRA.

## Data Availability

All the cell-lines used in the study are available upon request. The datasets used and/or analysed during the current study are available from the corresponding author on reasonable request. All data generated or analysed during this study are included in this published article and its supplementary information files. The raw data of the RNA-sequencing studies are also available in the *EMBL-EBI Annotare* database (https://www.ebi.ac.uk/fg/annotare/) under the accession numbers: E-MTAB-12,387 (Cell-lines), E-MTAB-12,385 (Patients). The processed *RNA-seq* data of our gastric-cancer cell-lines are available in Table S4.

## Abbreviations

ATRA: All-Trans Retinoic-acid
APL: Acute-Promyelocytic-Leukemia
RNA-seq: RNA-sequencing
AUC: Area Under the Curve
GSEA: Gene Set Enrichment Analysis
CCLE: Cancer Cell Line Encyclopedia
TCGA: The Cancer Genome Atlas
GSVA: Gene Set Variation Analysis

## References

[1] Hartgrink HH, Jansen EP, van Grieken NC, van de Velde CJ (2009). Gastric cancer. Lancet.

[2] Kamangar F, Dores GM, Anderson WF (2006). Patterns of Cancer Incidence, Mortality, and Prevalence Across Five Continents: defining priorities to reduce Cancer disparities in different Geographic regions of the World. JCO..

[3] Laurén P (1965). The two histological main types of gastric carcinoma: diffuse and so-called intestinal-type carcinoma: an attempt at a histo-clinical classification. Acta Pathol Microbiol Scand.

[4] Lim HJ, Zhuang L, Fitzgerald RC (2023). Current advances in understanding the molecular profile of hereditary diffuse gastric cancer and its clinical implications. J Exp Clin Cancer Res.

[5] Hu B, El Hajj N, Sittler S, Lammert N, Barnes R, Meloni-Ehrig A (2012). Gastric cancer: classification, histology and application of molecular pathology. J Gastrointest Oncol.

[6] Yeoh KG, Tan P (2022). Mapping the genomic diaspora of gastric cancer. Nat Rev Cancer.

[7] Wang Q, Liu G, Hu C (2019). Molecular classification of gastric adenocarcinoma. Gastroenterol Res.

[8] Tan IB, Ivanova T, Lim KH, Ong CW, Deng N, Lee J (2011). Intrinsic subtypes of gastric cancer, based on gene expression pattern, predict survival and respond differently to chemotherapy. Gastroenterology.

[9] Xu W, Yang Z, Lu N (2016). Molecular targeted therapy for the treatment of gastric cancer. J Exp Clin Cancer Res.

[10] Wang ZY, Chen Z (2000). Differentiation and apoptosis induction therapy in acute promyelocytic Leukaemia. Lancet Oncol.

[11] Sanz MA, Fenaux P, Tallman MS, Estey EH, Löwenberg B, Naoe T (2019). Management of acute promyelocytic leukemia: updated recommendations from an expert panel of the European LeukemiaNet. Blood.

[12] Cicconi L, Fenaux P, Kantarjian H, Tallman M, Sanz MA, Lo-Coco F (2018). Molecular remission as a therapeutic objective in acute promyelocytic leukemia. Leukemia.

[13] Bolis M, Paroni G, Fratelli M, Vallerga A, Guarrera L, Zanetti A (2020). All-trans retinoic acid stimulates viral mimicry, Interferon responses and antigen presentation in breast-cancer cells. Cancers.

[14] Bolis M, Garattini E, Paroni G, Zanetti A, Kurosaki M, Castrignanò T (2017). Network-guided modeling allows tumor-type independent prediction of sensitivity to all-trans-retinoic acid. Ann Oncol.

[15] Terao M, Goracci L, Celestini V, Kurosaki M, Bolis M, Di Veroli A (2019). Role of mitochondria and cardiolipins in growth inhibition of Breast cancer cells by retinoic acid. J Exp Clin Cancer Res.

[16] Centritto F, Paroni G, Bolis M, Garattini SK, Kurosaki M, Barzago MM (2015). Cellular and molecular determinants of all-trans retinoic acid sensitivity in breast cancer: luminal phenotype and RARα expression. EMBO Mol Med.

[17] Dobin A, Davis CA, Schlesinger F, Drenkow J, Zaleski C, Jha S (2013). STAR: ultrafast universal RNA-seq aligner. Bioinformatics.

[18] Love MI, Huber W, Anders S (2014). Moderated estimation of fold change and dispersion for RNA-seq data with DESeq2. Genome Biol.

[19] Liberzon A, Subramanian A, Pinchback R, Thorvaldsdóttir H, Tamayo P, Mesirov JP (2011). Molecular signatures database (MSigDB) 3.0. Bioinformatics.

[20] Athar A, Füllgrabe A, George N, Iqbal H, Huerta L, Ali A (2019). ArrayExpress update – from bulk to single-cell expression data. Nucleic Acids Res.

[21] Murtagh F, Legendre P (2014). Ward’s hierarchical agglomerative clustering method: which Algorithms Implement Ward’s Criterion?. J Classif.

[22] Shannon P, Markiel A, Ozier O, Baliga NS, Wang JT, Ramage D (2003). Cytoscape: a software environment for integrated models of biomolecular interaction networks. Genome Res.

[23] Szklarczyk D, Gable AL, Nastou KC, Lyon D, Kirsch R, Pyysalo S (2021). The STRING database in 2021: customizable protein–protein networks, and functional characterization of user-uploaded gene/measurement sets. Nucleic Acids Res.

[24] Subramanian A, Tamayo P, Mootha VK, Mukherjee S, Ebert BL, Gillette MA (2005). Gene set enrichment analysis: a knowledge-based approach for interpreting genome-wide expression profiles. Proc Natl Acad Sci USA.

[25] Hänzelmann S, Castelo R, Guinney J (2013). GSVA: gene set variation analysis for microarray and RNA-Seq data. BMC Bioinformatics.

[26] Hoshida Y (2010). Nearest template prediction: a single-sample-based flexible class prediction with confidence assessment. PLoS One.

[27] Berlth F (2014). Pathohistological classification systems in gastric cancer: diagnostic relevance and prognostic value. WJG.

[28] van der Kuip H, Mürdter TE, Sonnenberg M, McClellan M, Gutzeit S, Gerteis A (2006). Short term culture of Breast cancer tissues to study the activity of the anticancer drug taxol in an intact tumor environment. BMC Cancer.

[29] Garattini E, Bolis M, Garattini SK, Fratelli M, Centritto F, Paroni G (2014). Retinoids and Breast cancer: from basic studies to the clinic and back again. Cancer Treat Rev.

[30] Petkovich M, Chambon P (2022). Retinoic acid receptors at 35 years. J Mol Endocrinol.

[31] Delescluse C, Cavey MT, Martin B, Bernard BA, Reichert U, Maignan J (1991). Selective high affinity retinoic acid receptor alpha or beta-gamma ligands. Mol Pharmacol.

[32] Sun SY, Yue P, Mao L, Dawson MI, Shroot B, Lamph WW (2000). Identification of receptor-selective retinoids that are potent inhibitors of the growth of human head and neck squamous cell carcinoma cells. Clin Cancer Res.

[33] Zhou YQ, Chen SL, Ju JY, Shen L, Liu Y, Zhen S (2009). Tumor suppressor function of *BCSC-1* in nasopharyngeal carcinoma. Cancer Sci.

[34] Kang W, Nielsen O, Fenger C, Leslie G, Holmskov U, Reid KBM (2005). Induction of DMBT1 expression by reduced ERK activity during a gastric mucosa differentiation-like process and its association with human gastric cancer. Carcinogenesis.

[35] Zhou H, Tang YD, Zheng C (2022). Revisiting IRF1-mediated antiviral innate immunity. Cytokine Growth Factor Rev.

[36] Feng H, Zhang YB, Gui JF, Lemon SM, Yamane D (2021). Interferon regulatory factor 1 (IRF1) and anti-pathogen innate immune responses. Blumenthal A, curatore. PLoS Pathog.

[37] Lin L, Li L, Ma G, Kang Y, Wang X, He J (2022). Overexpression of IL-8 and Wnt2 is associated with prognosis of gastric cancer. Folia Histochem Cytobiol.

[38] Zhang Z, Wang J, Dong X (2018). Wnt2 contributes to the progression of gastric cancer by promoting cell migration and invasion. Oncol Lett.

[39] Katoh M (2005). Epithelial-mesenchymal transition in gastric cancer (review). Int J Oncol.

[40] Hu Q, Masuda T, Kuramitsu S, Tobo T, Sato K, Kidogami S (2020). Potential association of LOXL1 with peritoneal dissemination in gastric cancer possibly via promotion of EMT. PLoS One.

[41] Yoon J, Kim TY, Oh DY (2023). Recent progress in Immunotherapy for gastric cancer. J Gastric Cancer.

[42] Shen J, Wang Z (2022). Recent advances in the progress of immune checkpoint inhibitors in the treatment of advanced gastric cancer: a review. Front Oncol.

[43] Yang XW, Wang P, Liu JQ, Zhang H, Xi WD, Jia XH (2014). Coordinated regulation of the immunoproteasome subunits by PML/RARα and PU.1 in acute promyelocytic Leukemia. Oncogene.

[44] Shen M, Bunaciu RP, Congleton J, Jensen HA, Sayam LG, Varner JD (2011). Interferon regulatory factor-1 binds c-Cbl, enhances mitogen activated protein kinase signaling and promotes retinoic acid-induced differentiation of HL-60 human myelo-monoblastic Leukemia cells. Leuk Lymphoma.

[45] Coyle KM, Maxwell S, Thomas ML, Marcato P (2017). Profiling of the transcriptional response to all-trans retinoic acid in Breast cancer cells reveals RARE-independent mechanisms of gene expression. Sci Rep.

[46] Wang Y, Yan K, Guo Y, Lu Y, Su H, Li H (2022). IP-score correlated to endogenous tumour antigen peptide processing: a candidate clinical response score algorithm of immune checkpoint inhibitors therapy in multiple cohorts. Front Immunol.

[47] de Mingo Pulido Á, de Gregorio E, Chandra S, Colell A, Morales A, Kronenberg M (2018). Differential Role of Cathepsins S and B in hepatic APC-Mediated NKT Cell activation and cytokine secretion. Front Immunol.

[48] Isoherranen N, Zhong G (2019). Biochemical and physiological importance of the CYP26 retinoic acid hydroxylases. Pharmacol Ther.

[49] Gudas LJ (2022). Retinoid metabolism: new insights. J Mol Endocrinol.

[50] Billings SE, Pierzchalski K, Tjaden NEB, Pang X, Trainor PA, Kane MA (2013). The retinaldehyde reductase DHRS3 is essential for preventing the formation of excess retinoic acid during embryonic development. FASEB J.

[51] Adams MK, Belyaeva OV, Wu L, Kedishvili NY (2014). The retinaldehyde reductase activity of DHRS3 is reciprocally activated by Retinol dehydrogenase 10 to Control Retinoid Homeostasis. J Biol Chem.

[52] Shen M, Jiang YZ, Wei Y, Ell B, Sheng X, Esposito M (2019). Tinagl1 suppresses triple-negative Breast Cancer Progression and Metastasis by simultaneously inhibiting Integrin/FAK and EGFR Signaling. Cancer Cell.

[53] Xu M, Cheng A, Yu L, Wei W, Li J, Cai C (2022). AHNAK2 is a biomarker and a potential therapeutic target of adenocarcinomas. Acta Biochim Biophys Sin (Shanghai).

[54] Komuro A, Masuda Y, Kobayashi K, Babbitt R, Gunel M, Flavell RA (2004). The AHNAKs are a class of giant propeller-like proteins that associate with calcium channel proteins of cardiomyocytes and other cells. Proc Natl Acad Sci U S A.

[55] Ohmura H, Ito M, Uchino K, Okada C, Tanishima S, Yamada Y (2020). Methylation of drug resistance-related genes in chemotherapy-sensitive Epstein-Barr virus-associated gastric cancer. FEBS Open Bio.

