# Supplementary material for: Anti-tumor activity of all-trans retinoic acid in gastric-cancer: gene-networks and molecular mechanisms
Source: J Exp Clin Cancer Res. 2023 Nov 11;42:298. doi: 10.1186/s13046-023-02869-w (PMC10638833; doi:10.1186/s13046-023-02869-w)
Supplement: Supplementary file 1 — Additional file 1: Supplementary Methods. Supplementary Table S1. Characteristics and source of the gastric-cancer cell-lines. Supplementary Table S2. Structure of the double stranded DNAs used for the construction of the shRNA plasmid constructs. Figure S1. Growth curves of the gastric cancer cell-lines exposed to increasing concentrations of ATRA. Figure S2. Ki67 immune-histochemistry in tissue-slice cultures of representative primary gastric-cancers exposed to ATRA. Figure S3. Effects of ATRA on the body weight of SCID mice transplanted with LMSU and NCI-N87 cells. Figure S4. HALLMARK pathway analysis of the RNA-seq results obtained following treatment of the indicated gastric cell lines with ATRA. Figure S5. KEGG pathway analysis of the RNA-seq results obtained following treatment of the indicated gastric cell lines with ATRA. Figure S6. Number of genes modulated by ATRA in retinoid-sensitive G-INT and G-DIFF gastric cancer cell-lines.Figure S7. Effects of ATRA on IRF1 protein levels and cell-growth in the retinoid-sensitive LMSU cell-line.Figure S8. Effects of ATRA on IRF1 protein expression in retinoid resistant gastric cancer cells and IRF1 over-expression in AGS cells. Original Western blots. [file 13046_2023_2869_MOESM1_ESM.pdf]

SUPPLEMENTARY INFORMATION

Title: Anti-tumor activity of All-Trans Retinoic Acid in gastric-cancer: gene-networks and molecular mechanisms

Guarrera Luca, Kurosaki Mami, Garattini Silvio Ken, Gianni' Maurizio, Fasola Gianpiero, Rossit Luca, Prisciandaro Michele, Di Bartolomeo Maria, Bolis Marco, Rizzo Paola, Nastasi Claudia, Foglia Marika, Zanetti Adriana, Paroni Gabriela, Terao Mineko and Garattini Enrico

CONTENT

Supplementary Methods page 2
Legends to Tables S3-S5 page 3
Supplementary Table S1 page 4
Supplementary Table S2 page 5
Figure S1 page 6
Figure S2 page 7
Figure S3 page 8
Figure S4 page 9
Figure S5 page 10
Figure S6 page 11
Figure S7 page 12
Figure S8 page 13
Original Western blots page 14-28

SUPPLEMENTARY METHODS

Short-term tissue slice cultures

The short term tissue slice cultures were prepared and used as already described (Centritto F, Paroni G, Bolis M, Garattini SK, Kurosaki M, Barzago MM, et al. Cellular and molecular determinants of all-trans retinoic acid sensitivity in breast cancer: Luminal phenotype and RARα expression. EMBO Mol Med. 2015;7:950–72). Briefly, tissue slices (thickness, 200 μm) deriving from surgical specimens of 13 gastric cancer patients who underwent a diagnostic Tru-cut procedure were obtained within 24 hours from the resection. Tissue slices were challenged with vehicle (DMSO) or ATRA (1.0 μM) for 48 hours in a 1:1 mixture of DMEM/F12 medium containing EGF (20 ng/ml), FGF (20ng/ml), insulin (5μg/ml), glucose (0.3%) in the presence *Antibiotic-Antimycotic* solution (GIBCO). At the end of the treatment, samples were fixed, paraffin-included, and dissected into 5-μm slices, which were subjected to immuno-histochemical staining with an antibody targeting the Ki67 proliferation-associated marker. The percentage of Ki67-positive tumor cells in the various samples was assessed in a quantitative manner by automatic image analysis. Scoring of Ki67 was blinded as to treatment. Each value represents the mean ± SE of at least five separate fields for each experimental sample.

Summary of the predicted ATRA-scores and histological characteristics of the gastric-cancer patients available in the TCGA database

Total No. of Diffuse Type Stomach Adenocarcinoma = 63; cases with calculated *ATRA-score* ≥0.55 = 35 (56%); with calculated *ATRA-score* <0.55 = 28 (44%)

Total No. of Mucinous Stomach Adenocarcinoma = 19; cases with calculated *ATRA-score* ≥0.55 = 10 (53%); with calculated *ATRA-score* <0.55 = 9 (47%)

Total No. of Papillary Stomach Adenocarcinoma = 5; cases with calculated *ATRA-score* ≥0.55 = 0 (0%); with calculated *ATRA-score* <0.55 = 5 (100%)

Total No. of Signet Ring Cell Carcinoma of the Stomach = 12; cases with calculated *ATRA-score* ≥0.55 = 6 (50%); with calculated *ATRA-score* <0.55 = 6 (50%)

Total No. of Stomach Adenocarcinoma = 207; cases with calculated *ATRA-score* ≥0.55 = 87 (42%); with calculated *ATRA-score* <0.55 = 120 (58%)

Total No. of Tubular Stomach Adenocarcinoma = 69; cases with calculated *ATRA-score* ≥0.55 = 22 (32%); with calculated *ATRA-score* <0.55 = 47 (68%)

LEGENDS TO SUPPLEMENTARY TABLES S3-S5

Table S3 *Characteristics of the gastric-cancer patients used for the studies involving tissue-slice cultures*
The table summarizes the clinical characteristics of the 13 patients considered.

Table S4 *RNA-sequencing data*
The table contains the processed *RNA-seq* data obtained with our panel of gastric cancer cell-lines exposed to vehicle and ATRA.

Table S5 *Effects of ATRA on the expression of the RNAs derived from endogenous retroviruses*
The table contains the levels of endogenous retroviral RNAs determined by *RNA-seq* data obtained from the indicated cell lines exposed to vehicle and ATRA.

Cell line	Group	Cellosaurus Expasy	Source	Cluster
231132/87	Gastric adenocarcinoma	CVCL_1046	DSMZ ACC-201	*G-INT*
AGS	Gastric adenocarcinoma	CVCL_0139	ATCC CRL-1739	*G-INT*
GSU	Gastric carcinoma	CVCL_8877	RIKEN RCB2278	*G-INT*
HuG1-N	Gastric tubular adenocarcinoma	CVCL_4846	RIKEN RCB1178	*G-INT*
IM95	Gastric adenocarcinoma	CVCL_2961	□JCRB1075.0	*G-INT*
KATO-III	Signet ring cell gastric adenocarcinoma	CVCL_0371	ATCC HTB-103	*G-INT*
KE-39	Stomach adenocarcinoma	CVCL_3385	RIKEN RCB1434	*G-INT*
MKN45	Gastric adenocarcinoma	CVCL_0434	DSMZ ACC-409	*G-INT*
NCI-N87	Gastric tubular adenocarcinoma	CVCL_1603	ATCC CRL-5822	*G-INT*
NUGC-4	Signet ring cell gastric adenocarcinoma	CVCL_3082	□JCRB0834	*G-INT*
OCUM-1	Signet ring cell gastric adenocarcinoma	CVCL_3084	□JCRB0192	C1
SNU-16	Gastric adenocarcinoma	CVCL_0076	ATCC CRL-5974	*G-INT*
SNU-5	Gastric adenocarcinoma	CVCL_0078	ATCC CRL-5973	*G-INT*
ECC10	Gastric small cell neuroendocrine carcinoma	CVCL_1188	RIKEN RCB0983	*G-DIFF*
ECC12	Gastric small cell neuroendocrine carcinoma	CVCL_1189	RIKEN RCB1009	*G-DIFF*
GCIY	Gastric adenocarcinoma	CVCL_1228	RIKEN RCB0555	*G-DIFF*
GSS	Gastric carcinoma	CVCL_8876	RIKEN RCB2277	*G-DIFF*
HGC-27	Gastric carcinoma	CVCL_1279	ECACC 94042256	*G-DIFF*
Hs746.T	Gastric adenocarcinoma	CVCL_0333	ATCC HTB-135	*G-DIFF*
LMSU	Signet ring cell gastric adenocarcinoma	CVCL_5081	RIKEN RCB1062	*G-DIFF*
MKN1	Gastric adenosquamous carcinoma	CVCL_1415	JCRB0252	*G-DIFF*
MKN7	Gastric tubular adenocarcinoma	CVCL_1417	JCRB1025	*G-DIFF*
MKN74	Gastric tubular adenocarcinoma	CVCL_2791	JCRB0255	*G-DIFF*
NUGC-3	Gastric adenocarcinoma	CVCL_1612	JCRB0822	*G-DIFF*
RERF-GC1-B	Gastric carcinoma	CVCL_3152	JCRB1009	*G-DIFF*
SH-10-TC	Mucinous gastric adenocarcinoma	CVCL_5167	RIKEN RCB1940	*G-DIFF*
SNU-1	Gastric adenocarcinoma	CVCL_0099	ATCC CRL-5971	*G-DIFF*

Table S1 *Characteristics and source of the gastric-cancer cell-lines*
The cell-lines characterized by a *G-INT* and a *G-DIFF* phenotype are marked in blue and red, respectively. All the cell-lines used throughout the study were free from mycoplasma contamination.

***sh-IRF1a* sense**	**GATCCGATACAAAGCAGGGGAAAACTTCCTGTCAGATTTTCCCCTGCTTTGTATCTTTTTG**
***sh-IRF1a* antisense**	**AATTCAAAAAGATACAAAGCAGGGGAAAATCTGACAGGAAGTTTTCCCCTGCTTTGTATCG**
***sh-IRF1b* sense**	**GATCCCCCTGATACCTTCTCTGATCTTCCTGTCAGAATCAGAGAAGGTATCAGGGTTTTTG**
***sh-IRF1b* antisense**	**AATTCAAAAACCCTGATACCTTCTCTGATTCTGACAGGAAGATCAGAGAAGGTATCAGGGG**
***sh-CTRL1* sense**	**GATCCGAACTCAGGATCTTTGGTACTTCCTGTCAGATACCAAAGATCCTGAGTTCTTTTTG**
***sh-CTRL1* antisense**	**AATTCAAAAAGAACTCAGGATCTTTGGTATCTGACAGGAAGTACCAAAGATCCTGAGTTCG**
***sh-DHRS3a* sense**	**GATCCCCTAATGGACAGTGATGATCTTCCTGTCAGAATCATCACTGTCCATTAGGTTTTTG**
***sh-DHRS3a* antisense**	**AATTCAAAAACCTAATGGACAGTGATGATTCTGACAGGAAGATCATCACTGTCCATTAGGG**
***sh-DHRS3b* sense**	**GATCCCCTGCATGAACACTTTCAACTTCCTGTCAGATTGAAAGTGTTCATGCAGGTTTTTG**
***sh-DHRS3b* antisense**	**AATTCAAAAACCTGCATGAACACTTTCAATCTGACAGGAAGTTGAAAGTGTTCATGCAGGG**
***sh-CTRL2* sense**	**GATCCCAATGCGCAAGAAGATCAACTTCCTGTCAGATTGATCTTCTTGCGCATTGTTTTTG**
***sh-CTRL2* antisense**	**AATTCAAAAACAATGCGCAAGAAGATCAATCTGACAGGAAGTTGATCTTCTTGCGCATTGG**

Table S2 *Structure of the double stranded DNAs used for the construction of the shRNA plasmid constructs*

The sequences of the sense and antisense strands of the oligonucleotides targeting the *IRF1* and *DHRS3* genes as well as the scramble control oligonucleotides (*CTRL1* and *CTRL2*) are illustrated. The nucleotides marked in red constitute the *EcoRI* and *BamH1* sites used for the insertion of the double-stranded oligonucleotides into the *pGreenPuro* plasmid. The nucleotides marked in black represent the sequences corresponding to different targeted regions of the *IRF1* (*shIRF1a* = exon 4, nucleotides 4-22; *shIRF1b* = exon 6, nucleotides 18-36) and *DHRS3* genes (*shDHRS3a* = exon 3, nucleotides 54-72; *shDHRS3b* = exon 6, nucleotides 54-72). In *shCTRL1* and *shCTRL2* the nucleotides marked in black are scrambled and non-targeting oligonucleotides. In each oligonucleotide the black sequences are separated by an *L12* loop marked in green.

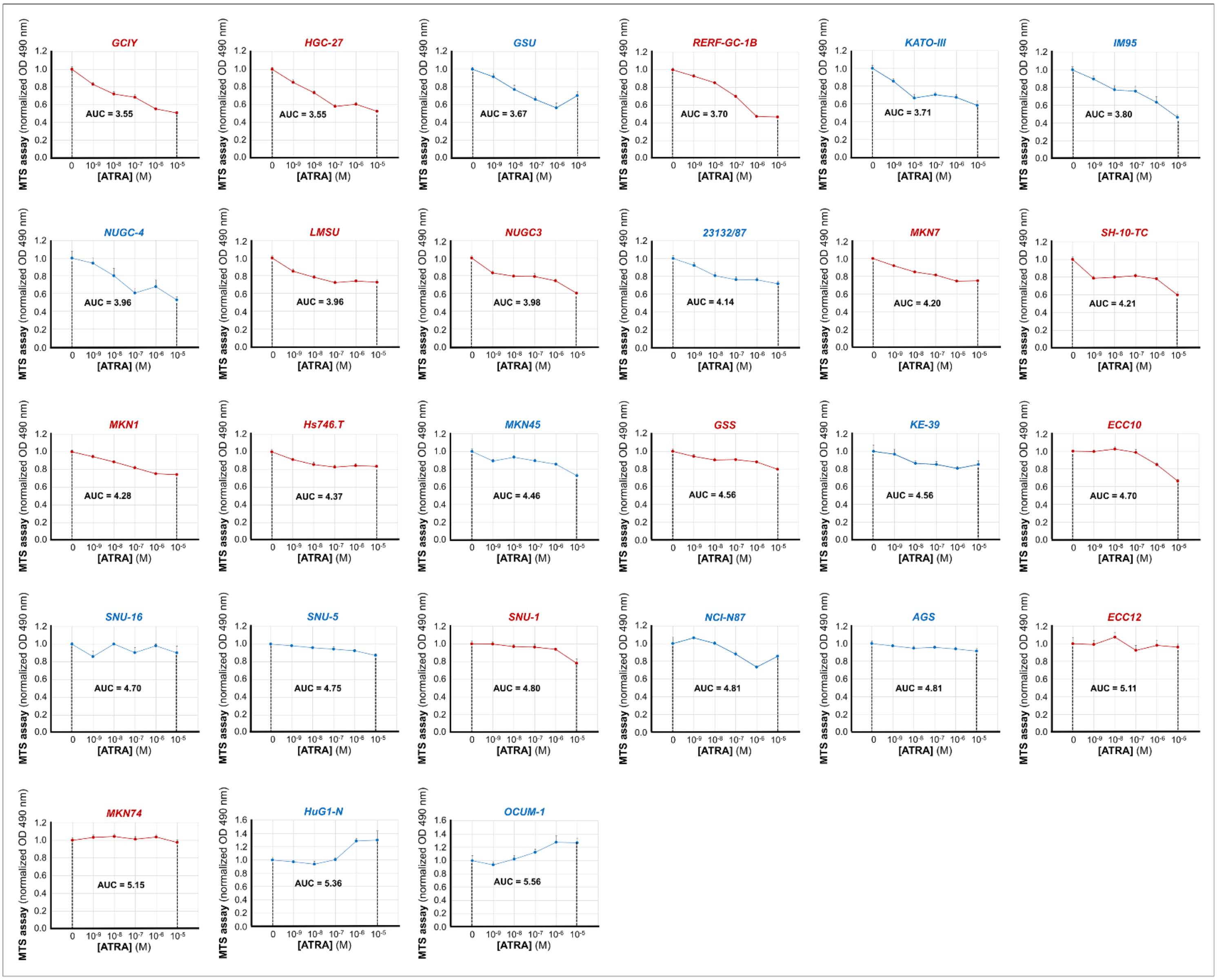

Figure S1 *Growth curves of the gastric cancer cell-lines exposed to increasing concentrations of ATRA*

The indicated and exponentially growing cell-lines were exposed to vehicle or 5 increasing concentrations of ATRA (10^{-9} to 10^{-5} M) for 6 days. At the end of the treatment, cell-lines were subjected to MTS assays to determine their growth. Each value represents the Mean $\pm$ SE of 10 independent cultures with the exception of *HuG1-N*, *OCUM-1*, *SNU-1*, *SNU-5* and *SNU-16* cells where each value is the Mean $\pm$ SE of 4 independent cultures. The *Area-Under-the-Curve* (*AUC*) value, which was used for the calculation of the *ATRA-score*s, is indicated for each growth curve.

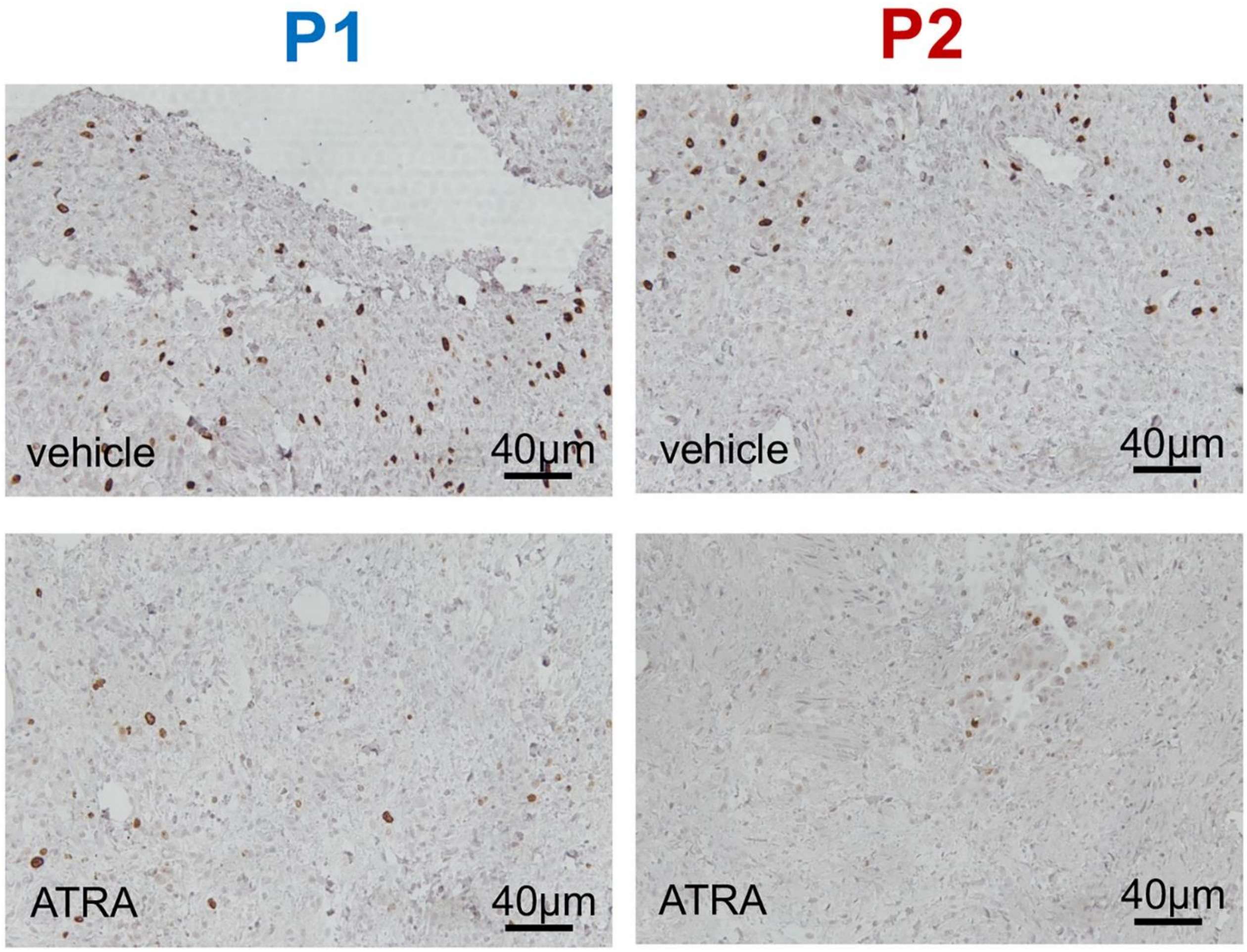

Figure S2 *Ki67 immune-histochemistry in tissue-slice cultures of representative primary gastric-cancers exposed to ATRA*

Tissue slices deriving from surgical specimens of 13 separate patients (P1-P13) were challenged with vehicle (DMSO) or ATRA (1.0 µM) for 48 hours. Tumor slices were fixed, paraffin embedded, cut into 5 µm slices and stained for the *Ki67* protein using a specific antibody. The case characterized by a *G-INT* phenotype is marked in blue, the case characterized by a *G-DIFF* phenotype is marked in red. The figure illustrates examples of the immuno-histochemical data obtained in two representative cases: (i) Patient 1 (P1), *G-INT* case; (ii) Patient 2 (P2), *G-DIFF* case.

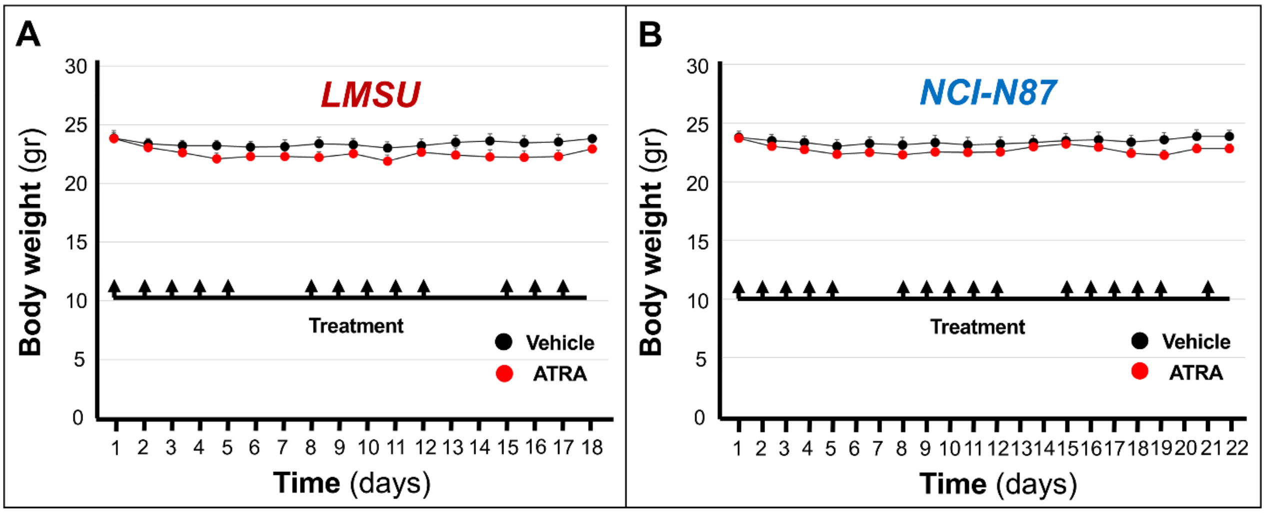

Figure S3 *Effects of ATRA on the body weight of SCID mice transplanted with LMSU and NCI-N87 cells*

Ten *Nude* mice/experimental group were xeno-transplanted with human *LMSU* (**A**) and *NCI-N87* (**B**) gastric-cancer cell-lines. Subsequently, mice were treated with vehicle (DMSO) or ATRA (15mg/kg) intra-peritoneally at the indicated time points (arrows). The total body weight of each animal was determined and the results are shown as the Mean$\pm$SD of the values.

	High Sensitivity							Low Sensitivity					
HALLMARK	GCIY	HGC-27	GSU	RERF-GC-1B	KATO-III	IM95	LMSU	MKN45	GSS	AGS	NCI-N87	HuG1-N	OCUM-1
ADIPOGENESIS	6.6 (0.2)	7.9 (0.2)	2.3 (0.6)	1.9 (0.6)	0.6 (0.9)	0.4 (0.9)	0.4 (0.9)	25.6 (0.01*)	9.1 (0.1)	1.9 (0.7)	0.6 (0.9)	2.4 (0.6)	44.5 (4.0E-05*)
ALLOGRAFT_REJECTION	19.8 (0.01*)	7.4 (0.2)	1.2 (0.8)	2.3 (0.6)	31.0 (0.01*)	0.4 (0.9)	8.1 (0.2)	0.3 (0.9)	0.1 (1.0)	7.2 (0.2)	3.6 (0.4)	2.4 (0.6)	0.8 (0.8)
ANDROGEN_RESPONSE	3.3 (0.5)	17.3 (0.02*)	1.1 (0.8)	0.9 (0.8)	1.5 (0.7)	3.7 (0.4)	0.4 (0.9)	7.0 (0.2)	0.1 (1.0)	2.4 (0.6)	1.5 (0.7)	3.6 (0.4)	15.4 (0.03*)
ANGIOGENESIS	4.9 (0.3)	24.1 (0.01*)	3.8 (0.4)	9.2 (0.1)	1.0 (0.8)	0.2 (0.9)	16.3 (0.02*)	1.4 (0.7)	6.3 (0.2)	3.4 (0.4)	3.6 (0.4)	2.4 (0.6)	2.7 (0.5)
APICAL_JUNCTION	23.3 (0.01*)	28.9 (0.01*)	26.9 (0.01*)	0.9 (0.8)	2.4 (0.6)	0.4 (0.9)	0.7 (0.9)	2.2 (0.6)	9.1 (0.1)	4.8 (0.3)	3.6 (0.4)	2.5 (0.6)	0.2 (1.0)
APICAL_SURFACE	8.9 (0.1)	4.3 (0.4)	1.3 (0.7)	8.0 (0.2)	1.2 (0.7)	0.1 (1.0)	3.1 (0.5)	1.6 (0.7)	1.7 (0.7)	5.5 (0.3)	3.6 (0.4)	1.0 (0.8)	4.4 (0.4)
APOPTOSIS	1.7 (0.7)	61.0 (7.8E-07*)	9.8 (0.1*)	13.5 (0.04*)	13.5 (0.05*)	0.2 (0.9)	16.5 (0.02*)	1.2 (0.8)	1.9 (0.6)	0.6 (2.3)	1.3 (0.7)	2.5 (0.6)	0.2 (0.9)
BILE_ACID_METABOLISM	0.4 (0.9)	4.9 (0.3)	0.4 (0.9)	2.4 (0.6)	1.2 (0.7)	2.9 (0.5)	0.3 (0.9)	15.1 (0.03*)	7.6 (0.2)	2.4 (0.6)	0.6 (0.9)	1.2 (0.8)	7.6 (0.2)
CHOLESTEROL_HOMEOSTASIS	33.8 (0.01*)	0.1 (0.9)	14.9 (0.03*)	4.5 (0.4)	1.0 (0.8)	6.1 (0.2)	13.0 (0.05*)	11.2 (0.07*)	2.7 (0.5)	46.4 (2.3E-05*)	5.4 (0.3)	7.9 (0.2)	7.6 (0.2)
COAGULATION	3.4 (0.5)	61.0 (7.8E-07*)	16.5 (0.02*)	2.3 (0.6)	12.9 (0.05*)	0.1 (1.0)	15.6 (0.03*)	2.2 (0.6)	0.1 (1.0)	2.4 (0.6)	6.0 (0.3)	1.3 (0.7)	4.4 (0.4)
COMPLEMENT	3.4 (0.5)	35.4 (0.01*)	11.6 (0.07*)	19.9 (0.01*)	21.7 (0.01*)	0.4 (0.9)	16.3 (0.02*)	0.2 (0.9)	1.5 (0.7)	0.1 (1.0)	3.5 (0.4)	1.6 (0.7)	3.6 (0.4)
DNA_REPAIR	24.9 (0.01*)	3.4 (0.5)	2.3 (0.6)	19.8 (0.01*)	4.1 (0.4)	0.1 (1.0)	4.8 (0.3)	2.2 (0.9)	0.2 (0.9)	0.3 (0.9)	1.0 (0.8)	2.4 (0.6)	4.6 (0.3)
E2F_TARGETS	147.3 (1.8E-15*)	58.5 (1.4E-06*)	1.6 (0.7)	218.2 (1.5E-22*)	53.6 (4.4E-06*)	13.6 (0.04*)	28.2 (0.01*)	2.2 (0.6)	8.7 (0.1)	0.1 (1.0)	36.0 (0.01*)	0.2 (1.0)	48.3 (1.0E-05*)
EPITHELIAL_MESENCHYMAL_TRANSITION	4.9 (0.3)	121.8 (6.5E-13*)	16.5 (0.02*)	15.3 (0.03*)	0.2 (0.9)	0.4 (0.9)	6.3 (0.2)	2.2 (0.6)	0.1 (1.0)	2.3 (0.6)	3.6 (0.4)	14.2 (0.04*)	0.4 (4.2)
ESTROGEN_RESPONSE_EARLY	2.7 (0.5)	35.2 (0.01*)	26.6 (0.01*)	32.1 (0.01*)	1.0 (0.8)	0.2 (0.9)	10.9 (0.08*)	2.2 (0.6)	25.8 (0.01*)	0.4 (0.9)	0.1 (1.0)	1.0 (0.8)	11.2 (0.08*)
ESTROGEN_RESPONSE_LATE	1.7 (0.7)	8.7 (0.1)	18.2 (0.01*)	10.7 (0.09*)	1.5 (0.7)	0.4 (0.9)	1.7 (0.7)	0.8 (0.8)	7.5 (0.2)	7.2 (0.2)	3.4 (0.5)	1.0 (0.8)	18.2 (0.02*)
FATTY_ACID_METABOLISM	2.7 (0.5)	21.9 (0.01*)	0.1 (0.9)	0.1 (1.0)	1.4 (0.7)	0.2 (0.9)	3.1 (0.5)	35.6 (0.01*)	2.7 (0.5)	7.2 (0.2)	2.1 (0.6)	2.2 (0.6)	46.7 (2.0E-05*)
G2M_CHECKPOINT	60.4 (9.1E-07*)	43.7 (0.01*)	1.3 (0.7)	144.7 (3.4E-15*)	53.6 (4.4E-06*)	24.6 (0.01*)	12.4 (0.06*)	8.6 (0.1)	9.1 (0.1)	0.9 (0.8)	31.9 (0.01*)	5.5 (0.3)	23.4 (0.01*)
GLYCOLYSIS	2.9 (0.5)	0.1 (0.9)	1.6 (0.7)	5.0 (0.3)	5.1 (0.3)	0.4 (0.9)	13.7 (0.04*)	2.3 (0.6)	7.8 (0.2)	0.3 (0.9)	0.8 (0.8)	0.6 (0.9)	7.6 (0.2)
HEDGEHOG_SIGNALING	34.0 (0.01*)	0.4 (0.9)	0.4 (0.9)	1.9 (0.6)	0.5 (0.9)	0.1 (1.0)	2.3 (0.6)	0.5 (0.9)	0.5 (0.9)	2.3 (0.6)	0.7 (0.8)	4.4 (0.4)	6.1 (0.2)
HEME_METABOLISM	7.9 (0.2)	2.6 (0.6)	4.1 (0.4)	2.2 (0.6)	1.4 (0.7)	0.1 (1.0)	0.8 (0.8)	1.4 (0.7)	0.1 (1.0)	3.4 (0.4)	0.6 (0.9)	2.2 (0.6)	3.5 (0.4)
HYPOXIA	3.4 (0.5)	28.5 (0.01*)	2.4 (0.6)	0.3 (0.9)	1.0 (0.8)	0.2 (0.9)	7.9 (0.2)	2.2 (0.6)	6.7 (0.2)	0.3 (0.9)	3.4 (0.5)	0.2 (1.0)	1.0 (0.8)
IL2_STAT5_SIGNALING	3.4 (0.5)	47.6 (0.01*)	3.8 (0.4)	5.9 (0.3)	0.2 (0.9)	0.4 (0.9)	8.0 (0.2)	0.5 (0.9)	3.7 (0.4)	2.3 (0.6)	3.6 (0.4)	2.5 (0.6)	0.2 (0.9)
IL6_JAK_STAT3_SIGNALING	2.7 (0.5)	17.5 (0.02*)	1.6 (0.7)	3.3 (0.5)	6.9 (0.2)	0.4 (0.9)	28.2 (0.01*)	1.7 (0.7)	2.3 (0.6)	2.1 (0.6)	3.6 (0.4)	2.3 (0.6)	0.7 (0.8)
INFLAMMATORY_RESPONSE	7.7 (0.2)	11.2 (0.08*)	2.4 (0.6)	4.5 (0.4)	1.5 (0.7)	0.1 (1.0)	16.5 (0.02*)	2.2 (0.6)	3.3 (0.5)	8.0 (0.2)	3.6 (0.4)	2.3 (0.6)	1.5 (0.7)
INTERFERON_ALPHA_RESPONSE	45.4 (0.01*)	47.6 (0.01*)	0.4 (0.9)	35.7 (0.01*)	132.1 (6.2E-14*)	0.4 (0.9)	34.1 (0.01*)	1.4 (0.7)	9.1 (0.1)	1.7 (0.7)	66.9 (2.0E-07*)	11.6 (0.07*)	1.4 (0.7)
INTERFERON_GAMMA_RESPONSE	49.7 (0.01*)	32.6 (0.01*)	0.4 (0.9)	29.6 (0.01*)	122.1 (6.1E-13*)	0.4 (0.9)	55.1 (3.1E-06*)	2.0 (0.6)	7.5 (0.2)	2.7 (0.5)	46.1 (2.0E-05*)	7.9 (0.2)	2.4 (0.6)
KRAS_SIGNALING_DN	6.1 (0.2)	0.9 (0.8)	3.8 (0.4)	1.9 (0.6)	2.2 (0.6)	4.4 (0.4)	3.1 (0.5)	1.4 (0.7)	0.7 (0.8)	19.9 (0.01*)	0.6 (0.9)	2.8 (0.5)	5.3 (0.3)
KRAS_SIGNALING_UP	2.7 (0.5)	15.4 (0.03*)	2.4 (0.6)	11.9 (0.06*)	10.4 (0.1*)	0.1 (1.0)	13.0 (0.05*)	0.5 (0.9)	2.5 (0.6)	8.0 (0.2)	3.6 (0.4)	1.3 (0.7)	2.7 (0.5)
MITOTIC_SPINDLE	5.3 (0.3)	1.9 (0.6)	1.3 (0.7)	15.9 (0.03*)	0.2 (0.9)	0.4 (0.9)	0.4 (0.9)	2.2 (0.6)	6.3 (0.2)	1.9 (0.6)	0.6 (0.9)	0.2 (1.0)	0.8 (0.8)
MTORC1_SIGNALING	8.9 (0.1)	10.3 (0.09*)	3.9 (0.4)	16.2 (0.02*)	0.9 (0.8)	4.2 (0.4)	3.1 (0.5)	0.2 (1.0)	0.2 (0.9)	12.5 (0.06)	6.7 (0.2)	4.7 (0.3)	42.6 (5.0E-05*)
MYC_TARGETS_V1	125.2 (3.0E-13*)	42.3 (0.01*)	1.6 (0.7)	93.4 (4.5E-10*)	77.2 (1.9E-08*)	4.4 (0.4)	13.2 (0.05*)	0.5 (0.9)	3.7 (0.4)	71.1 (7.7E-8*)	64.9 (3.0E-07*)	3.1 (0.5)	67.6 (2.0E-07*)
MYC_TARGETS_V2	144.8 (3.3E-15*)	30.2 (0.01*)	2.4 (0.6)	33.6 (0.01*)	113.6 (4.4E-12*)	24.6 (0.01*)	7.7 (0.2)	2.2 (0.6)	9.1 (0.1)	71.1 (7.7E-8*)	44.1 (4.0E-05*)	2.5 (0.6)	55.9 (3.0E-06*)
MYOGENESIS	17.1 (0.02*)	33.1 (0.01*)	10.0 (0.1*)	0.4 (0.9)	0.1 (1.0)	0.4 (0.9)	1.1 (0.8)	1.4 (0.7)	3.2 (0.5)	8.0 (0.2)	1.9 (0.6)	2.9 (0.5)	4.4 (0.4)
NOTCH_SIGNALING	2.7 (0.5)	4.3 (0.4)	10.2 (0.1*)	15.3 (0.03*)	0.6 (0.9)	0.4 (0.9)	2.3 (0.6)	0.5 (0.9)	1.6 (0.7)	14.9 (0.03*)	3.6 (0.4)	1.0 (0.8)	0.8 (0.8)
OXIDATIVE_PHOSPHORYLATION	27.0 (0.01*)	61.1 (7.8E-07*)	1.6 (0.7)	10.2 (0.09*)	0.2 (0.9)	0.1 (1.0)	8.6 (0.1)	35.6 (0.01*)	8.7 (0.1)	3.1 (0.5)	1.0 (0.8)	7.9 (0.2)	111.7 (7.0E-12*)
P53_PATHWAY	2.6 (0.5)	10.1 (0.1*)	3.8 (0.4)	3.0 (0.5)	0.1 (1.0)	0.4 (0.9)	0.4 (0.9)	1.7 (0.7)	3.9 (0.4)	0.1 (1.0)	2.6 (0.6)	1.0 (0.8)	0.2 (0.9)
PANCREAS_BETA_CELLS	3.4 (0.5)	2.9 (0.5)	10.0 (0.1*)	0.4 (0.9)	6.1 (0.2)	13.6 (0.04*)	10.9 (0.08*)	1.7 (0.7)	4.2 (0.4)	4.3 (0.4)	3.6 (0.4)	1.6 (0.7)	14.8 (0.03*)
PEROXISOME	6.1 (0.2)	0.6 (0.9)	1.3 (0.7)	0.9 (0.8)	5.3 (0.3)	0.1 (1.0)	2.6 (0.5)	8.6 (0.1)	3.7 (0.4)	3.0 (0.5)	1.0 (0.8)	4.4 (0.4)	18.7 (0.01*)
PI3K_AKT_MTOR_SIGNALING	3.4 (0.5)	2.3 (0.6)	9.8 (0.1*)	4.8 (0.3)	0.2 (0.9)	0.2 (0.9)	1.1 (0.8)	0.5 (0.9)	0.1 (1.0)	0.1 (1.0)	2.5 (0.6)	1.3 (0.7)	0.8 (0.8)
PROTEIN_SECRETION	4.9 (0.3)	28.5 (0.01*)	4.1 (0.4)	2.6 (0.5)	28.4 (0.01*)	2.9 (0.5)	1.1 (0.8)	1.4 (0.7)	9.1 (0.1)	1.9 (0.6)	2.6 (0.6)	0.1 (1.0)	3.6 (0.4)
REACTIVE_OXYGEN_SPECIES_PATHWAY	8.9 (0.1)	0.5 (0.9)	12.0 (0.06*)	2.6 (0.5)	0.1 (1.0)	0.2 (0.9)	0.5 (0.9)	5.8 (0.3)	1.9 (0.6)	3.2 (0.5)	3.6 (0.4)	4.7 (0.3)	7.6 (0.2)
SPERMATOGENESIS	8.1 (0.2)	7.9 (0.2)	1.1 (0.8)	9.9 (0.1*)	2.9 (0.5)	3.0 (0.5)	1.9 (0.6)	1.4 (0.7)	3.3 (0.5)	4.0 (0.4)	3.6 (0.4)	2.2 (0.6)	0.7 (0.8)
TGF_BETA_SIGNALING	8.1 (0.2)	39.7 (0.01*)	16.5 (0.02*)	0.3 (0.9)	0.5 (0.9)	0.1 (1.0)	12.4 (0.06*)	2.2 (0.6)	2.1 (0.6)	0.1 (1.0)	0.6 (0.9)	1.0 (0.8)	9.9 (0.1)
TNFA_SIGNALING_VIA_NFKB	2.2 (0.6)	48.1 (0.01*)	18.2 (0.02*)	2.6 (0.5)	1.1 (0.8)	0.2 (0.9)	55.1 (3.1E-06*)	2.2 (0.6)	7.8 (0.2)	41.0 (7.8E-05*)	4.5 (0.4)	3.0 (0.5)	10.0 (0.1)
UNFOLDED_PROTEIN_RESPONSE	3.8 (0.4)	0.1 (0.9)	1.9 (0.6)	9.2 (0.1)	1.5 (0.7)	0.1 (1.0)	14.3 (0.04*)	2.2 (0.6)	8.7 (0.1)	30.2 (9.6E-04*)	3.6 (0.4)	0.2 (1.0)	18.7 (0.01)
UV_RESPONSE_DN	21.9 (0.01*)	53.0 (4.9E-06*)	0.5 (0.9)	2.6 (0.5)	5.3 (0.3)	6.1 (0.2)	3.1 (0.5)	0.5 (0.9)	0.1 (1.0)	1.9 (0.6)	1.0 (0.8)	0.1 (1.0)	2.6 (0.5)
UV_RESPONSE_UP	21.8 (0.01*)	5.9 (0.2)	5.9 (0.3)	0.9 (0.8)	0.5 (0.9)	0.1 (1.0)	0.7 (0.9)	1.9 (0.6)	7.5 (0.2)	0.1 (1.0)	1.9 (0.6)	2.9 (0.5)	3.6 (0.4)
WNT_BETA_CATENIN_SIGNALING	3.3 (0.5)	4.3 (0.4)	1.6 (0.7)	13.9 (0.04*)	10.4 (0.09*)	0.4 (0.9)	0.5 (0.9)	1.7 (0.7)	0.1 (1.0)	3.0 (0.5)	33.5 (0.01*)	2.3 (0.6)	15.1 (0.03*)
XENOBIOTIC_METABOLISM	2.6 (0.5)	0.6 (0.9)	3.8 (0.4)	10.7 (0.08*)	9.9 (0.1*)	0.1 (1.0)	3.1 (0.5)	8.6 (0.1)	5.4 (0.3)	2.3 (0.6)	1.9 (0.6)	7.9 (0.2)	14.0 (0.04*)

Figure S4 *HALLMARK pathway analysis of the RNA-seq results obtained following treatment of the indicated gastric cell lines with ATRA*

Exponentially growing cultures of the indicated cell lines were exposed to ATRA (1.0 µM) for 48 hours. At the end of the treatment cells were subjected to *RNA-seq* analysis (**Supplementary Table S3**). The data obtained were subjected to pathway analysis using the *HALLMARK* data set. The numbers shown indicate the *Score* values obtained. The *FDR* (*False-Discovery-Rate*) values are indicated in parenthesis. When the *FDR* values are <0.1, they are considered to be statistically significant and they are marked in red with an asterisk. When the statistically significant pathways are up-regulated they are contained in a pink box. By contrast, the down-regulated pathways are contained in light blue box. The most relevant up- (red) or down-regulated (blue) pathways are contained in a yellow box.

	High Sensitivity							Low Sensitivity					
KEGG Metabolism	GCIY	HGC27	GSU	RERF-GC-1B	KATO-III	IM95	LMSU	MKN45	GSS	AGS	NCI-N87	HuG1-N	OCUM-1
ALANINE ASPARTATE AND GLUTAMATE METABOLISM	0.3 (0.9)	5.6 (0.3)	4.0 (0.4)	0.1 (1.0)	0.7 (0.8)	0.3 (0.9)	2.3 (0.6)	0.1 (1.0)	1.2 (0.8)	2.1 (0.6)	3.4 (0.5)	0.4 (0.9)	3.2 (0.5)
ALPHA LINOLENIC ACID METABOLISM	0.8 (0.8)	0.7 (0.9)	1.6 (0.7)	4.3 (0.4)	0.2 (0.9)	0.3 (0.9)	1.8 (0.7)	0.8 (0.8)	2.9 (0.5)	0.2 (1.0)	01 (1.0)	0.4 (0.9)	7.3 (0.2)
AMINO SUGAR AND NUCLEOTIDE SUGAR METABOLISM	3.6 (0.4)	0.6 (0.9)	0.7 (0.8)	1.6 (0.7)	16.9 (0.02*)	0.1 (1.0)	11.7 (0.07*)	4.5 (0.4)	0.5 (0.9)	3.0 (0.5)	1.7 (0.7)	1.0 (0.8)	9.1 (0.1)
ARACHIDONIC ACID METABOLISM	0.6 (0.9)	1.2 (0.8)	5.2 (0.3)	1.6 (0.7)	1.8 (0.7)	0.1 (1.0)	11.0 (0.08*)	0.1 (1.0)	3.7 (0.4)	1.4 (0.7)	8.7 (0.1)	2.60 (0.5)	0.8 (0.8)
ARGININE AND PROLINE METABOLISM	0.1 (1.0)	12.3 (0.06*)	1.0 (0.8)	4.3 (0.4)	5.1 (0.3)	0.1 (1.0)	10.0 (0.1)	0.4 (0.9)	2.6 (0.5)	1.3 (0.7)	1.5 (0.7)	0.3 (0.9)	13.5 (0.04*)
ASCORBATE AND ALDARATE METABOLISM	5.2 (0.3)	6.7 (0.2)	1.5 (0.7)	4.6 (0.3)	0.2 (0.9)	27.6 (1.7E-0.3*)	1.0 (0.8)	12.6 (0.06*)	0.2 (0.9)	0.1 (1.0)	4.5 (0.4)	9.8 (0.1)	29.7 (1.1E-03*)
BETA ALANINE METABOLISM	2.7 (0.5)	5.9 (0.3)	1.8 (0.7)	0.4 (0.9)	0.3 (0.9)	0.1 (1.0)	0.5 (0.9)	4.7 (0.3)	0.3 (0.9)	1.4 (0.7)	0.4 (0.9)	3.1 (0.5)	6.0 (0.2)
BIOSYNTHESIS OF UNSATURATED FATTY ACIDS	0.1 (1.0)	0.1 (1.0)	0.4 (0.9)	4.3 (0.4)	0.3 (0.9)	0.1 (1.0)	0.5 (0.9)	1.6 (0.7)	2.3 (0.6)	0.2 (1.0)	1.8 (0.7)	0.4 (0.9)	15.8 (0.03*)
BUTANOATE METABOLISM	2.7 (0.5)	17.7 (0.02*)	0.2 (1.0)	5.1 (0.3)	1.0 (0.8)	6.0 (0.3)	5.8 (0.3)	25.6 (2.7E-03)	0.2 (0.9)	12.6 (0.06*)	4.5 (0.4)	2.8 (0.5)	29.7 (1.1E-03*)
CITRATE CYCLE TCA CYCLE	0.8 (0.8)	41.9 (6.0E-05*)	2.4 (0.6)	6.3 (0.2)	2.1 (0.6)	8.3 (0.1)	3.3 (0.5)	7.4 (0.2)	3.7 (0.4)	0.1 (1.0)	1.7 (0.7)	0.4 (0.9)	51.3 (7.0E-06*)
CYSTEINE AND METHIONINE METABOLISM	0.3 (0.9)	11.4 (0.07*)	1.5 (0.7)	0.9 (0.8)	6.9 (0.2)	0.3 (0.9)	0.6 (0.9)	0.6 (0.9)	1.6 (0.7)	5.2 (0.3)	2.8 (0.5)	0.4 (0.9)	7.2 (0.2)
DRUG METABOLISM CYTOCHROME P450	0.1 (1.0)	0.1 (1.0)	1.5 (0.7)	1.1 (0.8)	16.9 (0.02*)	28.9 (1.3E-03*)	0.3 (0.9)	53.0 (5.0E-06*)	0.1 (1.0)	3.0 (0.5)	8.4 (0.1)	36.0 (2.0E-04*)	23.6 (4.3E-03*)
DRUG METABOLISM OTHER ENZYMES	0.3 (0.9)	0.8 (0.8)	1.9 (0.6)	1.0 (0.8)	1.9 (0.6)	5.8 (0.3)	0.1 (1.0)	4.1 (0.4)	2.6 (0.6)	0.1 (1.0)	3.7 (0.4)	4.7 (0.3)	23.6 (4.3E-03*)
ENDOCYTOSIS	1.8 (0.6)	13.7 (0.04*)	5.9 (0.3)	0.8 (0.8)	7.3 (0.2)	0.1 (1.0)	1.4 (0.7)	0.6 (0.9)	1.7 (0.7)	0.9 (0.8)	1.7 (0.7)	0.8 (0.8)	0.8 (0.8)
ETHER LIPID METABOLISM	4.2 (0.4)	0.7 (0.9)	2.8 (0.5)	0.4 (0.9)	0.4 (0.9)	0.3 (0.9)	1.4 (0.7	5.3 (0.3)	1.6 (0.7)	1.0 (0.8)	0.9 (0.8)	0.4 (0.9)	1.0 (0.8)
FATTY ACID METABOLISM	1.3 (0.7)	13.7 (0.04*)	9.8 (0.1)	4.3 (0.4)	5.6 (0.3)	17.9 (0.02*)	0.5 (0.9)	74.3 (3.7E-08*)	3.7 (0.4)	0.1 (1.0)	1.4 (0.7)	3.9 (0.4)	51.3 (7.0E-06*)
FOLATE BIOSYNTHESIS	6.7 (0.2)	3.4 (0.5)	0.2 (1.0)	1.9 (0.6)	0.2 (0.9)	0.1 (1.0)	5.7 (0.3)	1.8 (0.7)	5.5 (0.3)	0.1 (1.0)	1.3 (0.7)	3.0 (0.5)	9.8 (0.1)
FRUCTOSE AND MANNOSE METABOLISM	1.9 (0.6)	16.1 (0.02*)	0.5 (0.9)	0.1 (1.0)	0.3 (0.9)	0.6 (0.9)	8.1 (0.2)	0.9 (0.8)	2.9 (0.5)	1.6 (0.7)	0.1 (1.0)	0.4 (0.9)	9.8 (0.1)
GALACTOSE METABOLISM	3.9 (0.4)	1.3 (0.7)	1.0 (0.8)	6.4 (0.2)	9.6 (0.1)	0.3 (0.9)	6.3 (0.2)	4.1 (0.4)	10.5 (0.09*)	1.6 (0.7)	2.9 (0.5)	0.9 (0.8)	3.8 (0.4)
GLUTATHIONE METABOLISM	1.9 (0.6)	1.9 (0.7)	4.9 (0.3)	6.4 (0.2)	6.5 (0.2)	0.1 (1.0)	2.3 (0.6)	11.0 (0.08*)	0.2 (1.0)	0.3 (0.9)	3.7 (0.4)	21.0 (7.9E-03*)	9.2 (0.1)
GLYCEROLIPID METABOLISM	2.7 (0.5)	2.3 (0.6)	1.8 (0.7)	5.1 (0.3)	0.9 (0.8)	0.6 (0.9)	1.4 (0.7)	11.0 (0.08*)	3.7 (0.4)	1.1 (0.8)	2.4 (0.6)	0.9 (0.8)	20.9 (8.2E-03*)
GLYCEROPHOSPHOLIPID METABOLISM	19.9 (0.01*)	1.2 (0.8)	0.8 (0.8)	4.0 (0.4)	7.3 (0.2)	2.4 (0.6)	4.9 (0.3)	18.8 (0.01*)	13.1 (0.05*)	0.1 (1.0)	5.7 (0.3)	2.4 (0.6)	9.3 (0.1)
GLYCINE SERINE AND THREONINE METABOLISM	16.2 (0.02*)	7.3 (0.2)	3.3 (0.5)	0.5 (0.9)	21 (0.6)	0.8 (0.8)	2.7 (0.5)	0.1 (1.0)	0.5 (0.9)	0.1 (1.0)	0.3 (0.9)	0.3 (0.9)	10.0 (0.01*)
GLYCOLYSIS GLUCONEOGENESIS	2.7 (0.5)	17.9 (0.02*)	5.6 (0.3)	1.0 (0.8)	0.9 (0.8)	0.3 (0.9)	7.1 (0.2)	10.3 (0.09*)	4.9 (0.3)	1.0 (0.8)	1.7 (0.7)	3.9 (0.4)	35.7 (3.0E-04*)
GLYCOSAMINOGLYCAN BIOSYNTHESIS CHONDROITIN SULFATE	0.3 (0.9)	8.9 (0.1)	1.2 (0.8)	2.2 (0.6)	0.2 (0.9)	0.1 (1.0)	0.5 (0.9)	0.1 (1.0)	4.9 (0.3)	2.0 (0.6)	1.8 (0.7)	0.8 (0.8)	17.4 (0.02*)
GLYCOSAMINOGLYCAN BIOSYNTHESIS HEPARAN SULFATE	2.6 (0.5)	3.3 (0.5)	2.4 (0.6)	1.1 (0.8)	0.1 (1.0)	0.1 (1.0)	0.9 (0.8)	0.3 (0.9)	0.4 (0.9)	3.0 (0.5)	0.3 (0.9)	2.0 (0.6)	0.3 (0.9)
GLYCOSAMINOGLYCAN BIOSYNTHESIS KERATAN SULFATE	1.4 (0.7)	1.5 (0.7)	2.6 (0.5)	5.5 (0.3)	8.8 (0.1)	0.1 (1.0)	0.2 (0.9)	1.8 (0.7)	3.3 (0.5)	1.2 (0.8)	9.0 (0.1)	1.0 (0.8)	6.7 (0.2)
GLYCOSPHINGOLIPIDBIOSYNTHESIS GANGLIO SERIES	0.2 (1.0)	2.0 (0.6)	0.1 (1.0)	4.3 (0.4)	0.1 (1.0)	0.1 (1.0)	0.2 (1.0)	1.8 (0.7)	0.8 (0.8)	1.0 (0.8)	5.0 (0.3)	1.3 (0.7)	0.1 (1.0)
GLYCOSPHINGOLIPID BIOSYNTHESIS GLOBO SERIES	0.8 (0.8)	0.9 (0.8)	5.6 (0.3)	27.8 (1.7E-03*)	0.1 (1.0)	0.1 (1.0)	0.5 (0.9)	2.1 (0.6)	2.7 (0.5)	0.2 (1.0)	1.5 (0.7)	1.2 (0.8)	0.4 (0.9)
GLYCOSPHINGOLIPID BIOSYNTHESIS LACTO AND NEOLACTO SERIES	0.8 (0.8)	0.4 (0.9)	16.1 (0.02*)	10.3 (0.09*)	1.9 (0.6)	0.5 (0.9)	0.7 (0.8)	0.1 (1.0)	2.0 (0.6)	0.1 (1.0)	4.0 (0.4)	0.5 (0.9)	1.1 (0.8)
GLYCOSYLPHOSPHATIDYLINOSITOL GP ANCHOR BIOSYNTHESIS	1.7 (0.7)	3.6 (0.4)	2.6 (0.5)	4.3 (0.4)	0.3 (0.9)	0.1 (1.0)	3.6 (0.4)	0.4 (0.9)	3.3 (0.5)	0.1 (1.0)	1.2 (0.7)	0.1 (1.0)	1.3 (0.7)
KEGG GLYOXYLATE AND DICARBOXYLATE METABOLISM	0.1 (1.0)	14.7 (0.03*)	0.3 (0.9)	0.5 (0.9)	5.0 (0.3)	2.9 (0.5)	3.6 (0.4)	0.2 (1.0)	3.1 (0.5)	1.0 (0.8)	0.1 (1.0)	0.1 (1.0)	5.5 (0.3)
HISTIDINE METABOLISM	1.8 (0.7)	6.0 (0.2)	2.3 (0.6)	0.4 (0.9)	0.3 (0.9)	0.5 (0.9)	0.1 (1.0)	1.4 (0.7)	1.9 (0.6)	0.1 (1.0)	2.7 (0.5)	1.0 (0.8)	8.6 (0.1)
INOSITOL PHOSPHATE METABOLISM	1.3 (0.7)	4.7 (0.3)	1.8 (0.7)	8.6 (0.1)	2.1 (0.6)	0.3 (0.9)	2.3 (0.6)	0.1 (1.0)	3.3 (0.5)	0.6 (0.9)	1.7 (0.7)	2.6 (0.5)	0.1 (1.0)
LINOLEIC ACID METABOLISM	1.3 (0.7)	3.6 (0.4)	9.8 (0.1)	3.9 (0.4)	0.2 (0.9)	0.3 (0.9)	2.3 (0.6)	1.6 (0.7)	3.2 (0.5)	1.1 (0.8)	1.8 (0.7)	0.3 (0.9)	3.9 (0.4)
LYSINE DEGRADATION	8.8 (0.1)	14.3 (0.04*)	1.2 (0.8)	21.5 (7.0E-03*)	7.3 (0.2)	0.6 (0.9)	5.2 (0.3)	0.1 (1.0)	1.5 (0.7)	2.1 (0.6)	2.1 (0.6)	1.0 (0.8)	1.8 (0.7)
LYSOSOME	3.0 (0.5)	25.2 (3.0E-03*)	0.7 (0.9)	18.0 (1.6E-02*)	18.7 (0.01*)	0.5 (0.9)	0.2 (1.0)	3.2 (0.5)	0.3 (0.9)	52.8 (5.2E-06*)	11.0 (0.08)	4.2 (0.4)	0.2 (0.9)
METABOLISM OF XENOBIOTICS BY CYTOCHROME P450	0.8 (0.8)	0.7 (0.8)	1.2 (0.8)	0.4 (0.9)	26.8 (2.1E-03*)	54.0 (4.0E-06*)	1.4 (0.7)	46.1 (2.5E-05*)	0.8 (0.8)	3.0 (0.5)	9.0 (0.1)	48.8 (1.0E-05*)	28.8 (1.3E-03*)
N-GLYCAN BIOSYNTHESIS	18.4 (0.01*)	2.9 (0.5)	0.8 (0.8)	0.6 (0.9)	0.8 (0.8)	0.5 (0.9)	6.1 (0.2)	0.1 (1.0)	0.2 (1.0)	3.0 (0.5)	1.3 (0.7)	0.7 (0.9)	1.7 (0.7)
NICOTINATE AND NICOTINAMIDE METABOLISM	1.3 (0.7)	1.2 (0.8)	0.7 (0.8)	0.7 (0.8)	0.4 (0.9)	0.5 (0.9)	3.3 (0.5)	0.1 (1.0)	2.9 (0.5)	2.6 (0.5)	1.2 (0.8)	0.3 (0.9)	4.1 (0.4)
NITROGEN METABOLISM	0.2 (1.0)	12.5 (0.06*)	1.5 (0.7)	0.9 (0.8)	1.2 (0.8)	0.1 (1.0)	2.3 (0.6)	0.6 (0.9)	1.6 (0.7)	0.2 (1.0)	1.3 (0.7)	3.1 (0.5)	7.3 (0.2)
OTHER GLYCAN DEGRADATION	0.2 (1.0)	6.6 (0.2)	2.0 (0.6)	2.9 (0.5)	2.4 (0.6)	0.1 (1.0)	0.3 (0.9)	1.6 (0.7)	1.6 (0.7)	15.7 (2.7E-02*)	3.8 (0.4)	1.2 (0.8)	8.1 (0.2)
OXIDATIVE PHOSPHORYLATION	26.6 (2.2E-03)*)	37.5 (1.8E-04*)	15 (0.7)	5.1 (0.3)	3.3 (0.5)	0.1 (1.0)	5.7 (0.3)	29.8 (1.0E-03*)	2.9 (0.5)	1.0 (0.8)	0.2 (0.9)	3.9 (0.4)	51.3 (7.0E-06*)
PANTOTHENATE AND COA BIOSYNTHESIS	0.5 (0.9)	2.4 (0.6)	1.6 (0.7)	0.3 (0.9)	15.6 (0.03*)	0.5 (0.9)	0.3 (0.9)	0.4 (0.9)	0.6 (0.9)	0.1 (1.0)	1.4 (0.7)	3.1 (0.5)	1.8 (0.7)
PENTOSE AND GLUCURONATE INTERCONVERSIONS	4.2 (0.4)	0.9 (0.8)	1.8 (0.7)	0.6 (0.9)	0.8 (0.8)	6.7 (0.2)	0.2 (1.0)	8.8 (0.1)	0.6 (0.9)	1.9 (0.7)	4.5 (0.4)	12.6 (0.05*)	22.6 (5.4E-03*)
PENTOSE PHOSPHATE PATHWAY	4.1 (0.4)	5.5 (0.3)	1.6 (0.7)	0.4 (0.9)	0.1 (1.0)	0.3 (0.9)	1.8 (0.7)	0.5 (0.9)	10.6 (0.09*)	1.0 (0.8)	2.0 (0.6)	19.3 (0.01*)	16.4 (0.02*)
PEROXISOME	3.9 (0.4)	4.5 (0.3)	9.1 (0.1)	0.3 (0.9)	9.7 (0.1)	0.5 (0.9)	0.1 (1.0)	44.7 (3.4E-05*)	2.2 (0.6)	0.2 (1.0)	2.6 (0.5)	7.8 (0.2)	16.7 (0.02*)
PHENYLALANINE METABOLISM	1.3 (0.7)	5.6 (0.3)	9.1 (0.1)	1.7 (0.7)	3.3 (0.5)	0.5 (0.9)	2.3 (0.6)	0.3 (0.9)	1.6 (0.7)	1.0 (0.8)	2.6 (0.5)	0.4 (0.9)	4.2 (0.4)
PORPHYRIN AND CHLOROPHYLL METABOLISM	0.1 (1.0)	5.6 (0.3)	5.9 (0.3)	0.1 (1.0)	0.8 (0.8)	16.6 (0.02*)	3.3 (0.5)	13.2 (0.05*)	0.1 (1.0)	0.1 (1.0)	1.8 (0.7)	15.0 (0.03*)	35.3 (3.0E-04*)
PRIMARY BILE ACID BIOSYNTHESIS	2.0 (0.6)	1.2 (0.8)	2.1 (0.6)	5.1 (0.3)	5.1 (0.3)	0.1 (1.0)	3.8 (0.4)	1.6 (0.7)	2.3 (0.6)	3.0 (0.5)	0.2 (1.0)	1.3 (0.7)	5.6 (0.3)
PROPANOATE METABOLISM	10.2 (0.09*)	20.7 (8.4E-0.3*)	0.3 (0.9)	4.9 (0.3)	2.1 (0.6)	0.3 (0.9)	6.3 (0.2)	3.8 (0.4)	1.9 (0.6)	1.4 (0.7)	3.6 (0.4)	3.1 (0.5)	22.6 (5.4E-03*)
PURINE METABOLISM	23.7 (4.2E-03*)	5.6 (0.3)	1.6 (0.7)	1.8 (0.7)	0.9 (0.8)	0.5 (0.9)	3.6 (0.4)	0.1 (1.0)	3.5 (0.4)	3.5 (0.4)	1.5 (0.7)	1.0 (0.8)	9.2 (0.1)
PYRIMIDINE METABOLISM	30.3 (0.9E-04*)	5.6 (0.3)	1.0 (0.8)	6.3 (0.2)	4.4 (0.4)	0.5 (0.9)	7.1 (0.2)	0.1 (1.0)	3.7 (0.4)	2.7 (0.5)	0.2 (1.0)	2.6 (0.6)	13.8 (0.04*)
PYRUVATE METABOLISM	4.6 (0.3)	37.5 (1.8E-04*)	2.3 (0.6)	20.1 (9.7E-03*)	6.2 (0.2)	0.6 (0.9)	9.9 (0.1)	4.5 (0.4)	5.8 (0.3)	0.2 (1.0)	1.4 (0.7)	0.1 (1.0)	34.0 (4.0E-04*)
RETINOL METABOLISM	3.2 (0.5)	25.1 (3.0E-03*)	2.3 (0.6)	23.1 (4.9E-03*)	18.7 (0.01*)	48.4 (1.0E-05*)	34.1 (4.0E-04*)	55.5 (2.8E-06*)	1.6 (0.7)	0.1 (1.0)	4.5 (0.4)	36.0 (2.0E-04*)	34.0 (4.0E-04*)
RIBOFLAVIN METABOLISM	0.1 (1.0)	1.2 (0.8)	0.6 (0.9)	1.0 (0.8)	2.1 (0.6)	0.1 (1.0)	0.1 (1.0)	2.3 (0.6)	0.5 (0.9)	0.1 (1.0)	3.4 (0.5)	0.7 (0.8)	3.8 (0.4)
SELENOAMINO ACID METABOLISM	0.1 (1.0)	1.2 (0.8)	1.5 (0.7)	0.3 (0.9)	1.6 (0.7)	0.3 (0.9)	0.1 (1.0)	0.1 (1.0)	2.313062152	3.0 (0.5)	0.2 (0.9)	0.8 (0.8)	8.3 (0.1)
SPHINGOLIPID METABOLISM	4.1 (0.4)	8.3 (0.1)	8.0 (0.2)	4.3 (0.4)	0.6 (0.9)	0.1 (1.0)	0.2 (0.9)	0.1 (1.0)	2.9 (0.5)	5.2 (0.3)	1.8 (0.6)	0.3 (0.9)	0.3 (0.9)
STARCH AND SUCROSE METABOLISM	1.4 (0.7	2.3 (0.6)	2.8 (0.5)	2.9 (0.5)	6.6 (0.2)	14.4 (0.04*)	1.7 (0.7)	1.6 (0.7)	1.7 (0.7)	1.7 (0.7)	5.9 (0.3)	3.9 (0.4)	5.8 (0.3)
STEROID BIOSYNTHESIS	2.8 (0.5)	11.4 (0.07*)	0.4 (0.9)	5.0 (0.3)	3.6 (0.4)	8.9 (0.1)	7.5 (0.2)	22.8 (5.2E-03*)	0.4 (0.9)	76.0 (2.5E-08*)	8.7 (0.1)	4.3 (0.4)	9.2 (0.1)
STEROID HORMONE BIOSYNTHESIS	9.9 (0.1)	4.7 (0.3)	2.1 (0.6)	2.9 (0.5)	7.3 (0.2)	54.3 (4.0E-06*)	3.4 0.5)	3.3 (0.5)	0.8 (0.8)	1.5 (0.7)	4.3 (0.4)	8.6 (0.1)	22.9 (5.1E-03*)
SULFUR METABOLISM	1.8 (0.7)	13.7 (0.04*)	1.5 (0.7)	6.1 (0.2)	0.3 (0.9)	0.1 (1.0)	5.5 (0.3)	0.3 (0.9)	3.2 (0.5)	6.2 (0.2)	1.7 (0.7)	0.9 (0.8)	1.8 (0.7)
TAURINE AND HYPOTAURINE METABOLISM	2.7 (0.5)	2.7 (0.5)	1.6 (0.7)	3.0 (0.5)	1.3 (0.7)	0.1 (1.0)	2.2 (0.6)	0.6 (0.9)	2.0 (0.6)	7.6 (0.2)	2.6 (0.5)	2.1 (0.6)	8.6 (0.1)
TRYPTOPHAN METABOLISM	3.0 (0.5)	17.7 (0.02*)	0.1 (1.0)	1.6 (0.7)	1.0 (0.8)	0.1 (1.0)	2.3 (0.6)	4.7 (0.3)	1.6 (0.7)	1.0 (0.8)	0.6 (0.9)	0.8 (0.8)	11.4 (0.07*)
TYROSINE METABOLISM	2.7 (0.5)	4.8 (0.3)	0.1 (1.0)	2.6 (0.5)	0.3 (0.9)	0.3 (0.9)	3.4 (0.5)	9.3 (0.1)	1.1 (0.8)	0.2 (1.0)	2.6 (0.5)	2.0 (0.6)	3.2 (0.5)
VALINE LEUCINE AND ISOLEUCINE BIOSYNTHESIS	0.3 (0.9)	12.3 (0.06*)	1.0 (0.8)	4.3 (0.4)	0.3 (0.9)	0.8 (0.8)	7.1 (0.2)	2.1 (0.6)	1.6 (0.7)	31.6 (7.0E-04*)	2.6 (0.6)	0.4 (0.9)	13.6 (0.04*)
VALINE LEUCINE AND ISOLEUCINE DEGRADATION	7.7 (0.2)	35.3 (3.0E-04*)	5.5 (0.3)	3.3 (0.5)	0.2 (0.9)	0.1 (1.0)	6.4 (0.2)	32.3 (5.9E-04*)	0.2 (1.0)	1.6 (0.7)	3.0 (0.5)	1.0 (0.8)	27.2 (1.9E-03*)

Figure S5 *KEGG pathway analysis of the RNA-seq results obtained following treatment of the indicated gastric cell lines with ATRA*

Exponentially growing cultures of the indicated cell lines were exposed to ATRA (1.0 μM) for 48 hours. At the end of the treatment cells were subjected to *RNA-seq* analysis (**Supplementary Table S3**). The data obtained were subjected to pathway analysis using the *KEGG* metabolic data set. The numbers shown indicate the *Score* values obtained. The *FDR* (*False-Discovery-Rate*) values are indicated in parenthesis. When the *FDR* values are <0.1, they are considered to be statistically significant and they are marked in red with an asterisk. When the statistically significant pathways are up-regulated they are contained in a pinkbox. By contrast, the down-regulated pathways are contained in light blue box. The most relevant up-regulated (red) and down-regulated (blue) pathways are contained in a yellow box.

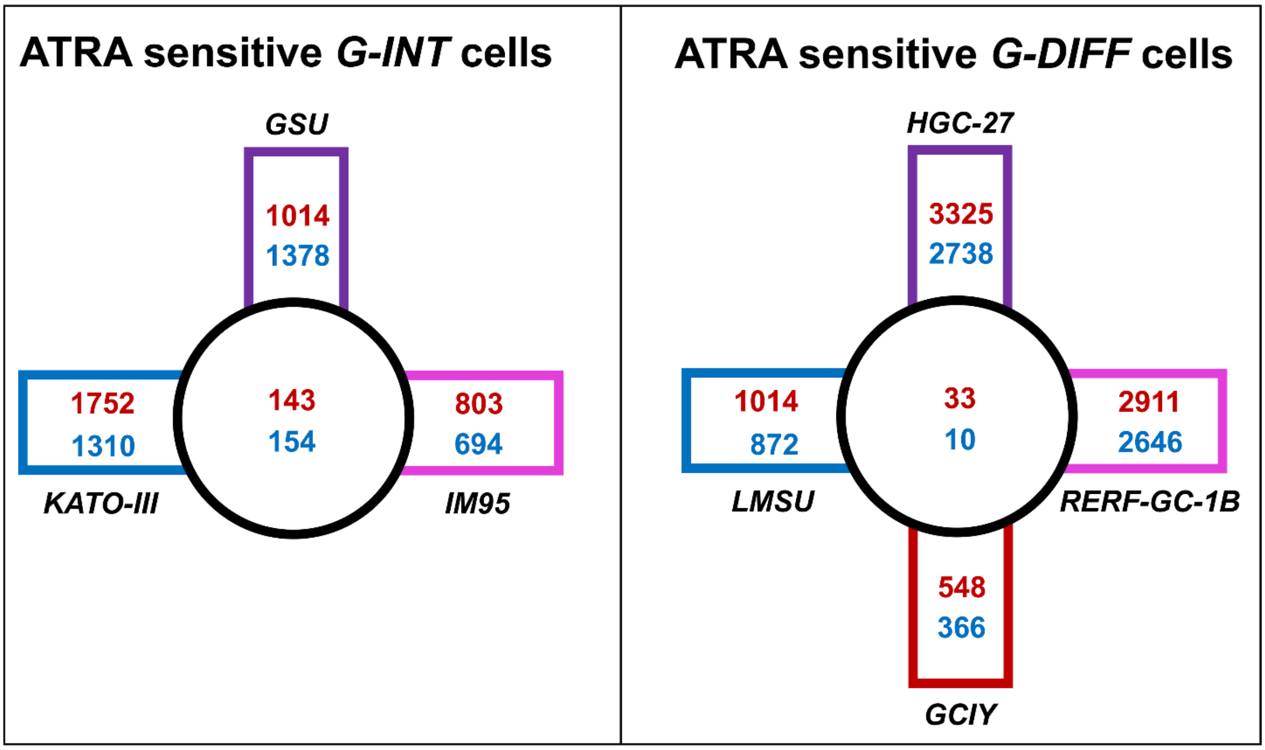

Figure S6 *Number of genes modulated by ATRA in retinoid-sensitive G-INT and G-DIFF gastric cancer cell-lines*

The *G-INT*/retinoid-sensitive *GSU*, *KATO-III* and *IM95* cell-lines as well as the *G-DIFF*/retinoid-sensitive *HGC-27*, *LMSU*, *GCIY* and *RERF-GC-1B* cell-lines were exposed to vehicle (DMSO) or ATRA (1.0 µM) for 48 hours. At the end of the treatment, cells were subjected to *RNA-seq* analysis. **Left:** The panel illustrates the number of genes selectively up-regulated (red) or down-regulated (blue) in each *G-INT* cell-line (squares) and commonly up-regulated (red) or down-regulated (blue) in the 3 cell-lines (circle). **Right:** The panel illustrates the number of genes selectively up-regulated (red) or down-regulated (blue) in each *G-DIFF* cell-line (squares) and commonly up-regulated (red) or down-regulated (blue) in the 4 cell-lines (circle).

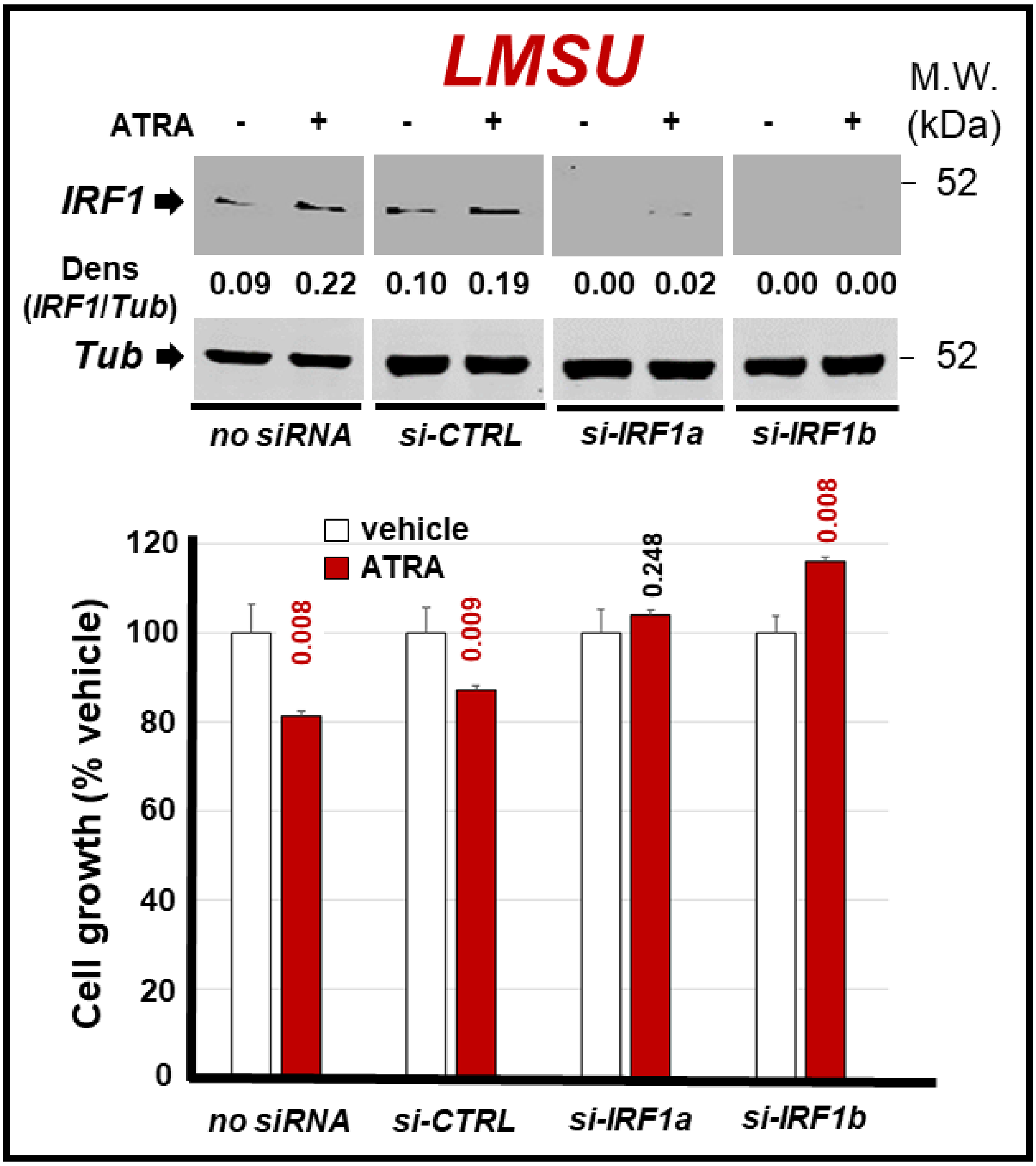

Figure S7 *Effects of ATRA on IRF1 protein levels and cell-growth in the retinoid-sensitive LMSU cell-line*

LMSU cells were transfected with two *IRF1*-targeting (*si-IRF1a*/*si-IRF1b*) and a control siRNA (*si-CTRL*). Twenty-four hours later, cells were treated with vehicle (DMSO) or ATRA (1µM) for 48 hours. **Upper**: Western-blot analysis using anti-*IRF1* and anti-tubulin antibodies: the lanes marked as "no-siRNA" indicate the parental *LMSU* cells. The values shown underneath the *IRF1* Western blots were obtained following densitometric analysis (Dens) of the *IRF1* and Tubulin (*Tub*) bands and represent the *IRF1*/*Tub* ratio, as indicated. **Lower**: Cell-growth of the transfected *LMSU* cells (sulforhodamine-assay): the results are expressed as the Mean±SD values of 3 replicate cultures, all the values are normalized for vehicle-treated cells (100%). The p-values (two-tailed Student's t-test) of the comparisons between ATRA-treated and vehicle-treated cells are shown above each red column. The p-values are marked in red if they indicate statistical significance. The figure shows the data obtained in one of the three independent experiments performed, which provided identical results.

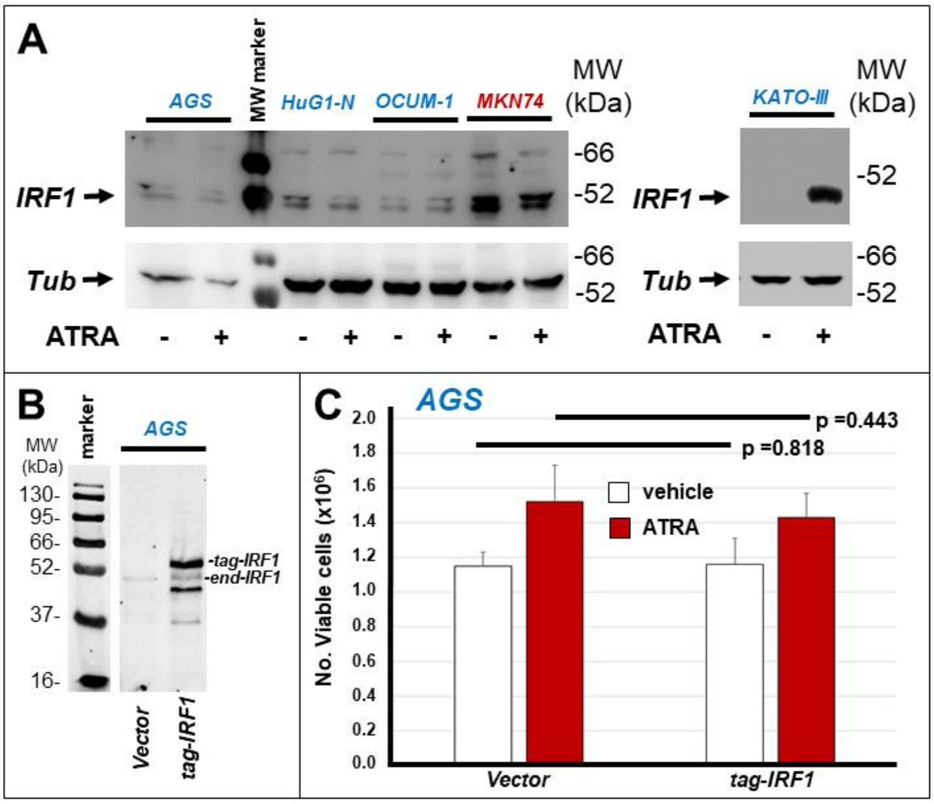

Figure S8 *Effects of ATRA on IRF1 protein expression in retinoid resistant gastric cancer cells and IRF1 over-expression in AGS cells*

(**A**) The *G-INT*, *OCUM-1*, *HuG1-N* and *AGS* cell-lines as well as the *G-DIFF*, *MKN-74* cell-line, which are characterized by a low level of sensitivity to the anti-proliferative effects of ATRA, were exposed to vehicle (DMSO) or ATRA (1.0 µM) for 48 hours. At the end of the treatment, cells were subjected to Western blot analysis with specific anti-*IRF1*and anti-tubulin (*Tub*) antibodies. The levels of tubulin in each lane of the gel are shown as a loading control. (**B**) Retinoid resistant *AGS* cells were transfected with a commercially available plasmid (myc-DDK-tagged human *IRF1*; Origene) allowing the expression of a tagged and biologically active form of the human *IRF1* transcription factor (*tag-IRF1*) or the corresponding void vector (*Vector*). *IRF1* expressing cells were selected in the presence of G418 (0.4 mg/ml) for 10 days. The Western blot shows the expression levels of the endogenous *IRF1* protein and the over-expressed *tag-IRF1* counterpart. (**C**) The entire *Vector* and *tag-IRF1* cell populations were plated at the same cell density (10^4 cells/ml) in a 12-wells plate (triplicate cultures). Cells were grown in *RPMI* medium supplemented with charcoal treated Fetal Bovine Serum (FBS) in the absence of G418 for 2 days. Subsequently cells were exposed to vehicle (DMSO) or 1.0 µM for 6 days. The number of viable cells was determined with the use of a Beckman Coulter Counter (Vi-CELL BLU v1.4.2). Each value is the Mean±SD of 6 independent cultures. The p-value of the ATRA *vs*. vehicle comparison is shown above each red column. The comparisons between the results obtained in vehicle treated *Vector* and *tag-IRF1* cells did not provide statistically significant results (p-value = 0.818). Similarly, the comparisons between the results obtained in ATRA treated *Vector* and *tag-IRF1* cells did not provide statistically significant results (p-value = 0.443), as shown in the panel.

ORIGINAL WESTERN BLOTS

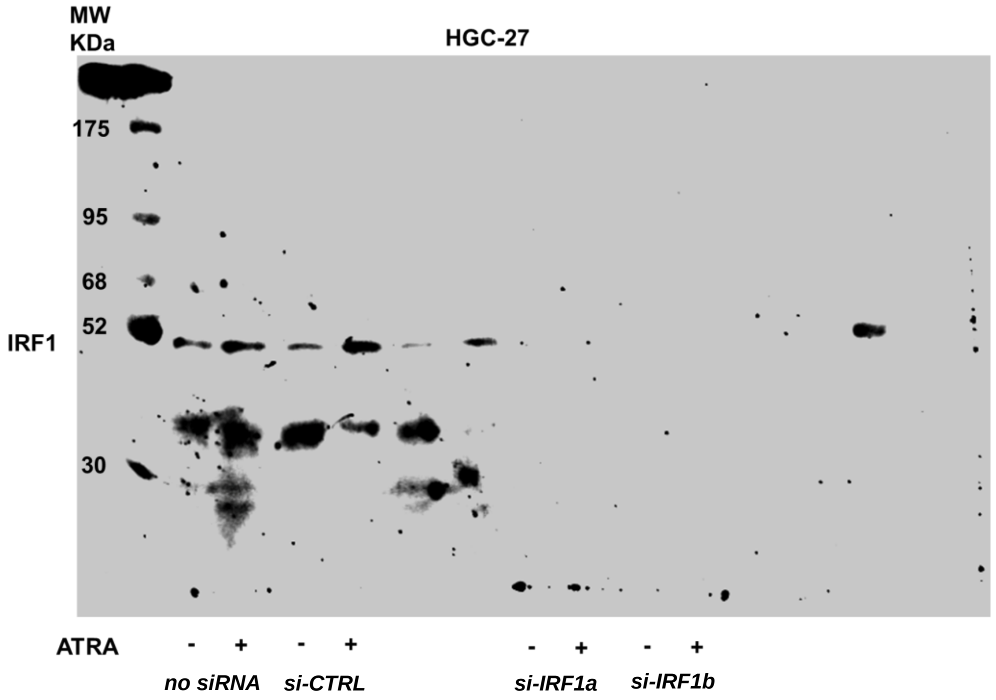

Original **Fig 8A**

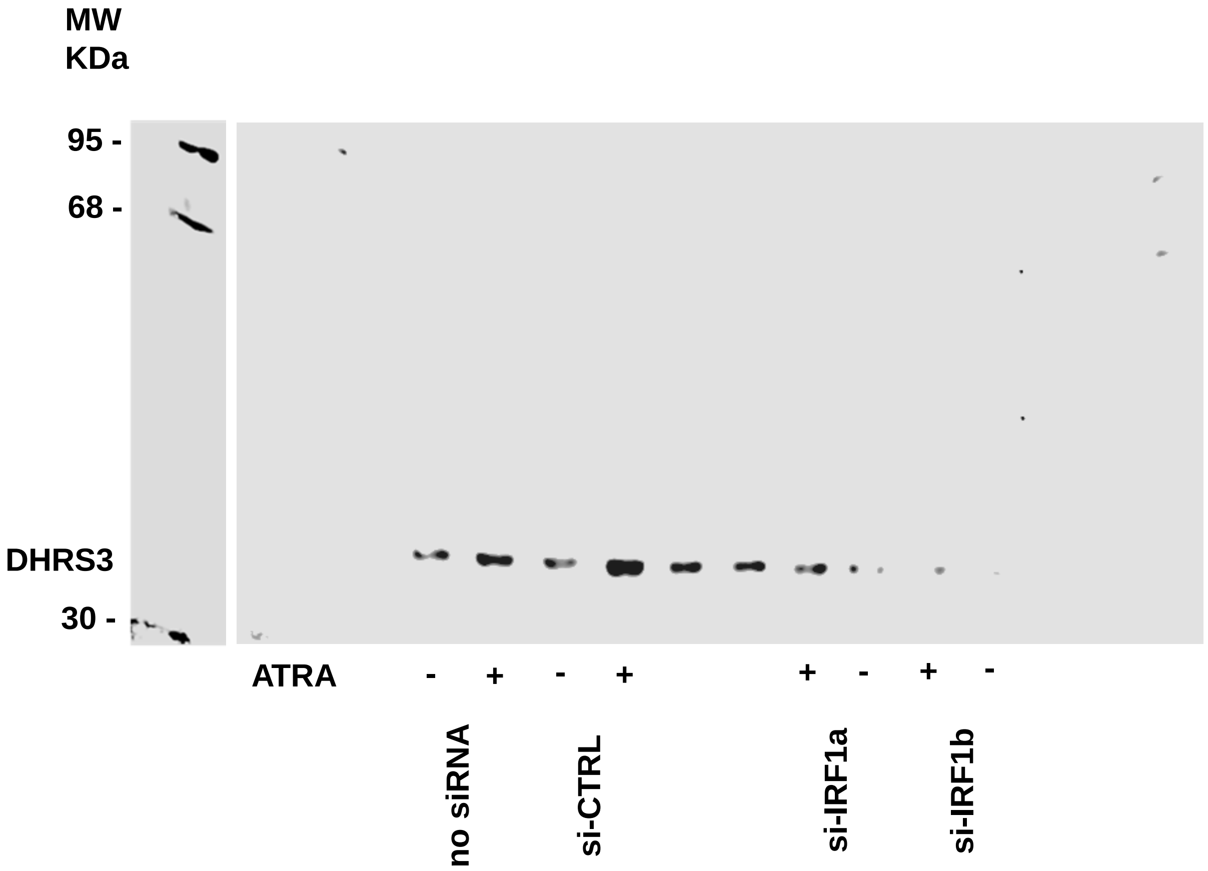

Original **Fig 8A**

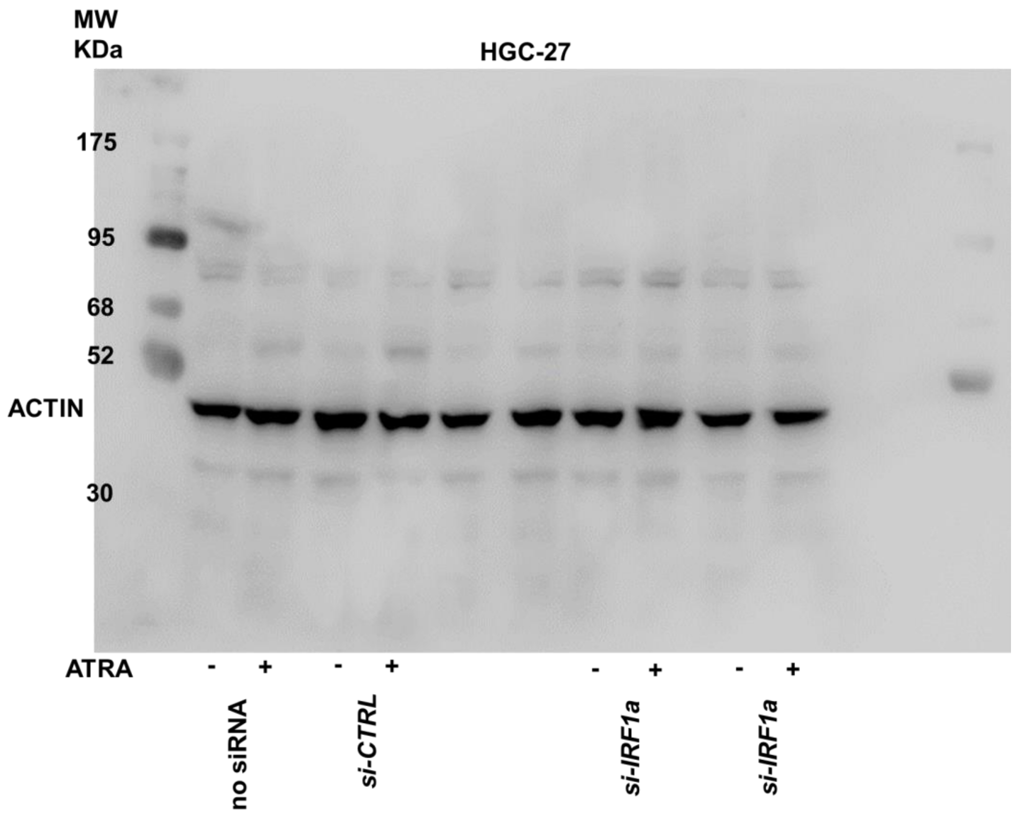

Original **Fig 8A**

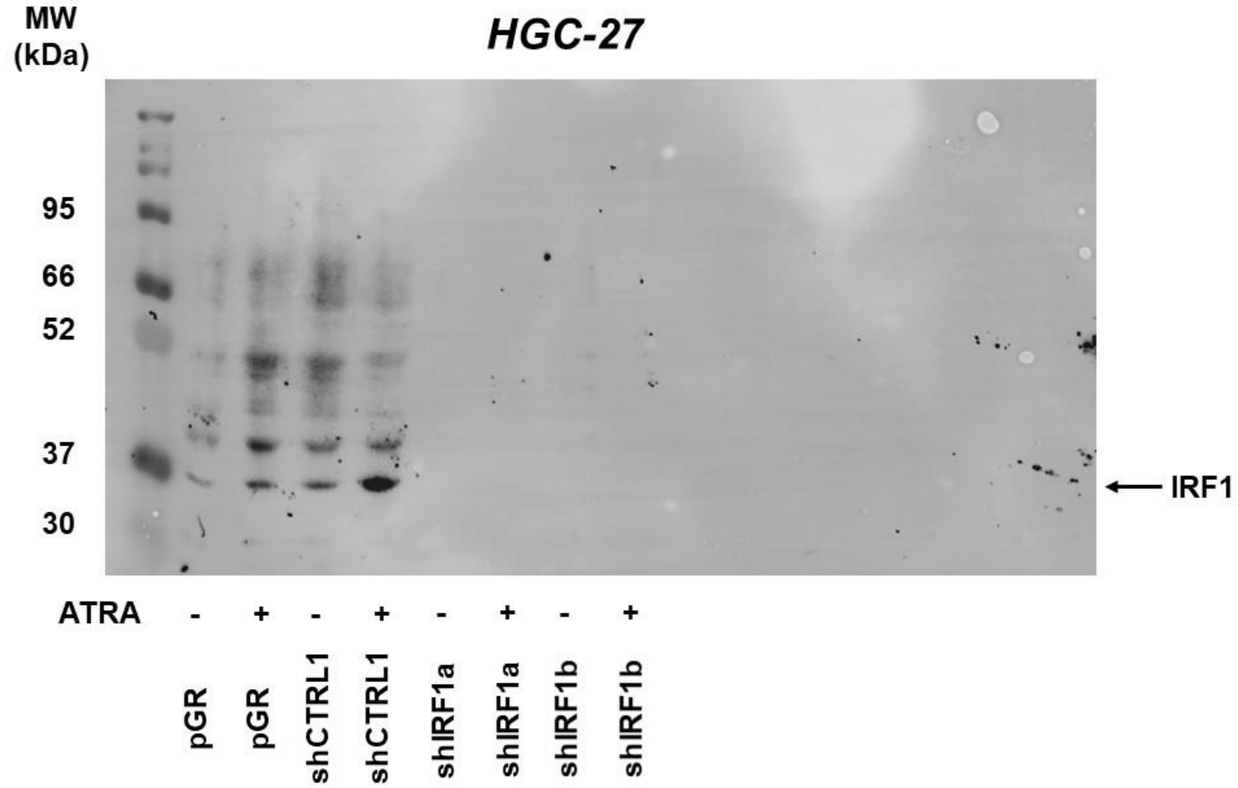

Original **Fig 8C**

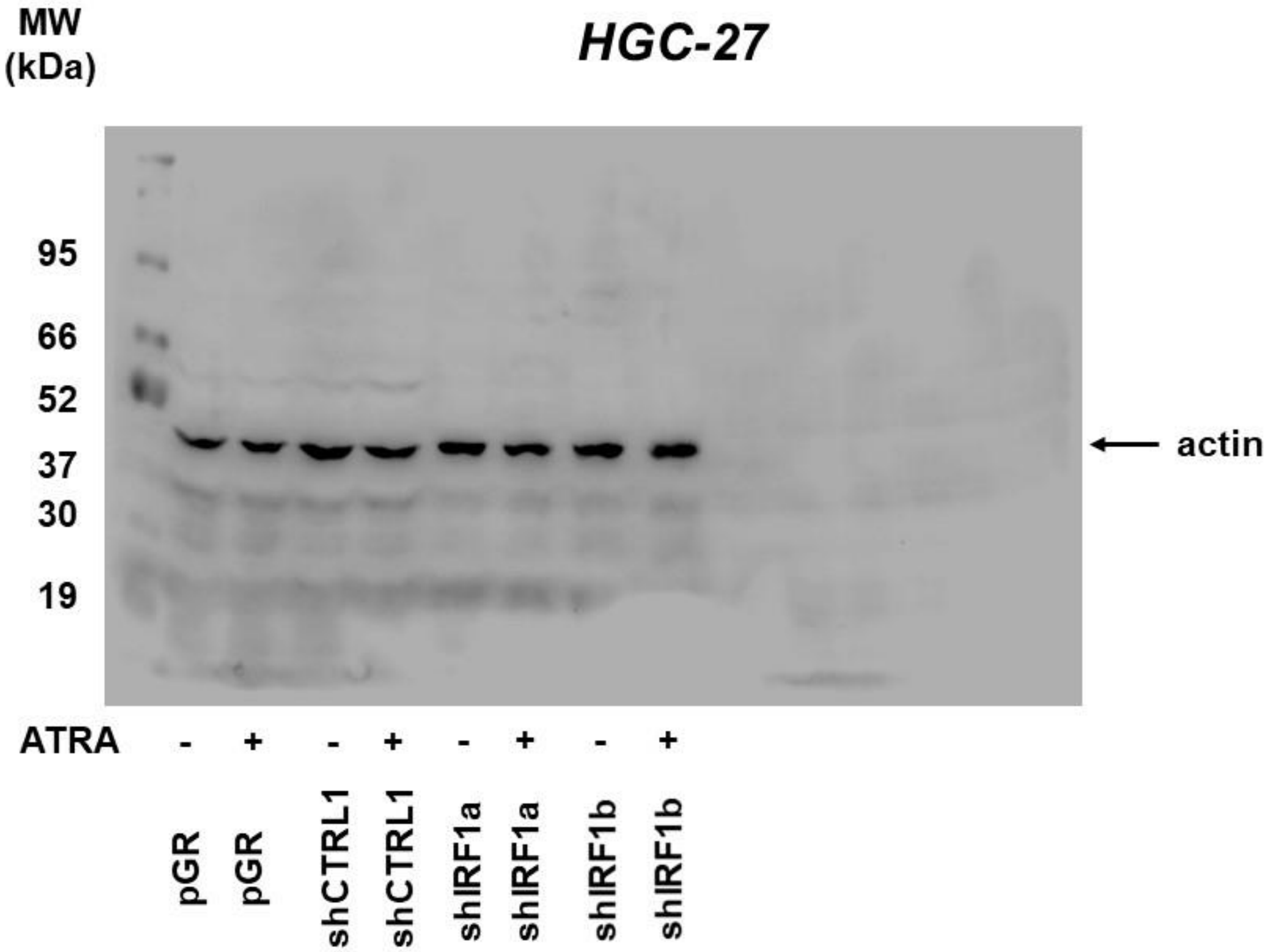

Original **Fig 8C**

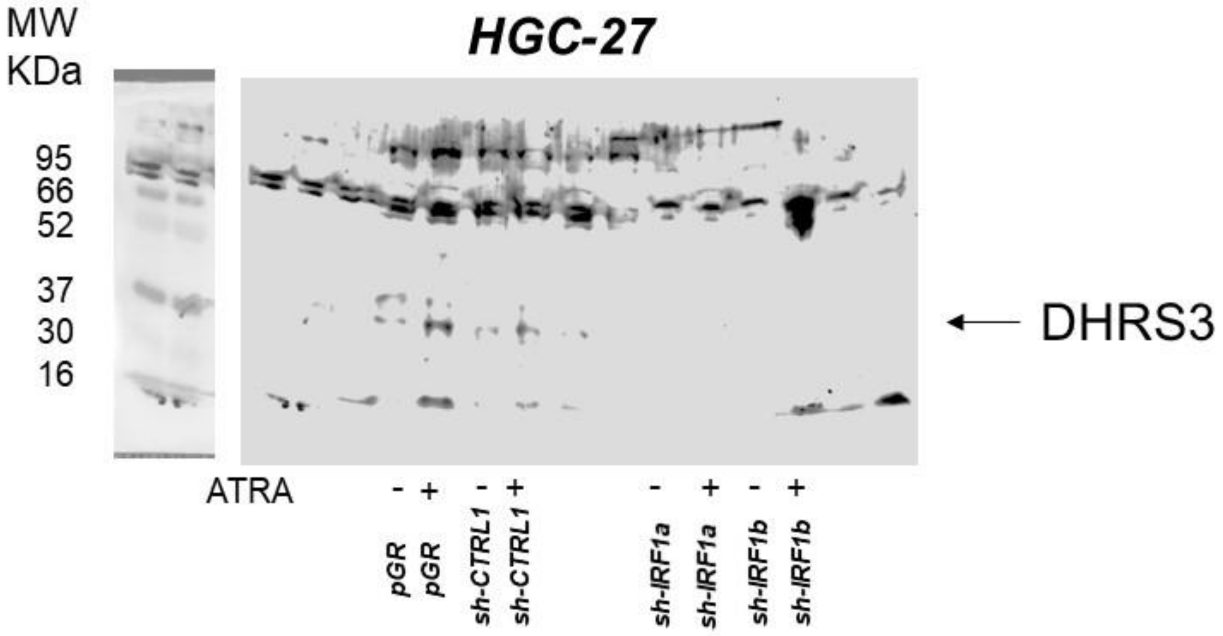

Original **Fig 8C**

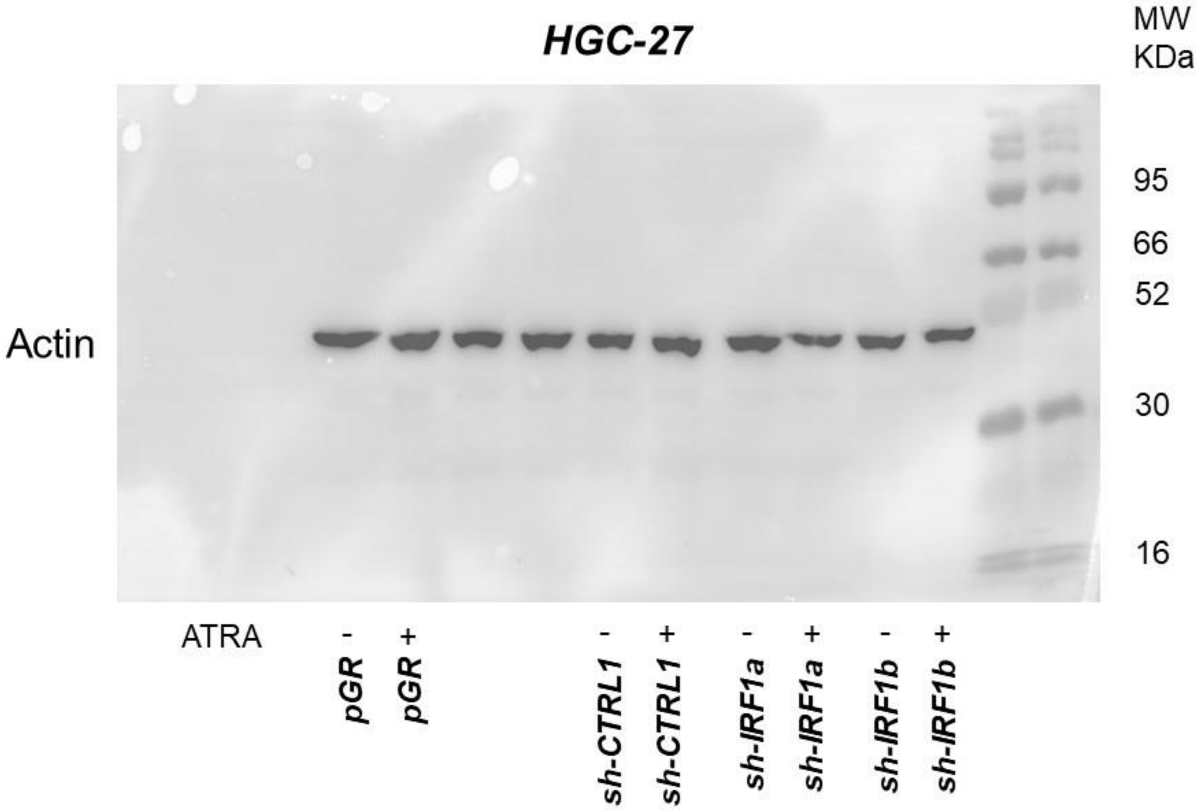

Original **Fig 8C**

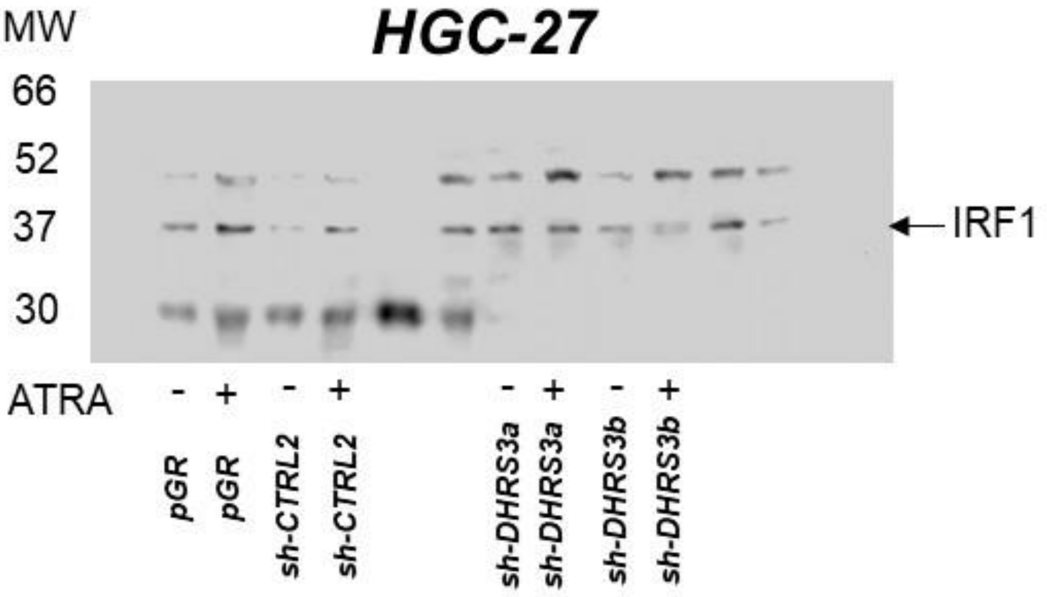

Original **Fig 8E**

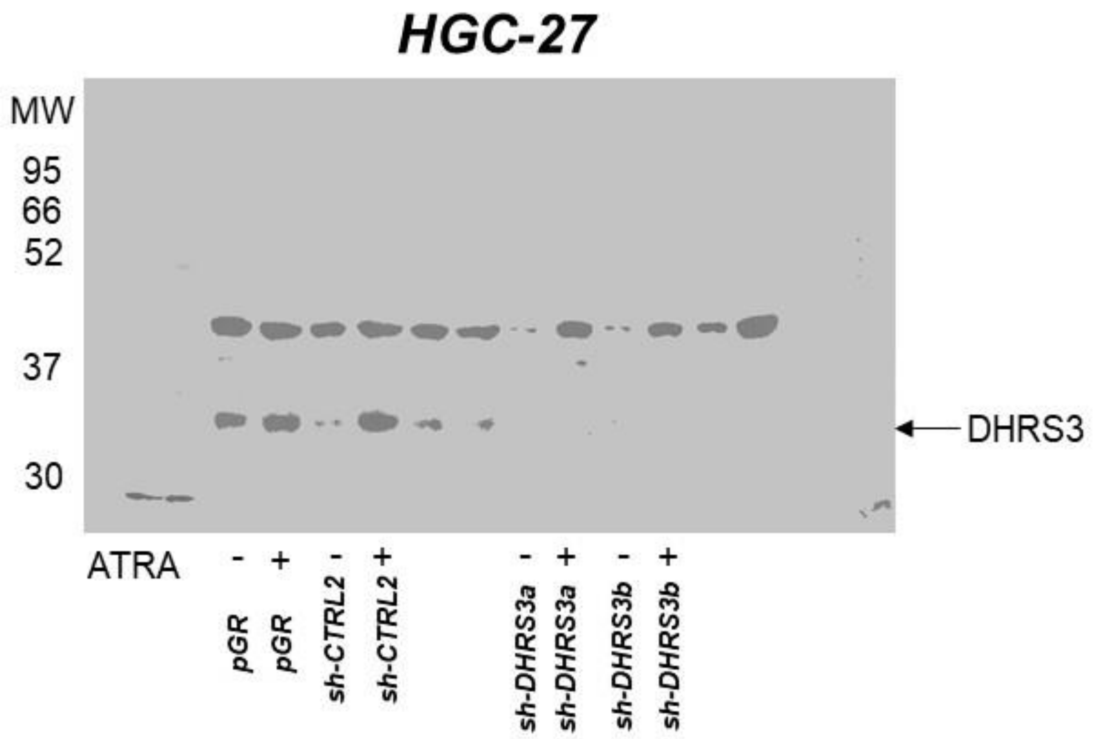

Original **Fig 8E**

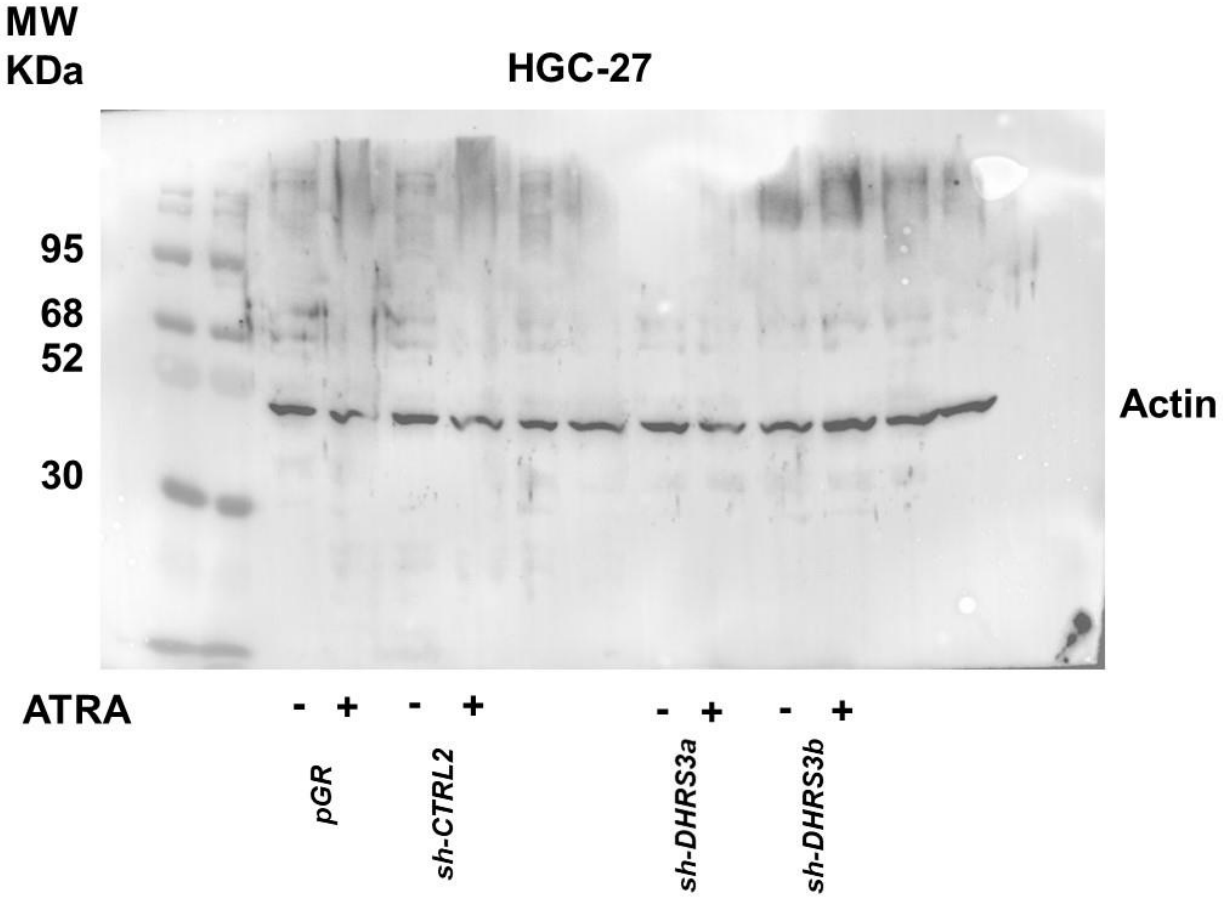

Original **Fig 8E**

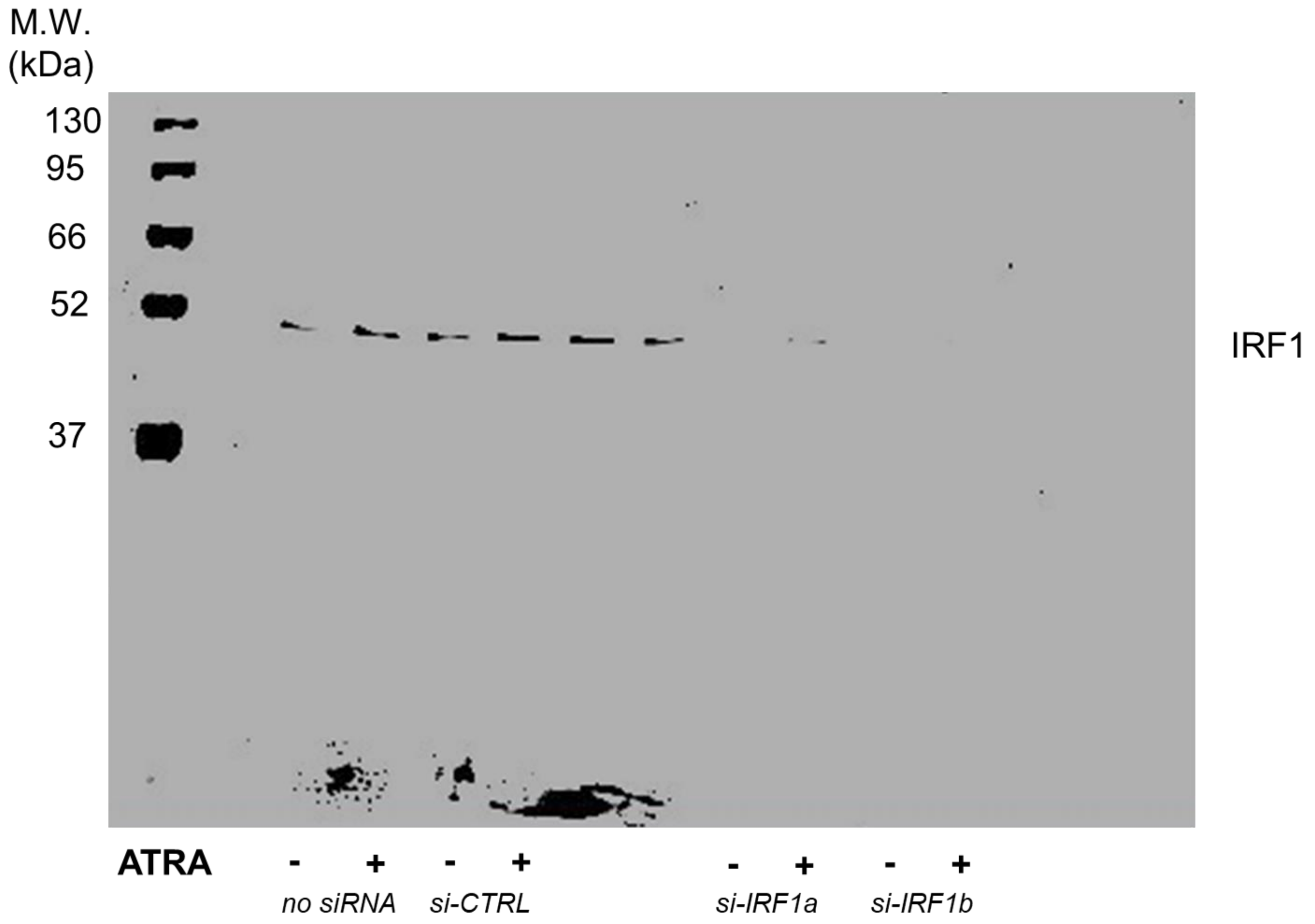

Original **Fig S7**

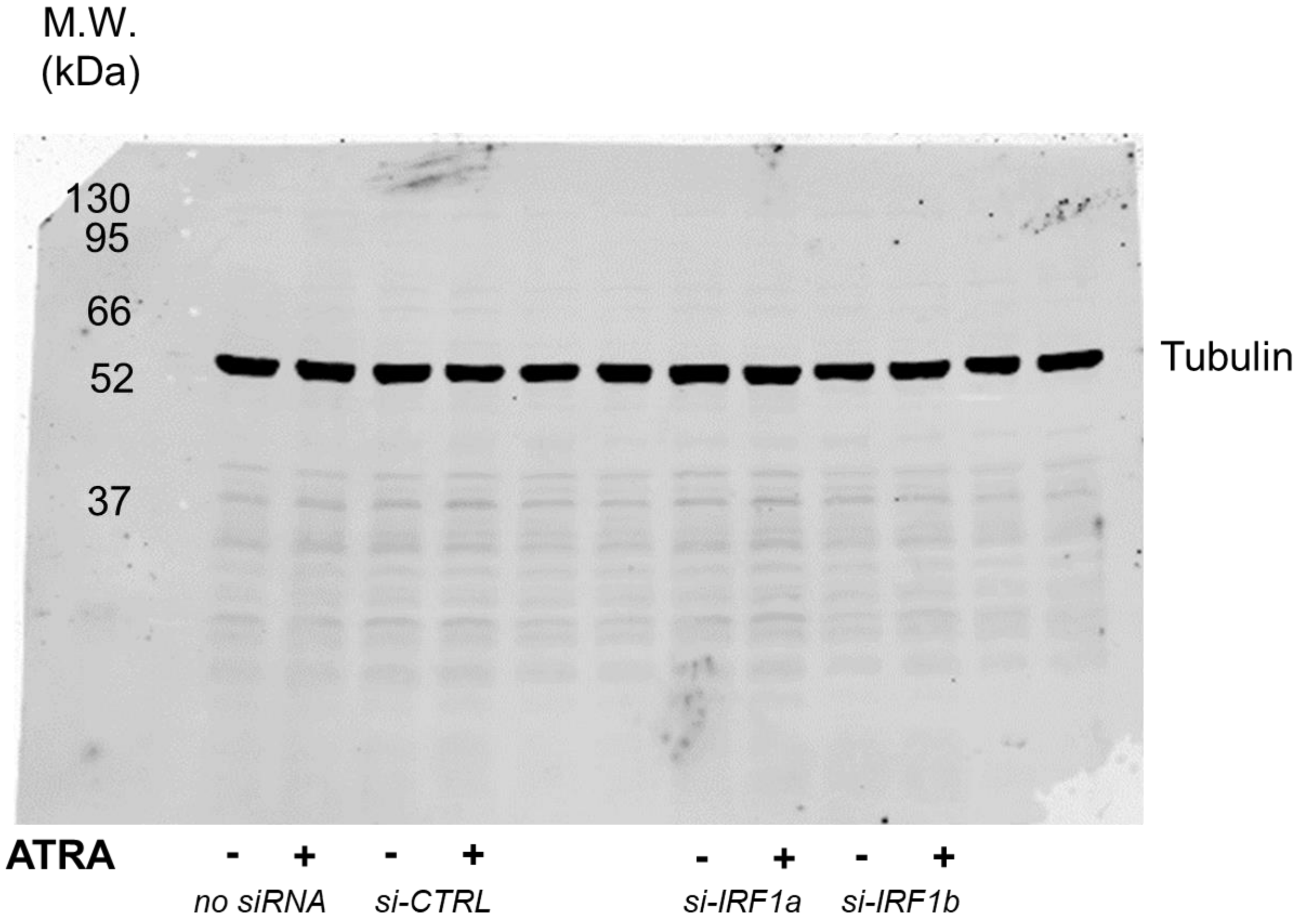

Original **Fig S7**

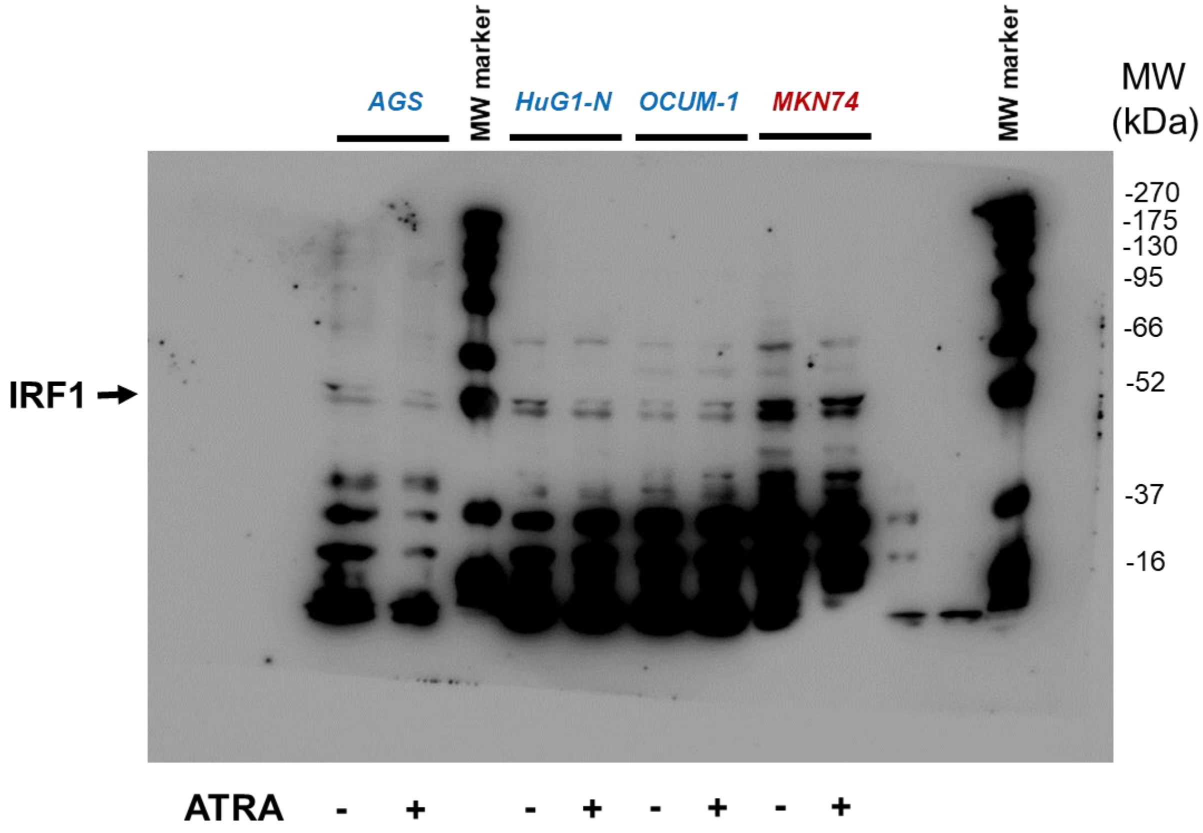

Original **Fig S8A**

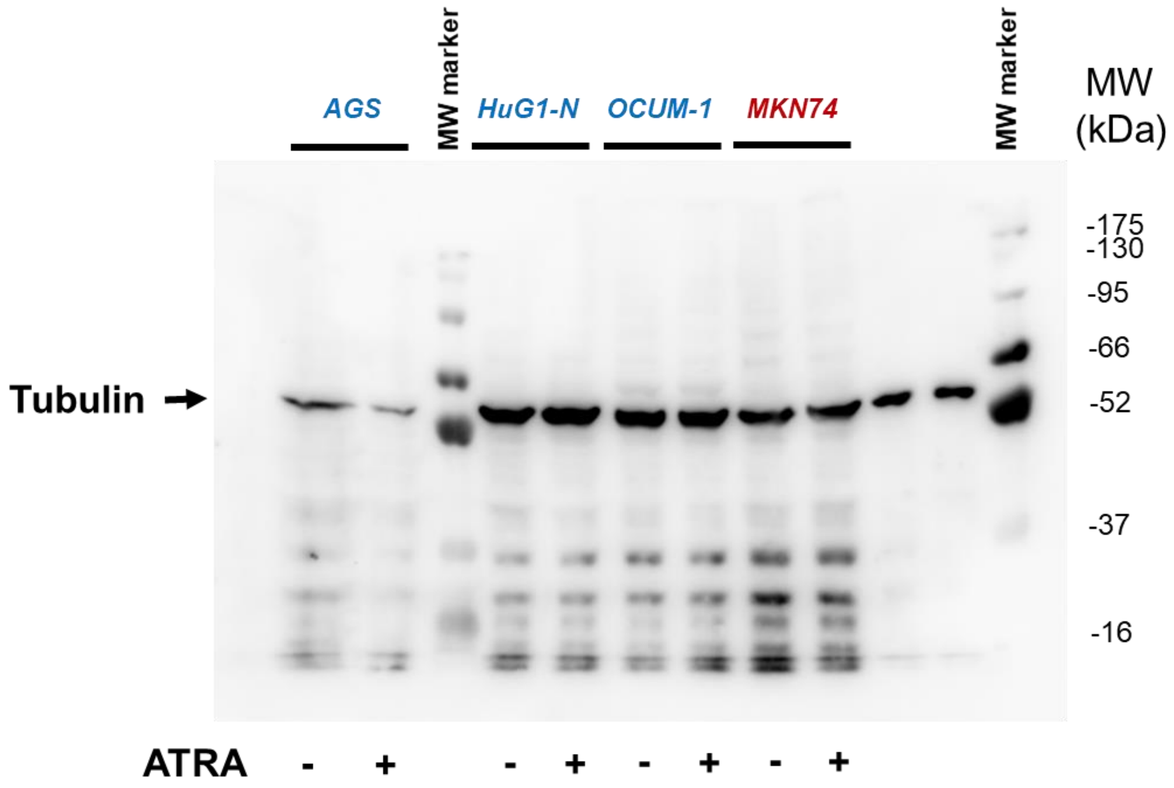

Original **Fig S8A**

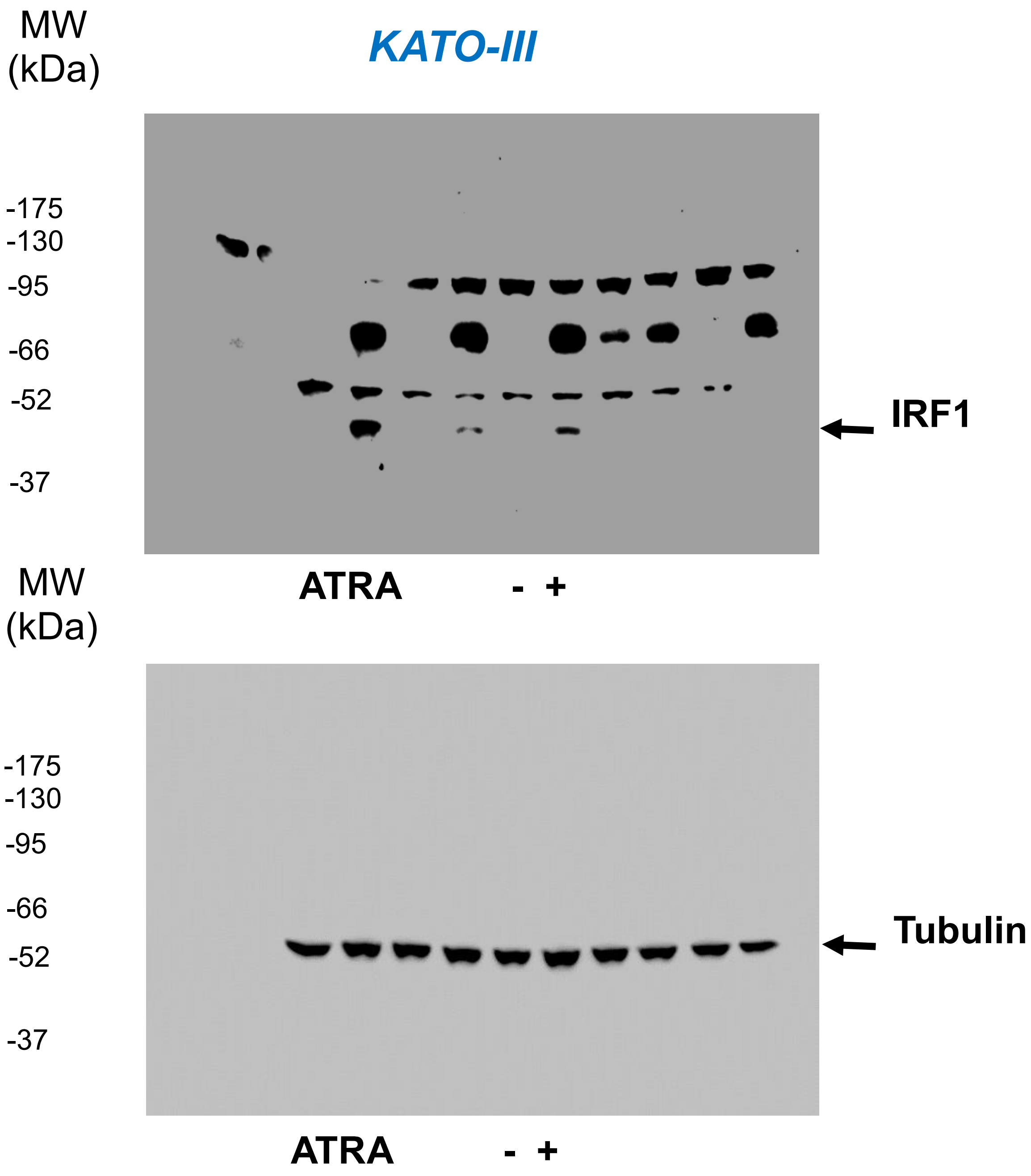

Original **Fig S8A**
